# An exploratory analysis of the response to ChAdOx1 nCoV-19 (AZD1222) vaccine in males and females

**DOI:** 10.1016/j.ebiom.2022.104128

**Published:** 2022-06-30

**Authors:** Natalie Gabrielle Marchevsky, Grace Li, Parvinder Aley, Sue Ann Costa Clemens, Jordan Richard Barrett, Sandra Belij-Rammerstorfer, Sagida Bibi, Elizabeth Clutterbuck, Christina Dold, Sally Felle, Amy Flaxman, Pedro Folegatti, Daniel Jenkin, Sarah Gilbert, Sarah Kelly, Teresa Lambe, Emma Plested, Maheshi Ramasamy, Nisha Singh, Holly Smith, Stephen Taylor, Lily Weckx, Andrew John Pollard, Merryn Voysey, the Oxford COVID Vaccine Trial Group

**Affiliations:** a Oxford Vaccine Group, Department of Paediatrics, University of Oxford, Oxford, UK; b Institute of Global Health, University of Siena, Siena, Italy; c Jenner Institute, University of Oxford, Oxford, UK; d Oxford Vaccine Group, Department of Paediatrics, University of Oxford, Oxford, UK and Chinese Academy of Medical Science (CAMS) Oxford Institute, University of Oxford, Oxford, UK; e National Infection Service, Public Health England, Salisbury, UK; f Department of Pediatrics, Universidade Federal de São Paulo, São Paulo, Brazil; g Oxford NIHR Biomedical Research Centre, Oxford, UK

**Keywords:** Vaccination, COVID-19, Sex-differences, Clinical trials

## Abstract

**Background:**

There are known differences in vaccine reactogenicity and immunogenicity by sex. Females have been shown to report greater reactogenicity and generate higher humoral and cellular immune responses than males following vaccination with several different vaccines. Whether this is also the case for COVID-19 vaccines is currently unknown, as COVID-19 vaccine study data disaggregated by sex are not routinely reported. Therefore, we have assessed the influence of sex on reactogenicity, immunogenicity and efficacy of COVID-19 vaccine ChAdOx1 nCoV-19.

**Methods:**

Vaccine efficacy was assessed in 15169 volunteers enrolled into single-blind randomised controlled trials of ChAdOx1 nCoV-19 in Brazil and the UK, with the primary endpoint defined as nucleic acid amplification test (NAAT)-positive symptomatic SARS-CoV-2 infection. All participants were electronically randomised to receive two standard doses of vaccine or the control product. Logistic regression models were fitted to explore the effect of age and sex on reactogenicity, and linear models fitted to log-transformed values for immunogenicity data. Reactogenicity data were taken from self-reported diaries of 788 trial participants. Pseudovirus neutralisation assay data were available from 748 participants and anti-SARS-CoV-2 spike IgG assay data from 1543 participants.

**Findings:**

7619 participants received ChAdOx1 nCoV-19 and 7550 received the control. Vaccine efficacy in participants after two doses of ChAdOx1 nCoV-19 (4243 females and 3376 males) was 66.1% (95% CI 55.9-73.9%) in males and 59.9% (95% CI 49.8-67.9%) in females; with no evidence of a difference in efficacy between the sexes (vaccine by sex interaction term P=0.3359). A small, statistically significant difference in anti-spike IgG was observed (adjusted GMR 1.14; 95% CI 1.04-1.26), with higher titres in females than males, but there were no statistically significant differences in other immunological endpoints. Whilst the majority of individuals reported at least one systemic reaction following a first dose of ChAdOx1 nCoV-19, females were twice as likely as males to report any systemic reaction after a first dose (OR 1.95; 95% CI 1.37-2.77). Measured fever of 38°C or above was reported in 5% of females and 1% of males following first doses. Headache and fatigue were the most commonly reported reactions in both sexes.

**Interpretation:**

Our results show that there is no evidence of difference in efficacy of the COVID-19 vaccine ChAdOx1 nCoV-19 in males and females. Greater reactogenicity in females was not associated with any difference in vaccine efficacy.

**Funding:**

Studies were registered with ISRCTN 90906759 (COV002) and ISRCTN 89951424 (COV003) and follow-up is ongoing. Funding was received from the UK Research and Innovation, Engineering and Physical Sciences Research Council, National Institute for Health Research, Coalition for Epidemic Preparedness Innovations, National Institute for Health Research Oxford Biomedical Research Centre, Chinese Academy of Medical Sciences Innovation Fund for Medical Science, Thames Valley and South Midlands NIHR Clinical Research Network, the Lemann Foundation, Rede D'Or, the Brava and Telles Foundation, the Coordenacao de Aperfeicoamento de Pessoal de Nivel Superior, Brazil, and AstraZeneca.

**Research in context**

**Evidence before this study**

Sex differences in reactogenicity and immunogenicity in response to vaccines have previously been described for several vaccines. Females have been shown to repeatedly report greater reactogenicity and generate greater immune responses than males. The clinical relevance of higher immune responses in females is unclear as clinical data disaggregated by sex are infrequently reported and the quality of reported data on sex-differences is often poor. Whether this is also true for COVID-19 vaccine ChAdOx1 nCoV-19 is unclear.

Phase 2/3 and real-world data for BNT162b2 (Pfizer/BioNTech), mRNA-1273 (Moderna) and Ad26.COV2-S (Janssen) vaccines describe greater reactogenicity in females than males. Phase 3 efficacy studies of the BNT162b2, mRNA-1273 and AD26.COV2-S vaccines have shown similar efficacy in males and females. Whether greater reactogenicity to COVID-19 vaccines in females is associated with any differences in immunogenicity or efficacy has not been evaluated.

**Added value of this study**

This study assessed reactogenicity, immunogenicity and efficacy of COVID-19 vaccine ChAdOx1 nCOV-19, using data from randomised controlled studies of the vaccine in the UK and Brazil, disaggregated by sex.

**Implications of all the available evidence**

Our results show that although females had higher reactogenicity than males, there was no evidence of a difference in efficacy of the COVID-19 vaccine ChAdOx1 nCoV-19 in males and females. Only one immune readout showed a higher response in females than males.

Alt-text: Unlabelled box

## Introduction

Biological sex is known to impact immune responses, susceptibility to pathogens and outcome of infection.^1^ Prior to the COVID-19 pandemic it was recognised that differences existed between the sexes regarding risk of disease and clinical outcome from respiratory viral infections. Higher disease incidence and case fatality rates have been reported in males than females for Middle East Respiratory Syndrome (MERS)^2^ and males have poorer outcomes from Severe Acute Respiratory Syndrome (SARS).^3^ To evaluate sex-differences in clinical outcome in the current COVID-19 pandemic, the Global Health 50-50 collaboration has collected sex-disaggregated data on COVID-19 incidence and outcomes from 107 countries.^4^ Their results show that mortality from acute COVID-19 disease is higher in men; on average, 15 males have died for every 10 females during the pandemic. The World Health Organisation (WHO) has noted that a fuller understanding of the sex and gender considerations in the research, development and delivery of COVID-19 vaccines will be critical to the success of the global vaccination programme.^5^

Female sex has been associated with both greater reactogenicity and higher levels of humoral and cellular immunity in response to vaccination with non-COVID-19 vaccines, including those against influenza, yellow fever, pneumococcus and hepatitis B across a range of vaccine types.^6^^,^^7^ The clinical relevance of higher immune responses to vaccination in females is unclear as higher vaccine efficacy in females has not been widely reported for large vaccine efficacy trials and the quality of reported data on sex-differences is often poor.^8^

Studies suggest that sex differences also exist with regards to COVID-19 vaccine responses. Data from the COVID Symptom Study app developed by ZOE Global suggest that females are more likely to report both local and systemic reactions in response to COVID-19 vaccination with two doses of either BNT162b2 (Pfizer/BioNtech) or ChAdOx1 nCoV-19 than males,^9^ in contrast to clinical trial data which suggested no difference in reactogenicity between the sexes.^10^ To assess differences in responses to ChAdOx1 nCoV-19 by sex, we analysed reactogenicity, immunogenicity, and vaccine efficacy in randomised controlled studies of the vaccine in the UK and Brazil, disaggregated by sex.

## Methods

### Study design

Data were taken from two single-blind, randomised controlled efficacy trials in the UK (COV002) and Brazil (COV003). Between May 28 and December 8 2020, informed written consent from 21227 healthy volunteers was obtained, who were then recruited and vaccinated across both trials. 15169 were included in the primary efficacy analysis in this paper (7619 receiving ChAdOx1 nCoV-19 and 7550 receiving control vaccine). 5777 were participants in the UK study, and 9392 participated in the Brazil study. Health and social care workers and those considered at higher risk of exposure to SARS-CoV-2 were targeted for recruitment. The data cutoff date for COVID-19 cases to be included in this paper was February 28 2021.

The studies have been previously described and extensively published in detail, including study protocols.^11^^,^^12^

### Randomisation

**Figure 1:**
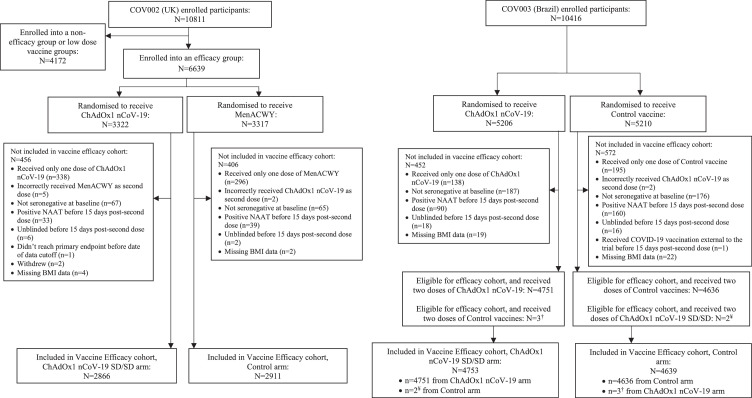
**CONSORT diagram for the efficacy cohort**. Chart shows numbers of participants enrolled and vaccinated in the COV002 (UK) study and the COV003 (Brazil) study. Shown are numbers who received each vaccine within each trial and reasons for exclusion from the efficacy analysis for each group.

Participants in the efficacy cohort (Figure 1) were randomised with full allocation concealment using a web-based randomisation system, in a ratio of 1:1 to receive two doses of ChAdOx1 nCoV-19 intramuscularly at 5 × 10^10^ vp, or two-doses of the control product (UK control group: 2 doses of MenACWY, Brazil control group: MenACWY as a first and saline as a second dose control). Computer randomisation was carried out within the secure web platform (REDCap v12.0.19) used for the electronic case report form. Study staff cleaned and checked data on an ongoing basis throughout the study. Randomisation lists, using block randomisation stratified by study group and site, were generated by the study statistician (MV). Participants were blinded to their allocation.

### Reactogenicity

A subset of participants were asked to complete a diary detailing solicited systemic and local adverse reactions for seven days after receiving their first and second doses of vaccine. Solicited systemic reactions included headache, fever (defined as an oral temperature of ≥38°C), feverishness, chills, muscle ache, joint pain, malaise, fatigue, and nausea; and solicited local reactions included pain, tenderness, redness, warmth, induration, itch, and swelling. Participants categorised their reactions as mild, moderate or severe using pre-specified criteria (Table S1). The reactogenicity cohorts consisted of trial participants who received two doses of ChAdOx1 nCoV-19 with at least one day of diary data in their first-dose or second-dose diaries. Participants were enrolled into the reactogenicity cohorts from a limited number of study sites, depending upon research capacity.

### Immunogenicity

The immunogenicity cohort consisted of participants who had received two doses of ChAdOx1 nCoV-19 and also had available anti-SARS-CoV-2 spike IgG and/or antibody neutralisation data at 28 days after receiving the second dose of vaccine. Only participants with no positive nucleic acid amplification test (NAAT) tests prior to this timepoint were included in the immunogenicity cohort. Participants were removed from the immunogenicity cohort if they were unblinded or received any COVID-19 vaccinations outside of the trial prior to the 28 days post-second vaccination timepoint.

Baseline serum samples were measured for nucleocapsid reactivity with the Roche Elecsys Anti-SARS-CoV-2 serology assay and a multiplexed immunoassay used to assess the spike-specific response to ChAdOx1 nCoV-19 vaccination and/or natural SARS-CoV-2 infection. Antibody neutralisation was measured with a lentivirus-based pseudovirus particle expressing the SARS CoV-2 spike protein as previously described.^11^ Anti-spike antibody-dependent subclasses, isotypes and functions were measured using in-house assays as previously described.^13^

For comparison, convalescent plasma samples from non-trial participants were obtained from hospitalised patients with severe COVID-19 disease or healthcare workers with mild or asymptomatic infection. All participants were ≥18 years old and were enrolled into surveillance studies following a confirmed SARS-CoV-2 NAAT positive test.

## Outcomes

### Efficacy analysis

The primary efficacy outcome was symptomatic SARS-CoV-2 infection with positive NAAT from a nose and throat swab. Participants with at least one of the primary outcome symptoms (fever ≥ 37.8°C, shortness of breath, cough, anosmia or ageusia) were included as primary outcome cases. Only primary outcome cases occurring more than 14 days after receiving a second dose were eligible for inclusion in efficacy analysis. Participants with positive NAAT swabs within 14 days after receiving their second dose were excluded from the efficacy cohort. Other exclusions included participants who were not seronegative at baseline, those who discontinued from the study with a follow-up time of less than 15 days after the second vaccination, and those not enrolled in efficacy cohorts. All cases were reviewed for inclusion by an independent blinded outcome adjudication committee. Participants were analysed according to vaccine received.

The efficacy analysis presented here is an exploratory subgroup analysis by sex. There are similarities in the targeted recruitment populations for phase 3 cohorts in COV002 and COV003, therefore efficacy analyses have been combined from these two studies.

### Ethics

Written informed consent was obtained from all participants. Studies were approved by the following committees: Brazilian National Research Ethics Committee (reference 4068113); Gastrointestinal Illness in Oxford: COVID substudy (Sheffield Research Ethics Committee, reference 16/YH/0247); Health Research Authority and Health and Care Research Wales^20^^/SC/0179^; ISARIC/WHO Clinical Characterisation Protocol for Severe Emerging Infections (Oxford Research Ethics Committee C, reference 13/SC/0149); Oxford Tropical Research Ethics Committee (OxTREC Reference 36-20); Sepsis Immunomics Project (Oxford Research Ethics Committee C, reference 19/SC/0296); Studies were registered with ISRCTN 90906759 (COV002) and ISRCTN 89951424 (COV003).

An international data safety monitoring board reviewed the safety data for the studies.

### Statistical methods

Baseline characteristics of each cohort were summarised by sex and presented as frequencies (%) or medians with IQRs for categorical or continuous variables, respectively. Distribution of sex and health/social care worker status was additionally explored in participants aged 18-65 years (considered to be of working age). The number of average daily COVID-19 patient contacts amongst participants working in a health/social care setting was collected. Proportions with 95% binomial confidence intervals, and P-values from Chi-squared tests, are presented to explore associations by sex.

Vaccine efficacy (VE) was calculated as 1-aRR (the adjusted relative risk; ChAdOx1 nCoV-19 vs control) computed using a robust Poisson regression model. The model contained terms for sex, country, vaccine received, BMI (linear and quadratic terms), health/social care worker status (yes/no), ethnicity (white/non-white), and age at randomisation (linear and quadratic terms). The model additionally contained interaction terms for age by health/social care worker status, and vaccine group by sex. The logarithm of the period at risk was used as an offset variable in the model to adjust for volunteers experiencing events at varying follow-up times. Participants were censored at the earliest occurrence of positive NAAT swab, discontinuation/withdrawal, unblinding, receiving a COVID-19 vaccination external to the trials, or data cutoff date. Severity of disease in primary symptomatic cases, measured by the WHO severity grading scale, was explored by sex using the Cochran-Armitage test for trend.

Immune responses at 28 days post-second vaccination have been summarised as medians and IQRs, and geometric mean titres (GMTs) with 95% CIs for log-transformed data. Geometric mean ratios (GMRs) with 95% CIs and associated P-values, from Student's t-tests, are presented for comparison between females and males. Adjusted linear models of log-transformed immune responses were fit to produce GMRs adjusted for age at randomisation (continuous, linear term only), country, health/social care worker status, ethnicity, and the interaction between age and health/social care worker status. Anti-spike antibody isotypes, subclasses and function data were compared between sexes using Bonferroni-adjusted P-values from Wilcoxon rank-sum tests. All other P-values are unadjusted for multiplicity.

Levels of receptor binding domain (RBD), anti-spike, and anti-nucleocapsid antibodies in plasma samples from convalescent patients are presented by reported WHO severity grading scale rating. Linear models were fitted to assess the differences between males and females on each antibody response using the first sample taken for each patient, adjusting for severity of disease and age of patient.

Solicited adverse events are presented as frequencies (%) with 95% binomial exact confidence intervals. Logistic regression models were fitted as exploratory analyses to observe the effect of age and sex on adverse events after first and second ChAdOx1 nCoV-19 vaccinations. For interpretation purposes, age was fit as a continuous variable, centered at 18 (the lower age bound of participants recruited to the studies). Models contained additional terms to adjust for the potentially confounding effects of country, health/social care worker status and ethnicity. Correlation matrices were produced to assess the associations between solicited adverse events in each sex.

In all regression models, linearity assumptions for continuous variables were checked and transformations made as appropriate. A statistical analysis plan was prepared prior to the first efficacy analysis.

### Sample size

Formal sample size calculations for efficacy were pre-specified for the main and interim efficacy analysis as previously published,^14^ but not for subgroup analyses.

There was no formal sample size calculation for immunogenicity outcomes as these were secondary or exploratory. Anti-spike IgG titres were measured on a subset of 15% of trial participants as required for regulatory submission, whereas other assays were run on smaller subsets.

### Software

Data analysis was done using R version 3.6.1 or later and SAS version 9.4. Robust Poisson models were fitted using “*proc genmod*” function, and logistic models were fitted using “*proc logistic*” function, in SAS.

### Role of funders

Funders had no role in study design, data collection, data analyses, interpretation, or writing of this report.

## Results

### Efficacy

Baseline characteristics in the efficacy cohort were similar for vaccine and control groups (Table 1). Differences in age, BMI, ethnicity, co-morbidities, and health/social care worker status were noted between the sexes in this cohort. The female population was younger, with 8% of females aged over 65 years compared with 15% of males. BMI was higher in males in both vaccine and control arms, and greater ethnic diversity was seen among male participants (24% non-white males versus 21% non-white females). Males reported a higher prevalence of cardiovascular disease and diabetes, whereas females reported more pre-existing respiratory disease. Similar differences were seen across both studies (Table S2). The distribution of health/social care workers aged 18–65 years in the cohort differed by sex (*P*<0.0001, Chi-squared test; Table S3) with 64% of females working in health or social care, compared with 36% of males. Average daily contact with COVID-19 patients in health/social care workers was not statistically significantly different between the sexes (*P*=0.1191, Chi-squared test; Table S4). However, there were country-specific differences, as female health/social care workers on average made fewer daily contacts with COVID-19 patients than males in the UK (*P*=0.0001; Chi-squared test), but this was not so in Brazil (*P*=0.0583; Chi-squared test).

**Table 1:** Baseline characteristics of the efficacy analysis population, by sex.

	ChAdOx1 nCoV-19		Control	
	Female	Male	Female	Male
N	4243	3376	4150	3400
BMI, median [IQR]	24.8 [22.3, 28.6]	26.6 [24.2, 29.6]	25.1 [22.5, 28.9]	26.5 [24.1, 29.6]
Health/social care worker	2937 (69.2%)	1673 (49.6%)	2866 (69.1%)	1686 (49.6%)
Age (years), median [IQR]	41.0 [30.0, 53.0]	43.0 [32.0, 58.0]	41.0 [31.0, 53.2]	42.0 [32.0, 57.0]
Age categories:				
*18-25 years*	442 (10.4%)	311 (9.2%)	431 (10.4%)	289 (8.5%)
*26-35 years*	1128 (26.6%)	802 (23.8%)	1050 (25.3%)	833 (24.5%)
*36-45 years*	978 (23.0%)	776 (23.0%)	976 (23.5%)	809 (23.8%)
*46-55 years*	812 (19.1%)	513 (15.2%)	812 (19.6%)	535 (15.7%)
*56-65 years*	522 (12.3%)	472 (14.0%)	532 (12.8%)	446 (13.1%)
*≥66 years*	361 (8.5%)	502 (14.9%)	349 (8.4%)	488 (14.4%)
Country (study):				
*UK (COV002)*	1596 (37.6%)	1270 (37.6%)	1650 (39.8%)	1261 (37.1%)
*Brazil (COV003)*	2647 (62.4%)	2106 (62.4%)	2500 (60.2%)	2139 (62.9%)
Ethnicity:				
*White*	3325 (78.4%)	2593 (76.8%)	3343 (80.6%)	2581 (75.9%)
*Black*	232 (5.5%)	195 (5.8%)	223 (5.4%)	200 (5.9%)
*Asian*	129 (3.0%)	139 (4.1%)	123 (3.0%)	121 (3.6%)
*Mixed*	541 (12.8%)	420 (12.4%)	436 (10.5%)	477 (14.0%)
*Other*	16 (0.4%)	29 (0.9%)	25 (0.6%)	21 (0.6%)
Co-morbidities:				
*Cardiovascular disease*	495 (11.7%)	710 (21.0%)	493 (11.9%)	707 (20.8%)
*Respiratory disease*	473 (11.1%)	357 (10.6%)	457 (11.0%)	353 (10.4%)
*Diabetes*	121 (2.9%)	189 (5.6%)	118 (2.8%)	140 (4.1%)

663 cases of primary symptomatic COVID-19 occurred more than 14 days after a second dose, 109 (2.6%) in the 4243 female participants in the ChAdOx1 nCoV-19 group and 261 (6.3%) in the 4150 female participants in the control group, with adjusted efficacy of 59.9% (95% CI 49.8–67.9%; Table 2). In the male participants, 75 (2.2%) cases occurred in the 3376 participants in the ChAdOx1 nCoV-19 group and 218 (6.4%) in the 3400 in the control group, with adjusted vaccine efficacy of 66.1% (95% CI 55.9–73.9%). The interaction term between vaccine arm and sex was not statistically significant (*P*=0.3359, robust Poisson regression). No statistically significant difference between males and females was found in the severity of breakthrough disease or disease in the control arm (*P*=0.1299 and *P*=0.1453, respectively; Cochran Armitage tests for trend; Table S5).

**Table 2:** Vaccine efficacy against primary symptomatic COVID-19 disease in females and males, primary efficacy population.

	Total cases	ChAdOx1 nCoV-19 n/N (%)	Control n/N (%)	Adjusted VE (95% CI)*	*P*-value (interaction)
Females (N=8393)	370	109/4243 (2.6%)	261/4150 (6.3%)	59.9% (49.8%, 67.9%)	0.3359
Males (N=6776)	293	75/3376 (2.2%)	218/3400 (6.4%)	66.1% (55.9%, 73.9%)	

⁎ Model adjusted for country (UK/Brazil), age, BMI, health/social care worker, ethnicity (white/non-white), as well as the interaction between age and health/social care worker status. Interaction P-value is from the interaction between sex and vaccine group.

### Immunogenicity

**Figure 2:**
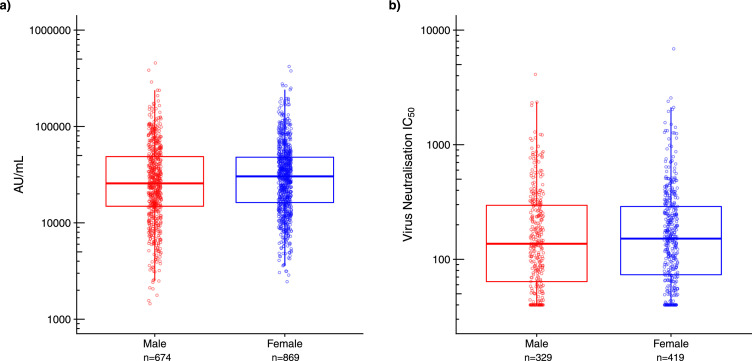
**Immune response 28 days after a second dose of ChAdOx1 nCoV-19 in females and males and females**. Boxplots represent the median and 25^th^ and 75^th^ percentiles; each data point is one participant. The number of participants included in each group is shown beneath each box plot. Summary statistics and further analysis of these data are provided in Table S7. a) Anti-SARS-CoV-2 spike IgG by multiplex immunoassay. P=0.0054 from linear model of log-transformed antibody values adjusting for country, age, health/social care worker, ethnicity, and interaction between age and health/social care worker. AU/mL: Arbitrary Units per millilitre; b) Neutralisation titres by pseudovirus assay. P=0.2795 from linear model of log-transformed antibody values adjusting for country, age, health/social care worker, ethnicity, and interaction between age and health/social care worker. IC_50_: concentration achieving 50% inhibition of viral replication.

**Figure 3:**
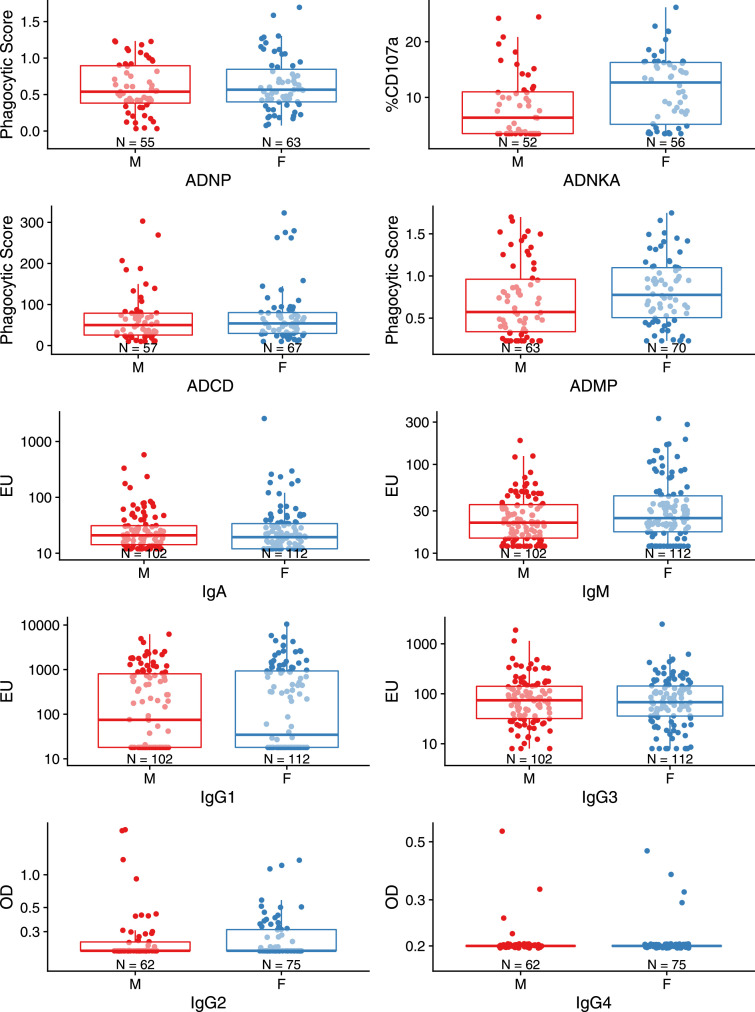
**Anti-spike antibody isotypes, subclasses and function in males and females at day 28 after dose 2**. ADCD: antibody dependent complement deposition; ADMP: antibody dependent monocyte phagocytosis; ADNKA: antibody dependent natural killer cell activation; ADNP: antibody dependent neutrophil phagocytosis. EU: Elisa Units; OD: optical density; Ig: Immunoglobulin. Comparisons of normalized data between males (M) and females (F) by Wilcoxon rank sum tests: all Bonferroni-adjusted P-values all >0.05. Boxplots represent the median and 25th and 75th percentiles; each data point is one participant. The number of participants included in each analysis is shown beneath each box plot. Numbers of samples were limited by the experimental size of the assays and laboratory capacity. Summary Statistics provided in Table S8.

A total of 1543 participants (973 UK, 570 Brazil) had anti-SARS-CoV-2 spike IgG data, and 748 participants (484 UK, 264 Brazil) had available pseudovirus neutralising antibody data (Table S6). Anti-spike IgG responses 28 days after a second ChAdOx1 nCoV-19 vaccination were statistically significantly higher in females than males, both before and after adjustment for potential confounders (adjusted GMR 1.14; 95% CI 1.04–1.26; Table S7). However, neutralising antibody levels were similar between the sexes (Figure 2). Adjusting for potentially confounding variables, there was no statistically significant difference between males and females in neutralising antibody response to ChAdOx1 nCoV-19 vaccine (adjusted GMR 1.08; 95% CI 0.94-1.24). Differences in antibody subclasses, isotypes and functions were not statistically significant between males and females (Figure 3, Table S8).

Convalescent plasma samples were available from 39 unvaccinated males and 74 females. A higher proportion of males had severe disease (67%) compared with females (19%). The majority of samples were collected from health/social care workers, the majority being female (82%), with asymptomatic and mild disease. The median age of males with convalescent plasma samples was 57 years (IQR 47–68 years) compared to 43 years (IQR 30–53 years) in females. Adjusting for both age and severity of disease, there were no statistically significant differences between males and females with regards to RBD, nucleocapsid, or spike antibody response to COVID-19 infection (*P*>0.5000 in each linear regression model; Figure S1).

### Reactogenicity

The first-dose reactogenicity cohort included 788 participants (708 from the UK and 80 from Brazil) with diary data for their first ChAdOx1 nCoV-19 vaccination, and 676 participants in the second-dose cohort (633 UK participants, 43 Brazilian participants).

**Figure 4:**
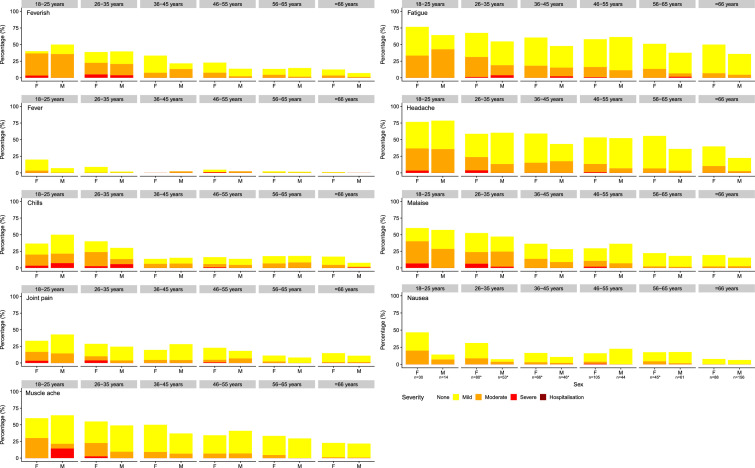
**Solicited systemic reactions after a first dose of ChAdOx1 nCoV-19 in participants returning diary data, by sex and age at vaccination**. The numbers of female and male participants included in each age group are shown on the final reaction panel (nausea). These numbers apply for all solicited symptoms presented in this figure except where indicated with *, where the denominator is n=5 fewer participants for the fever panel due to missing temperature readings. Data presented are maximum symptom severity reported over the first 0-7 days following a first dose of ChAdOx1 nCoV-19. Participants categorised the severity of their reactions using pre-specified criteria (Table S1). F: females; M: males.

**Figure 5:**
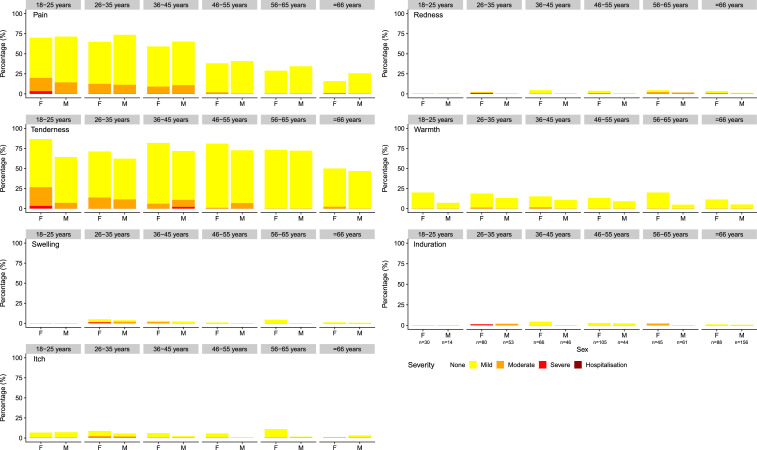
**Solicited local reactions after a first dose of ChAdOx1 nCoV-19 in participants returning diary data, by sex and age at vaccination**. The numbers of female and male participants included in each age group are shown on the final reaction panel (induration). These numbers apply for all solicited symptoms presented in this figure. Data presented are maximum severity reported over the first 0-7 days following a first dose of ChAdOx1 nCoV-19. Participants categorised the severity of their reactions using pre-specified criteria (Table S1). F: females; M: males.

Reported fever rates were higher in females (5%) than males (1%) after first doses. For each 10-year increase from 18 years of age, the odds of having any solicited systemic reaction after a first vaccination decreased by 35% (adjusted OR 0.65; 95% CI 0.56–0.75), and by 48% (adjusted OR 0.52; 95% CI 0.44–0.62) for any solicited local reaction (Table 3, Tables S9,S10; Figures 4,5). This trend of decreasing odds with increasing age was seen for all solicited adverse reactions after second vaccination, despite a reduction in severity and frequency of reported adverse events after the second dose (Tables S11,S12; Figures S2,S3).

**Table 3:** Analysis of solicited local or systemic adverse events in 7 days after ChAdOx1 nCoV-19 first or second vaccination.

Outcome	Effect	Adjusted ORs (95% CI)*	*P*-value
First dose: any systemic event	Sex (female vs male)	1.95 (1.37, 2.77)	0.0002
	Age (per 10-year increase in age)	0.65 (0.56, 0.75)	<0.0001
First dose: any local event	Sex (female vs male)	0.90 (0.62, 1.31)	0.5819
	Age (per 10-year increase in age)	0.52 (0.44, 0.62)	<0.0001
Second dose: any systemic event	Sex (female vs male)	1.23 (0.88, 1.71)	0.2315
	Age (per 10-year increase in age)	0.76 (0.68, 0.86)	<0.0001
Second dose: any local event	Sex (female vs male)	1.79 (1.27, 2.51)	0.0008
	Age (per 10-year increase in age)	0.72 (0.64, 0.82)	<0.0001
First dose: any moderate/severe systemic event	Sex (female vs male)	1.51 (1.05, 2.17)	0.0247
	Age (per 10-year increase in age)	0.59 (0.52, 0.66)	<0.0001
First dose: any moderate/severe local event	Sex (female vs male)	1.19 (0.66, 2.13)	0.5644
	Age (per 10-year increase in age)	0.44 (0.35, 0.54)	<0.0001
Second dose: any moderate/severe systemic event	Sex (female vs male)	1.38 (0.84, 2.25)	0.2017
	Age (per 10-year increase in age)	0.68 (0.58, 0.79)	<0.0001
Second dose: any moderate/severe local event	Sex (female vs male)	1.38 (0.50, 3.79)	0.5362
	Age (per 10-year increase in age)	0.70 (0.51, 0.97)	0.0309

⁎ Models adjusted for country (UK/Brazil), health/social care worker, and ethnicity (white/non-white). Age has been centered on 18 years and modelled such that the adjusted odds ratio corresponds to the change associated with being 10 years older. Data is from participants who completed diaries.

Adjusting for age and other potentially confounding variables, females had almost twice the odds of experiencing a systemic reaction after first vaccination than males (adjusted OR 1.95; 95% CI 1.37–2.77). Solicited systemic adverse reactions did not differ between males and females after a second dose; however, females had increased odds of a local reaction after a second dose (adjusted OR 1.79; 95% CI 1.27–2.51). Interaction terms for age by sex and age by health/social care worker were explored, but were statistically insignificant in all models. Despite differences in frequency of reactions, both sexes reported fatigue and tenderness as the most common solicited systemic and local adverse reactions, respectively, after both first and second ChAdOx1 nCoV-19 doses (Tables S13,S14). No differences between females and males were seen in correlations between solicited adverse reactions after either dose of ChAdOx1 nCoV-19 (Figures S4,S5).

## Discussion

This study shows that there is no evidence of a difference in efficacy of COVID-19 vaccine ChAdOx1 nCoV-19 between males and females. As observed for other vaccines, systemic reactions following the first dose were higher in females than males, and females were more likely to experience local reactions after a second dose. Whilst this trend was seen across age groups, reactogenicity in both sexes decreased with increasing age. Minor differences were also observed in binding antibody titres, which were higher in females, however this was not associated with any statistically significant difference in vaccine efficacy.

In a large phase 3 efficacy study in the United States, ChAdOx1 nCoV-19 vaccine efficacy was similar in males and females.^15^ This is consistent with findings in phase 3 trials of other COVID-19 vaccines. The phase 3 study of another adenoviral-vector COVID-19 vaccine Ad26.COV2.S (Janssen) vaccine found no statistically significant difference in VE between males and females, with 230 cases of moderate to severe COVID-19 in males (VE 69.8%; 95% CI 58.9-78.2%), and 207 cases in females (VE 60.3%; 95% CI 46.0-71.2%).^16^ Similarly, no difference in BNT162b2 vaccine efficacy was reported with VE of 96.5% in males (95% CI 84.9-99.3%) versus 93.7% in females (95% CI 84.7-98.0%)^10^ nor for an mRNA-1273 (Moderna) vaccine with 93.5% (95% CI 79.2-98.0%) VE in females versus 95.5% (95% CI 81.5-98.9%) in males.^17^

This study reports COVID-19 vaccine immune responses disaggregated by sex, showing a small difference in anti-spike IgG between males and females 28 days after receiving a second dose. However, no statistically significant differences were seen in neutralizing antibody titres, antibody isotypes or functionality profiles between the biological sexes. The sample sizes for neutralising antibody and systems serology assays were smaller than for SARS CoV-2 anti-spike IgG assays, and this may have affected our ability to detect statistically significant differences in these measures. To date, there are few reports of differing antibody functionality by biological sex after vaccination, though some differences are reported for measles vaccine.^18^ Our data therefore suggest that the difference in IgG titres observed is not clinically relevant, as the rate of reporting of symptomatic COVID-19 cases following vaccination did not differ between males and females.

Higher antibody titres are more commonly measured in females than males following vaccination but with no clear demonstration that these differences result in clinically meaningful variation in protection.^8^ For example, hemagglutination inhibition antibody titres were twice as high in adult females than adult males following trivalent influenza vaccination (strains A/H1N1, A/H3N2 and B during winter 2004).^19^ However, this observation was not associated with any difference in the risk of medical visits or hospitalisation for influenza-like illness. Similarly, two meta-analyses of individual participant immunogenicity data in young children including routine DTP-IPV-Hib and PCV vaccinations also showed evidence of higher antibody titres in female children across several vaccine types.^20^^,^^21^ Again, no consistent evidence of higher vaccine failure rates in either boys or girls was seen, nor a statistically significant difference in the proportion of either sex reaching IgG thresholds for correlates of protection for pneumococcal disease, severe Hib disease or pertussis.^21^

Several mechanisms have been suggested to explain sex-differences in humoral responses to vaccination. Oestrogen receptors are present on lymphocytes and pre-menopausal adult females generally exhibit stronger immune responses than children, adult males or post-menopausal females.^22^ However, evidence of sex differences in vaccination in childhood suggests that genetic or epigenetic factors may also be relevant. Several genes on the X-chromosome regulate immune response, including TLR7, IL-2 and IL-3.^23^ Sex differences have also been observed in the immune response to acute SARS-CoV-2 infection,^24^ and kynurenic acid is one of several factors which has been implicated in sex-specific immune responses to COVID-19.^25^

The observation of greater reactogenicity in females following COVID-19 vaccination in our study is also seen for other COVID-19 vaccines. Both phase 2/3 clinical trial reports and real-world evidence gathered for BNT162b2, mRNA-1273 and Ad26.COV2-S^9^^,^^26^, ^27^, ^28^ describe greater reactogenicity in females than males, a pattern also observed with vaccines against other diseases including yellow fever, measles, mumps and rubella and influenza.^29^^,^^30^,^31^ Fatigue and headache are also the two systemic reactions reported more commonly in females than males, in response to H1N1 vaccination^30^^,^^32^ and both local and systemic reactions are reported more commonly in females after both intramuscular and intradermal inactivated trivalent influenza vaccination.^28^ Our analysis describing decreasing reactogenicity with age in both sexes is consistent with the age-related differences in reactogenicity reported for other COVID-19 vaccines.^10^^,^^16^^,^^17^ Although both reactogenicity and immunogenicity have been shown to be higher in females, investigating whether they are linked is beyond the scope of the paper.

We adjusted for potential confounders to minimise potential bias in the analysis. Our models adjusted for health/social care worker status as a greater proportion of health/social care workers were female, and have potentially higher exposure to SARS-CoV-2. Health care workers were also younger than non-health care workers and so adjustment for age was important. This analysis includes clinical trial data collected from mid-2020 to early 2021. During this period, the alpha SARS-CoV-2 variant was the dominant variant in circulation in the UK and the gamma variant was emerging in Brazil. The observations of this study are limited to the UK and Brazil settings, and may not be representative of other ethnic groups or geographic settings.

Our results show that there is no evidence of a difference in efficacy of the COVID-19 vaccine ChAdOx1 nCoV-19 in males and females, and efficacy is observed in both sexes. By disaggregating sex in our analysis of trial data we show that greater reactogenicity in females does not affect clinical vaccine efficacy.

## Contributors

AJP is chief investigator. PA, SACC, SB-R, SB, CD, EAC, SF, HS, AF, PF, DJ, SG, JB, MR, SK, TL, EP, NS, ST, LW contributed to implementation of the study and/or laboratory experimentation. MV and NGM performed the statistical analysis and had full access to and verified the data. GL, NGM, AJP, and MV contributed to the preparation of the report. All authors critically reviewed and approved the final version.

## Data sharing statement

Anonymised participant data will be made available when the trials are complete, upon requests directed to the corresponding author. Proposals will be reviewed and approved by the sponsor, investigator, and collaborators on the basis of scientific merit. After approval of a proposal, data can be shared through a secure online platform after signing a data access agreement. All data will be made available for a minimum of 5 years from the end of the trial.

## Declaration of interests

Oxford University has entered into a partnership with AstraZeneca for further development of ChAdOx1 nCoV-19. AstraZeneca reviewed the final manuscript before submission, but the authors retained editorial control. SCG is a cofounder of and shareholder in Vaccitech (collaborators in the early development of this vaccine candidate) and named as an inventor on a patent covering use of ChAdOx1-vectored vaccines (PCT/GB2012/000467) and a patent application covering this SARS-CoV-2 vaccine. TL is named as an inventor on a patent covering use of ChAdOx1-vectored vaccines (PCT/GB2012/000467) and was a consultant to Vaccitech. PMF is a consultant to Vaccitech. AJP is Chair of the UK Department of Health and Social Care's JCVI, but does not participate in policy advice on coronavirus vaccines, and was a member of the WHO Strategic Advisory Group of Experts (SAGE) until 2022. MNR has acted as PI on commercial vaccine trials sponsored by Astra Zeneca and VBI vaccines but not personally received payment for this work. MV has received grants from the NIHR and Bill and Melinda Gates Foundation not related to this work and participated on Data Safety Monitoring Boards for NIHR funded projects not related to this paper.

## Appendix Supplementary materials

Image, application 1

Image, application 2

Image, application 3

Image, application 4

Image, application 5

Image, application 6

Image, application 7

Image, application 8
