# Supplementary material for: An exploratory analysis of the response to ChAdOx1 nCoV-19 (AZD1222) vaccine in males and females
Source: eBioMedicine. 2022 Jun 30;81:104128. doi: 10.1016/j.ebiom.2022.104128 (PMC9242842; doi:10.1016/j.ebiom.2022.104128)
Supplement: Supplementary file 1 [file mmc1.docx]

**Figure S1. Antibody response from aniti-SARS-CoV-2 multiplex immunoassay at PPD Laboratories in convalescent plasma samples, by disease severity and sex**

Figure contains RBD, nucleocapsid (N), and spike (S) IgG data for 39 males (M) (n=4 asymptomatic, n=9 mild, n=26 severe) and n=74 females (F) (n=17 asymptomatic, n=43 mild, n=14 severe) who were SARS-CoV-2 positive. Linear models were fit to log-transformed data to assess the effect of sex on each antibody response, adjusting for severity of disease and age of patient. Sex was insignificant in all models (p-values all >0.5000). AU/ml: Arbitrary units per millilitre. Boxplots represent the median and 25th and 75th percentiles. Each data point is one participant.

Figure S2. Solicited systemic reactions after a second dose of ChAdOx1 nCoV-19 recipients, by sex and age at vaccination.

The numbers of female and male participants included in each age group are shown on the final reaction panel (nausea). These numbers apply for all solicited symptoms presented in this figure except where indicated with *, where the denominator is n=5 fewer participants for the fever panel due to missing temperature readings.

Data presented are maximum symptom severity reported over the first 0-7 days following a first dose of ChAdOx1 nCoV-19. Participants categorised the severity of their reactions using pre-specified criteria in an e-diary (Table S1). F: females; M: males.

Figure S3. Solicited local reactions after a second dose of ChAdOx1 nCoV-19 recipients, by sex and age at vaccination.

The numbers of female and male participants included in each age group are shown on the final reaction panel (induration). These numbers apply for all solicited symptoms presented in this figure. Data presented are maximum symptom severity reported over the first 0-7 days following a first dose of ChAdOx1 nCoV-19. Participants categorised the severity of their reactions using pre-specified criteria in an e-diary (Table S1). F: females; M: males.

Figure S4. Correlation matrix of solicited adverse reactions after a first dose of ChAdOx1 nCoV-19.

This figure shows the correlations between each solicited adverse reaction based on a binary any (severity) [1]/none (no occurrence of symptom) [0] on days 0-7 following a first dose of ChAdOx1 nCoV-19, by sex. Symptom data is from 372 males and 411 females. Positive and negative correlations are represented by red and blue colours, respectively, where the intensity of the colour represents the strength of the correlations (see key included in figure). Pearson correlation coefficients (ranging from -1.0 to 1.0) for each pairwise association are also presented.

Figure S5. Correlation matrix of solicited adverse reactions after a second dose of ChAdOx1 nCoV-19.

This figure shows the correlations between each solicited adverse reaction based on a binary any (severity) [1]/none (no occurrence of symptom) [0] on days 0-7 following a second dose of ChAdOx1 nCoV-19, by sex. Symptom data is from 314 males and 349 females. Positive and negative correlations are represented by red and blue colours, respectively, where the intensity of the colour represents the strength of the correlations (see key included in figure). Pearson correlation coefficients (ranging from -1.0 to 1.0) for each pairwise association are also presented. Correlations have not been calculated for symptom combinations including fever or induration in males, as no males in the cohort experienced these symptoms after their second ChAdOx1 nCoV-19 dose.
