# Supplementary figures and images for: An exploratory analysis of the response to ChAdOx1 nCoV-19 (AZD1222) vaccine in males and females
Source: eBioMedicine. 2022 Jun 30;81:104128. doi: 10.1016/j.ebiom.2022.104128 (PMC9242842; doi:10.1016/j.ebiom.2022.104128)

# RBD

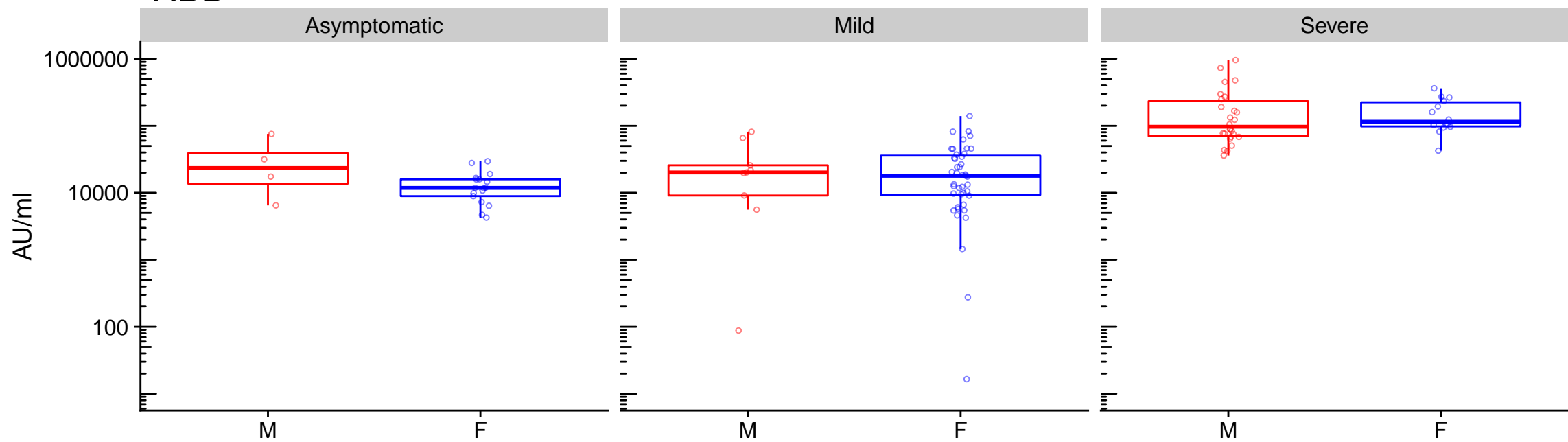

# N

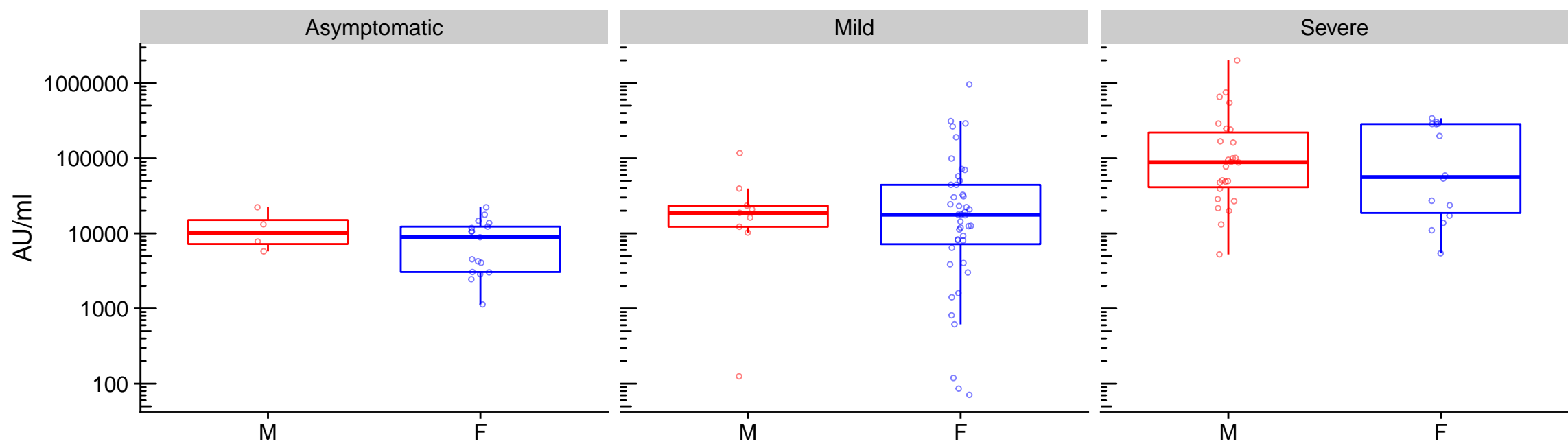

# S

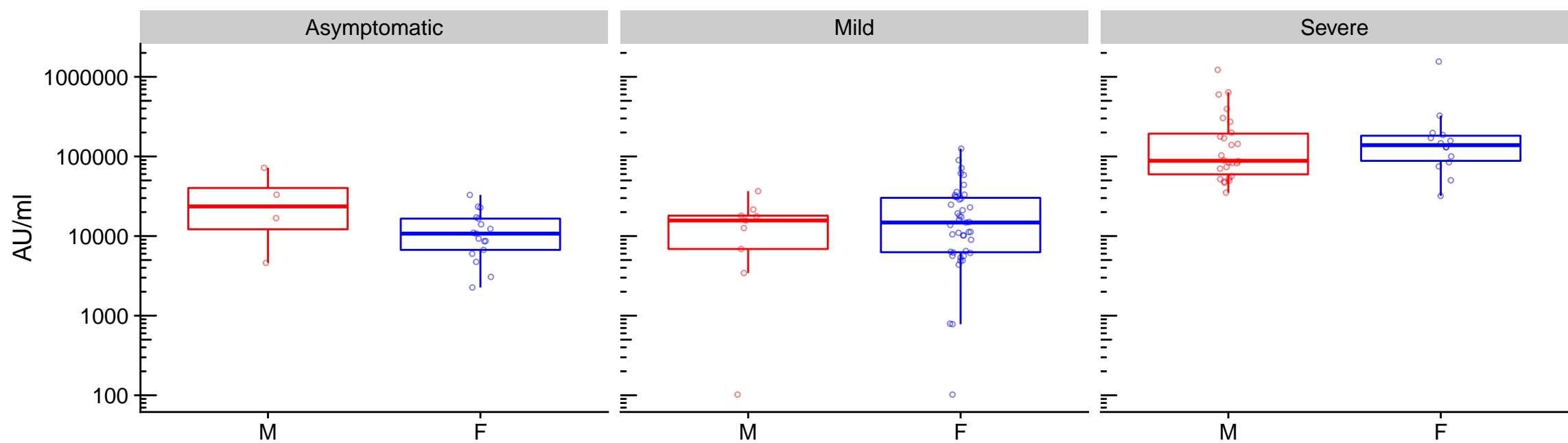

Supplement: Supplementary file 2 [file mmc2.pdf]

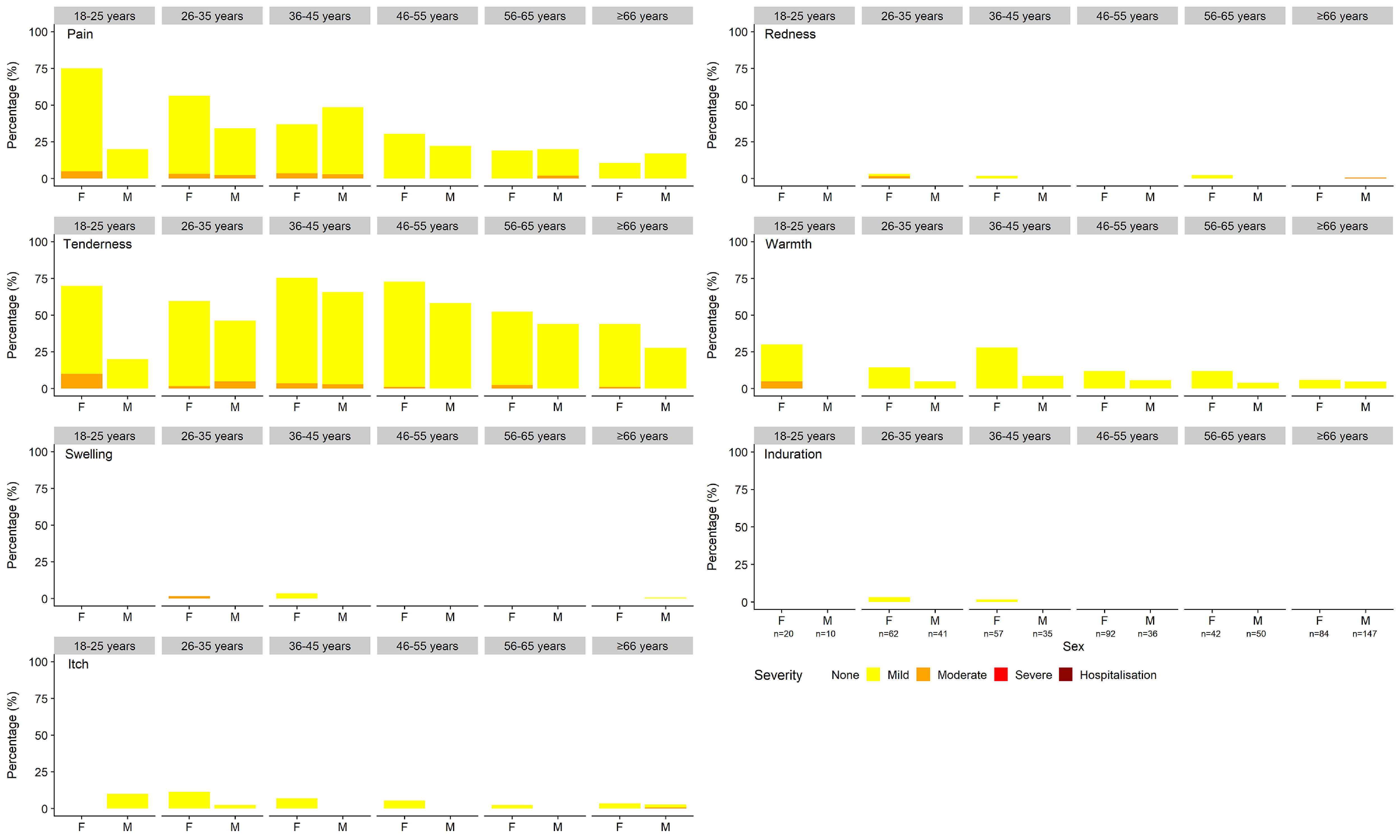

Supplement: Supplementary file 3 [file mmc3.jpg]
