# Supplementary material for: An exploratory analysis of the response to ChAdOx1 nCoV-19 (AZD1222) vaccine in males and females
Source: eBioMedicine. 2022 Jun 30;81:104128. doi: 10.1016/j.ebiom.2022.104128 (PMC9242842; doi:10.1016/j.ebiom.2022.104128)
Supplement: Supplementary file 5 [file mmc5.docx]

**Table S1. Severity grading scales for adverse events.**

| Grade 0 | None |
| --- | --- |
| Grade 1 | Mild: transient or mild discomfort (<48 hours); No interference with activity; No medical intervention/therapy required |
| Grade 2 | Moderate: mild to moderate limitation in activity – some assistance may be needed; no or minimal medical intervention/therapy required |
| Grade 3 | Severe: marked limitation in activity, some assistance usually required, medical intervention/therapy required |
| Grade 4 | Potentially Life-threatening: required assessment in A&E or hospitalisation |

Table S2. Baseline characteristics of the primary efficacy cohort, by country (study).

|  | **UK (COV002)** | | | | **Brazil (COV003)** | | | |
| --- | --- | --- | --- | --- | --- | --- | --- | --- |
|  | **ChAdOx1 nCoV-19** | | **Control** | | **ChAdOx1 nCoV-19** | | **Control** | |
|  | **Female** | **Male** | **Female** | **Male** | **Female** | **Male** | **Female** | **Male** |
| N | 1596 | 1270 | 1650 | 1261 | 2647 | 2106 | 2500 | 2139 |
| Age (years), median [IQR] | 46.0 [35.0, 56.0] | 51.0 [37.0, 67.0] | 47.0 [36.0, 56.0] | 51.0 [37.1, 67.0] | 38.0 [29.0, 50.0] | 40.0 [30.2, 52.0] | 38.0 [29.0, 50.0] | 39.0 [30.0, 51.0] |
| Age categories: |  |  |  |  |  |  |  |  |
| *18-25 years* | 115 (7.2%) | 54 (4.3%) | 105 (6.4%) | 57 (4.5%) | 327 (12.4%) | 257 (12.2%) | 326 (13.0%) | 232 (10.8%) |
| *26-35 years* | 305 (19.1%) | 238 (18.7%) | 304 (18.4%) | 226 (17.9%) | 823 (31.1%) | 564 (26.8%) | 746 (29.8%) | 607 (28.4%) |
| *36-45 years* | 361 (22.6%) | 247 (19.4%) | 373 (22.6%) | 244 (19.3%) | 617 (23.3%) | 529 (25.1%) | 603 (24.1%) | 565 (26.4%) |
| *46-55 years* | 406 (25.4%) | 198 (15.6%) | 440 (26.7%) | 208 (16.5%) | 406 (15.3%) | 315 (15.0%) | 372 (14.9%) | 327 (15.3%) |
| *56-65 years* | 191 (12.0%) | 196 (15.4%) | 199 (12.1%) | 184 (14.6%) | 331 (12.5%) | 276 (13.1%) | 333 (13.3%) | 262 (12.2%) |
| *≥66 years* | 218 (13.7%) | 337 (26.5%) | 229 (13.9%) | 342 (27.1%) | 143 (5.4%) | 165 (7.8%) | 120 (4.8%) | 146 (6.8%) |
| BMI, median [IQR] | 25.1 [22.4, 29.0] | 25.9 [23.7, 28.4] | 25.3 [22.6, 29.4] | 25.8 [23.4, 28.5] | 24.8 [22.1, 28.3] | 27.0 [24.6, 30.1] | 25.0 [22.4, 28.7] | 27.0 [24.5, 30.0] |
| Health/social care worker | 1020 (63.9%) | 503 (39.6%) | 1067 (64.7%) | 524 (41.6%) | 1917 (72.4%) | 1170 (55.6%) | 1799 (72.0%) | 1162 (54.3%) |
| Ethnicity: |  |  |  |  |  |  |  |  |
| *White* | 1488 (93.2%) | 1140 (89.8%) | 1552 (94.1%) | 1133 (89.8%) | 1837 (69.4%) | 1453 (69.0%) | 1791 (71.6%) | 1448 (67.7%) |
| *Black* | 6 (0.4%) | 12 (0.9%) | 3 (0.2%) | 12 (1.0%) | 226 (8.5%) | 183 (8.7%) | 220 (8.8%) | 188 (8.8%) |
| *Asian* | 58 (3.6%) | 86 (6.8%) | 65 (3.9%) | 81 (6.4%) | 71 (2.7%) | 53 (2.5%) | 58 (2.3%) | 40 (1.9%) |
| *Mixed* | 35 (2.2%) | 17 (1.3%) | 23 (1.4%) | 20 (1.6%) | 506 (19.1%) | 403 (19.1%) | 413 (16.5%) | 457 (21.4%) |
| *Other* | 9 (0.6%) | 15 (1.2%) | 7 (0.4%) | 15 (1.2%) | 7 (0.3%) | 14 (0.7%) | 18 (0.7%) | 6 (0.3%) |
| Co-morbidities: |  |  |  |  |  |  |  |  |
| *Cardiovascular disease* | 148 (9.3%) | 261 (20.6%) | 162 (9.8%) | 259 (20.5%) | 347 (13.1%) | 449 (21.3%) | 331 (13.2%) | 448 (20.9%) |
| *Respiratory disease* | 195 (12.2%) | 148 (11.7%) | 207 (12.5%) | 155 (12.3%) | 278 (10.5%) | 209 (9.9%) | 250 (10.0%) | 198 (9.3%) |
| *Diabetes* | 30 (1.9%) | 49 (3.9%) | 32 (1.9%) | 41 (3.3%) | 91 (3.4%) | 140 (6.6%) | 86 (3.4%) | 99 (4.6%) |

| **Cohort** | **Health or social care worker** | **Female**  N (%, 95% CI) | **Male**  N (%, 95% CI) | **P-value** |
| --- | --- | --- | --- | --- |
| Pooled | Yes | 5704 (64.1%, 63.1%-65.1%) | 3195 (35.9%, 34.9%-36.9%) | <0.0001 |
|  | No | 1979 | 2591 |  |
| UK (COV002) | Yes | 2070 (67.5%, 65.8%-69.1%) | 997 (32.5%, 30.9%-34.2%) | <0.0001 |
|  | No | 729 | 855 |  |
| Brazil (COV003) | Yes | 3634 (62.3%, 61.1%-63.6%) | 2198 (37.7%, 36.4%-38.9%) | <0.0001 |
|  | No | 1250 | 1736 |  |

Table S3. Distribution of sex and health or social care worker status in participants aged 18-65 years*, by study.

*Considered a generalisable working age range.
95% CIs are binomial confidence intervals. P_-values from chi-squared tests.

**Table S4. Average daily COVID-19 patient contacts in health or social care workers aged 18-65 years, by sex.**

| **Cohort** | **Average daily COVID-19 patient contacts** | **Female**  N (%, 95% CI) | **Male**  N (%, 95% CI) | **P-value** |
| --- | --- | --- | --- | --- |
| Pooled | <1 | 3387 (59.4%, 58.1%-60.7%) | 1843 (57.7%, 55.9%-59.4%) | 0.1191 |
|  | ≥1 | 2317 (40.6%, 39.3%-41.9%) | 1352 (42.3%, 40.6%-44.1%) |  |
| UK (COV002) | <1 | 1572 (75.9%, 74.0%-77.8%) | 689 (69.1%, 66.1%-72.0%) | 0.0001 |
|  | ≥1 | 498 (24.1%, 22.2%-26.0%) | 308 (30.9%, 28.0%-33.9%) |  |
| Brazil (COV003) | <1 | 1815 (49.9%, 48.3%-51.6%) | 1154 (52.5%, 50.4%-54.6%) | 0.0583 |
|  | ≥1 | 1819 (50.1%, 48.4%-51.7%) | 1044 (47.5%, 45.4%-49.6%) |  |

95% CIs are binomial confidence intervals. P-values from chi-squared tests.

**Table S5. Severity by WHO severity score of primary symptomatic COVID-19 disease, by sex.**

| **WHO severity score** | **ChAdOx1 nCoV-19** | | **Control** | |
| --- | --- | --- | --- | --- |
|  | **Female (n=109)** | **Male (n=75)** | **Female (n=261)** | **Male (n=218)** |
| 1 (asymptomatic) | 0 (0.0%) | 0 (0.0%) | 0 (0.0%) | 0 (0.0%) |
| 2 | 47 (43.1%) | 40 (53.3%) | 81 (31.0%) | 72 (33.0%) |
| 3 | 61 (56.0%) | 35 (46.7%) | 174 (66.7%) | 132 (60.6%) |
| 4 (hospitalized) | 0 (0.0%) | 0 (0.0%) | 2 (0.8%) | 2 (0.9%) |
| 5 | 1 (0.9%) | 0 (0.0%) | 1 (0.4%) | 7 (3.2%) |
| 6 (severe) | 0 (0.0%) | 0 (0.0%) | 3 (1.1%) | 3 (1.4%) |
| 7 | 0 (0.0%) | 0 (0.0%) | 0 (0.0%) | 0 (0.0%) |
| 8 | 0 (0.0%) | 0 (0.0%) | 0 (0.0%) | 0 (0.0%) |
| 9 | 0 (0.0%) | 0 (0.0%) | 0 (0.0%) | 0 (0.0%) |
| 10 (death) | 0 (0.0%) | 0 (0.0%) | 0 (0.0%) | 2 (0.9%) |

Association between severity of disease and sex was insignificant in both ChAdOx1 nCov-19 and control groups (p=0.1299 and 0.1453, respectively; p-values from Cochran Armitage test for trend).

Table S6. Baseline characteristics of the reactogenicity and immunogenicity cohorts, by sex.

|  |  | **Reactogenicity cohorts** | |  | **Immunogenicity cohorts** | | | |
| --- | --- | --- | --- | --- | --- | --- | --- | --- |
|  | **First dose** | | **Second dose** | | **Anti-SARS-CoV-2 IgG** | | **Antibody neutralisation** | |
|  | **Female** | **Male** | **Female** | **Male** | **Female** | **Male** | **Female** | **Male** |
| N | 414 | 374 | 357 | 319 | 869 | 674 | 419 | 329 |
| Age (years), median [IQR] | 48.0 [35.0, 60.0] | 60.0 [42.0, 72.0] | 50.0 [38.0, 63.0] | 62.0 [44.0, 72.0] | 43.0 [31.0, 54.0] | 42.0 [32.0, 57.0] | 44.0 [32.5, 55.0] | 44.0 [34.0, 58.0] |
| Age categories: |  |  |  |  |  |  |  |  |
| *18-25 years* | 30 (7.2%) | 14 (3.7%) | 20 (5.6%) | 10 (3.1%) | 69 (7.9%) | 45 (6.7%) | 32 (7.6%) | 17 (5.2%) |
| *26-35 years* | 80 (19.3%) | 53 (14.2%) | 62 (17.4%) | 41 (12.9%) | 226 (26.0%) | 183 (27.2%) | 98 (23.4%) | 76 (23.1%) |
| *36-45 years* | 66 (15.9%) | 46 (12.3%) | 57 (16.0%) | 35 (11.0%) | 199 (22.9%) | 148 (22.0%) | 89 (21.2%) | 81 (24.6%) |
| *46-55 years* | 105 (25.4%) | 44 (11.8%) | 92 (25.8%) | 36 (11.3%) | 199 (22.9%) | 113 (16.8%) | 99 (23.6%) | 56 (17.0%) |
| *56-65 years* | 45 (10.9%) | 61 (16.3%) | 42 (11.8%) | 50 (15.7%) | 117 (13.5%) | 88 (13.1%) | 71 (16.9%) | 52 (15.8%) |
| *≥66 years* | 88 (21.3%) | 156 (41.7%) | 84 (23.5%) | 147 (46.1%) | 59 (6.8%) | 97 (14.4%) | 30 (7.2%) | 47 (14.3%) |
| BMI, median [IQR] | 24.8 [22.1, 28.1] | 26.2 [24.2, 29.1] | 24.8 [22.2, 28.2] | 26.1 [24.0, 29.0] | 25.1 [22.3, 28.8] | 26.2 [24.0, 29.0] | 25.1 [22.2, 29.0] | 26.4 [24.2, 28.8] |
| Country: |  |  |  |  |  |  |  |  |
| *UK (COV002)* | 369 (89.1%) | 339 (90.6%) | 335 (93.8%) | 298 (93.4%) | 541 (62.3%) | 432 (64.1%) | 261 (62.3%) | 223 (67.8%) |
| *Brazil (COV003)* | 45 (10.9%) | 35 (9.4%) | 22 (6.2%) | 21 (6.6%) | 328 (37.7%) | 242 (35.9%) | 158 (37.7%) | 106 (32.2%) |
| Health or social care worker | 250 (60.4%) | 115 (30.7%) | 202 (56.6%) | 82 (25.7%) | 678 (78.0%) | 372 (55.2%) | 333 (79.5%) | 200 (60.8%) |
| Ethnicity: |  |  |  |  |  |  |  |  |
| *White* | 393 (94.9%) | 355 (94.9%) | 342 (95.8%) | 304 (95.3%) | 707 (81.4%) | 535 (79.4%) | 344 (82.1%) | 263 (79.9%) |
| *Black* | 1 (0.2%) | 1 (0.3%) | 0 (0.0%) | 1 (0.3%) | 43 (4.9%) | 36 (5.3%) | 20 (4.8%) | 16 (4.9%) |
| *Asian* | 10 (2.4%) | 11 (2.9%) | 10 (2.8%) | 11 (3.4%) | 28 (3.2%) | 36 (5.3%) | 17 (4.1%) | 23 (7.0%) |
| *Mixed* | 9 (2.2%) | 6 (1.6%) | 4 (1.1%) | 2 (0.6%) | 86 (9.9%) | 60 (8.9%) | 37 (8.8%) | 24 (7.3%) |
| *Other* | 1 (0.2%) | 1 (0.3%) | 1 (0.3%) | 1 (0.3%) | 5 (0.6%) | 7 (1.0%) | 1 (0.2%) | 3 (0.9%) |
| *Missing* | 0 (0.0%) | 0 (0.0%) | 0 (0.0%) | 0 (0.0%) | 0 (0.0%) | 0 (0.0%) | 0 (0.0%) | 0 (0.0%) |
| Co-morbidities: |  |  |  |  |  |  |  |  |
| *Cardiovascular disease* | 46 (11.1%) | 99 (26.5%) | 43 (12.0%) | 86 (27.0%) | 99 (11.4%) | 123 (18.2%) | 49 (11.7%) | 65 (19.8%) |
| *Respiratory disease* | 48 (11.6%) | 42 (11.2%) | 44 (12.3%) | 34 (10.7%) | 119 (13.7%) | 61 (9.1%) | 54 (12.9%) | 32 (9.7%) |
| *Diabetes* | 5 (1.2%) | 21 (5.6%) | 5 (1.4%) | 19 (6.0%) | 18 (2.1%) | 32 (4.7%) | 12 (2.9%) | 18 (5.5%) |

Table S7. Immune responses at 28 days after a second dose, by sex.

| **Assay** | **Sex** | **n** | **Median [IQR]** | **GMT (95% CI)** | **GMR (95% CI)** | **p-value** | **Adjusted* GMR (95% CI)** | **P-value*** |
| --- | --- | --- | --- | --- | --- | --- | --- | --- |
| SARS-CoV-2 anti-spike IgG (AU/mL) | Male | 674 | 25484 [14623, 48296] | 24687 (22860, 26661) | ref | 0.0035 | ref | 0.0054 |
|  | Female | 869 | 30351 [16160, 47970] | 28469 (26890, 30141) | 1.15 (1.05, 1.27) |  | 1.14 (1.04, 1.26) |  |
| Neutralising antibody (IC_50_) | Male | 329 | 137 [64, 296] | 147 (132, 163) | ref | 0.4223 | ref | 0.2795 |
|  | Female | 419 | 152 [73, 289] | 155 (142, 170) | 1.06 (0.92, 1.22) |  | 1.08 (0.94, 1.24) |  |

*linear model of log-transformed antibody values, adjusted for country (UK/Brazil), age, healthcare worker, ethnicity, as well as the interaction between age and healthcare worker status. AU/mL: Arbitrary Units per millilitre; IC_50_: concentration achieving 50% inhibition of viral replication.

Data also shown in Figure 2.

**Table S8. Anti-spike antibody isotypes, subclasses and function in males and females at day 28 after second dose of ChAdOx1 nCoV-19*.**

| Assay | Sex | N samples | Minimum | Q1 | Median | Q3 | Maximum |
| --- | --- | --- | --- | --- | --- | --- | --- |
| ADCD | Male | 57 | 10.0 | 25.5 | 49.8 | 78.8 | 303.0 |
|  | Female | 67 | 10.0 | 29.5 | 53.9 | 80.4 | 323.0 |
| ADMP | Male | 63 | 0.2 | 0.3 | 0.6 | 1.0 | 1.7 |
|  | Female | 70 | 0.2 | 0.5 | 0.8 | 1.1 | 1.7 |
| ADNKA | Male | 52 | 3.5 | 3.5 | 6.4 | 11.1 | 24.5 |
|  | Female | 56 | 3.5 | 5.1 | 12.7 | 16.3 | 26.2 |
| ADNP | Male | 55 | 0.0 | 0.4 | 0.5 | 0.9 | 1.2 |
|  | Female | 63 | 0.1 | 0.4 | 0.6 | 0.8 | 1.7 |
| IgA | Male | 102 | 12.0 | 14.1 | 20.9 | 31.2 | 579.1 |
|  | Female | 112 | 12.0 | 12.0 | 19.4 | 34.0 | 2580.3 |
| IgG1 | Male | 102 | 18.0 | 18.0 | 74.7 | 829.8 | 6237.7 |
|  | Female | 112 | 18.0 | 18.0 | 34.9 | 937.8 | 10485.1 |
| IgG2 | Male | 62 | 0.2 | 0.2 | 0.2 | 0.2 | 2.6 |
|  | Female | 75 | 0.2 | 0.2 | 0.2 | 0.3 | 1.4 |
| IgG3 | Male | 102 | 8.0 | 31.8 | 74.1 | 142.5 | 1878.3 |
|  | Female | 112 | 8.0 | 35.8 | 67.7 | 143.6 | 2477.9 |
| IgG4 | Male | 62 | 0.2 | 0.2 | 0.2 | 0.2 | 0.6 |
|  | Female | 75 | 0.2 | 0.2 | 0.2 | 0.2 | 0.5 |
| IgM | Male | 102 | 12.0 | 14.7 | 22.1 | 35.4 | 186.2 |
|  | Female | 112 | 12.0 | 17.4 | 24.8 | 44.8 | 329.3 |

*Data also shown in Figure 3

ADCD: antibody dependent complement deposition; ADMP: antibody dependent monocyte phagocytosis; ADNKA: antibody dependent natural killer cell activation; ADNP: antibody dependent neutrophil phagocytosis. Comparisons of normalized data between males and females by Wilcoxon rank sum tests: all Bonferroni-adjusted p values all >0.05.

Table S9. Solicited systemic vaccine reactions after a first dose of ChAdOx1 nCoV-19 in men and women by age.

| **Severity*** | **Sex** | **18-25 years** | **26-35 years** | **36-45 years** | **46-55 years** | **56-65 years** | **≥66 years** |
| --- | --- | --- | --- | --- | --- | --- | --- |
| None | All | 6/44 (14%, 5%-27%) | 22/133 (17%, 11%-24%) | 23/112 (21%, 13%-29%) | 26/149 (17%, 12%-25%) | 31/106 (29%, 21%-39%) | 98/244 (40%, 34%-47%) |
|  | Male | 2/14 (14%, 2%-43%) | 12/53 (23%, 12%-36%) | 15/46 (33%, 20%-48%) | 9/44 (20%, 10%-35%) | 21/61 (34%, 23%-48%) | 71/156 (46%, 38%-54%) |
|  | Female | 4/30 (13%, 4%-31%) | 10/80 (12%, 6%-22%) | 8/66 (12%, 5%-22%) | 17/105 (16%, 10%-25%) | 10/45 (22%, 11%-37%) | 27/88 (31%, 21%-41%) |
| Any | All | 38/44 (86%, 73%-95%) | 111/133 (83%, 76%-89%) | 89/112 (79%, 71%-87%) | 123/149 (83%, 75%-88%) | 75/106 (71%, 61%-79%) | 146/244 (60%, 53%-66%) |
|  | Male | 12/14 (86%, 57%-98%) | 41/53 (77%, 64%-88%) | 31/46 (67%, 52%-80%) | 35/44 (80%, 65%-90%) | 40/61 (66%, 52%-77%) | 85/156 (54%, 46%-62%) |
|  | Female | 26/30 (87%, 69%-96%) | 70/80 (88%, 78%-94%) | 58/66 (88%, 78%-95%) | 88/105 (84%, 75%-90%) | 35/45 (78%, 63%-89%) | 61/88 (69%, 59%-79%) |
| Mild | All | 13/44 (30%, 17%-45%) | 52/133 (39%, 31%-48%) | 56/112 (50%, 40%-60%) | 81/149 (54%, 46%-63%) | 57/106 (54%, 44%-64%) | 112/244 (46%, 40%-52%) |
|  | Male | 4/14 (29%, 8%-58%) | 22/53 (42%, 28%-56%) | 16/46 (35%, 21%-50%) | 23/44 (52%, 37%-68%) | 32/61 (52%, 39%-65%) | 69/156 (44%, 36%-52%) |
|  | Female | 9/30 (30%, 15%-49%) | 30/80 (38%, 27%-49%) | 40/66 (61%, 48%-72%) | 58/105 (55%, 45%-65%) | 25/45 (56%, 40%-70%) | 43/88 (49%, 38%-60%) |
| Moderate | All | 18/44 (41%, 26%-57%) | 41/133 (31%, 23%-39%) | 32/112 (29%, 20%-38%) | 39/149 (26%, 19%-34%) | 17/106 (16%, 10%-24%) | 33/244 (14%, 9%-18%) |
|  | Male | 5/14 (36%, 13%-65%) | 13/53 (25%, 14%-38%) | 14/46 (30%, 18%-46%) | 12/44 (27%, 15%-43%) | 7/61 (11%, 5%-22%) | 15/156 (10%, 5%-15%) |
|  | Female | 13/30 (43%, 25%-63%) | 28/80 (35%, 25%-46%) | 18/66 (27%, 17%-40%) | 27/105 (26%, 18%-35%) | 10/45 (22%, 11%-37%) | 18/88 (20%, 13%-30%) |
| Severe | All | 7/44 (16%, 7%-30%) | 18/133 (14%, 8%-21%) | 1/112 (1%, 0%-5%) | 3/149 (2%, 0%-6%) | 1/106 (1%, 0%-5%) | 1/244 (0%, 0%-2%) |
|  | Male | 3/14 (21%, 5%-51%) | 6/53 (11%, 4%-23%) | 1/46 (2%, 0%-12%) | 0/44 (0%, 0%-8%) | 1/61 (2%, 0%-9%) | 1/156 (1%, 0%-4%) |
|  | Female | 4/30 (13%, 4%-31%) | 12/80 (15%, 8%-25%) | 0/66 (0%, 0%-5%) | 3/105 (3%, 1%-8%) | 0/45 (0%, 0%-8%) | 0/88 (0%, 0%-4%) |
| Moderate or severe | All | 25/44 (57%, 41%-72%) | 59/133 (44%, 36%-53%) | 33/112 (29%, 21%-39%) | 42/149 (28%, 21%-36%) | 18/106 (17%, 10%-26%) | 34/244 (14%, 10%-19%) |
|  | Male | 8/14 (57%, 29%-82%) | 19/53 (36%, 23%-50%) | 15/46 (33%, 20%-48%) | 12/44 (27%, 15%-43%) | 8/61 (13%, 6%-24%) | 16/156 (10%, 6%-16%) |
|  | Female | 17/30 (57%, 37%-75%) | 40/80 (50%, 39%-61%) | 18/66 (27%, 17%-40%) | 30/105 (29%, 20%-38%) | 10/45 (22%, 11%-37%) | 18/88 (20%, 13%-30%) |

*The severity gradings presented in this table are the maximum severity recorded per participant for any of the solicited systemic adverse reactions over the first 0-7 days following a first dose of ChAdOx1 nCoV-19. Hospitalisation was an additional category for solicited adverse event severity; however no reported solicited AEs resulted in hospitalisation.

Table S10. Solicited local reactions after a first dose of ChAdOx1 nCoV-19 in men and women by age.

| **Severity*** | **Sex** | **18-25 years** | **26-35 years** | **36-45 years** | **46-55 years** | **56-65 years** | **≥66 years** |
| --- | --- | --- | --- | --- | --- | --- | --- |
| None | All | 4/44 (9%, 3%-22%) | 15/133 (11%, 6%-18%) | 13/112 (12%, 6%-19%) | 26/149 (17%, 12%-25%) | 23/106 (22%, 14%-31%) | 103/244 (42%, 36%-49%) |
|  | Male | 1/14 (7%, 0%-34%) | 5/53 (9%, 3%-21%) | 6/46 (13%, 5%-26%) | 9/44 (20%, 10%-35%) | 13/61 (21%, 12%-34%) | 65/156 (42%, 34%-50%) |
|  | Female | 3/30 (10%, 2%-27%) | 10/80 (12%, 6%-22%) | 7/66 (11%, 4%-21%) | 17/105 (16%, 10%-25%) | 10/45 (22%, 11%-37%) | 38/88 (43%, 33%-54%) |
| Any | All | 40/44 (91%, 78%-97%) | 118/133 (89%, 82%-94%) | 99/112 (88%, 81%-94%) | 123/149 (83%, 75%-88%) | 83/106 (78%, 69%-86%) | 141/244 (58%, 51%-64%) |
|  | Male | 13/14 (93%, 66%-100%) | 48/53 (91%, 79%-97%) | 40/46 (87%, 74%-95%) | 35/44 (80%, 65%-90%) | 48/61 (79%, 66%-88%) | 91/156 (58%, 50%-66%) |
|  | Female | 27/30 (90%, 73%-98%) | 70/80 (88%, 78%-94%) | 59/66 (89%, 79%-96%) | 88/105 (84%, 75%-90%) | 35/45 (78%, 63%-89%) | 50/88 (57%, 46%-67%) |
| Mild | All | 28/44 (64%, 48%-78%) | 90/133 (68%, 59%-76%) | 84/112 (75%, 66%-83%) | 117/149 (79%, 71%-85%) | 81/106 (76%, 67%-84%) | 138/244 (57%, 50%-63%) |
|  | Male | 11/14 (79%, 49%-95%) | 37/53 (70%, 56%-82%) | 34/46 (74%, 59%-86%) | 32/44 (73%, 57%-85%) | 47/61 (77%, 65%-87%) | 91/156 (58%, 50%-66%) |
|  | Female | 17/30 (57%, 37%-75%) | 53/80 (66%, 55%-76%) | 50/66 (76%, 64%-85%) | 85/105 (81%, 72%-88%) | 34/45 (76%, 60%-87%) | 47/88 (53%, 42%-64%) |
| Moderate | All | 11/44 (25%, 13%-40%) | 27/133 (20%, 14%-28%) | 14/112 (12%, 7%-20%) | 6/149 (4%, 1%-9%) | 2/106 (2%, 0%-7%) | 3/244 (1%, 0%-4%) |
|  | Male | 2/14 (14%, 2%-43%) | 11/53 (21%, 11%-34%) | 5/46 (11%, 4%-24%) | 3/44 (7%, 1%-19%) | 1/61 (2%, 0%-9%) | 0/156 (0%, 0%-2%) |
|  | Female | 9/30 (30%, 15%-49%) | 16/80 (20%, 12%-30%) | 9/66 (14%, 6%-24%) | 3/105 (3%, 1%-8%) | 1/45 (2%, 0%-12%) | 3/88 (3%, 1%-10%) |
| Severe | All | 1/44 (2%, 0%-12%) | 1/133 (1%, 0%-4%) | 1/112 (1%, 0%-5%) | 0/149 (0%, 0%-2%) | 0/106 (0%, 0%-3%) | 0/244 (0%, 0%-2%) |
|  | Male | 0/14 (0%, 0%-23%) | 0/53 (0%, 0%-7%) | 1/46 (2%, 0%-12%) | 0/44 (0%, 0%-8%) | 0/61 (0%, 0%-6%) | 0/156 (0%, 0%-2%) |
|  | Female | 1/30 (3%, 0%-17%) | 1/80 (1%, 0%-7%) | 0/66 (0%, 0%-5%) | 0/105 (0%, 0%-3%) | 0/45 (0%, 0%-8%) | 0/88 (0%, 0%-4%) |
| Moderate or severe | All | 12/44 (27%, 15%-43%) | 28/133 (21%, 14%-29%) | 15/112 (13%, 8%-21%) | 6/149 (4%, 1%-9%) | 2/106 (2%, 0%-7%) | 3/244 (1%, 0%-4%) |
|  | Male | 2/14 (14%, 2%-43%) | 11/53 (21%, 11%-34%) | 6/46 (13%, 5%-26%) | 3/44 (7%, 1%-19%) | 1/61 (2%, 0%-9%) | 0/156 (0%, 0%-2%) |
|  | Female | 10/30 (33%, 17%-53%) | 17/80 (21%, 13%-32%) | 9/66 (14%, 6%-24%) | 3/105 (3%, 1%-8%) | 1/45 (2%, 0%-12%) | 3/88 (3%, 1%-10%) |

*The severity gradings presented in this table are the maximum severity recorded per participant for any of the solicited local adverse reactions over the first 0-7 days following a first dose of ChAdOx1 nCoV-19. Hospitalisation was an additional category for solicited adverse event severity; however no reported solicited AEs resulted in hospitalisation.

Table S11. Solicited systemic reactions after a second dose of ChAdOx1 nCoV-19 in men and women by age.

| **Severity*** | **Sex** | **18-25 years** | **26-35 years** | **36-45 years** | **46-55 years** | **56-65 years** | **≥66 years** |
| --- | --- | --- | --- | --- | --- | --- | --- |
| None | All | 10/30 (33%, 17%-53%) | 38/103 (37%, 28%-47%) | 27/92 (29%, 20%-40%) | 45/128 (35%, 27%-44%) | 38/92 (41%, 31%-52%) | 133/231 (58%, 51%-64%) |
|  | Male | 3/10 (30%, 7%-65%) | 16/41 (39%, 24%-55%) | 11/35 (31%, 17%-49%) | 12/36 (33%, 19%-51%) | 22/50 (44%, 30%-59%) | 91/147 (62%, 54%-70%) |
|  | Female | 7/20 (35%, 15%-59%) | 22/62 (35%, 24%-49%) | 16/57 (28%, 17%-42%) | 33/92 (36%, 26%-47%) | 16/42 (38%, 24%-54%) | 42/84 (50%, 39%-61%) |
| Any | All | 20/30 (67%, 47%-83%) | 65/103 (63%, 53%-72%) | 65/92 (71%, 60%-80%) | 83/128 (65%, 56%-73%) | 54/92 (59%, 48%-69%) | 98/231 (42%, 36%-49%) |
|  | Male | 7/10 (70%, 35%-93%) | 25/41 (61%, 45%-76%) | 24/35 (69%, 51%-83%) | 24/36 (67%, 49%-81%) | 28/50 (56%, 41%-70%) | 56/147 (38%, 30%-46%) |
|  | Female | 13/20 (65%, 41%-85%) | 40/62 (65%, 51%-76%) | 41/57 (72%, 58%-83%) | 59/92 (64%, 53%-74%) | 26/42 (62%, 46%-76%) | 42/84 (50%, 39%-61%) |
| Mild | All | 14/30 (47%, 28%-66%) | 40/103 (39%, 29%-49%) | 47/92 (51%, 40%-62%) | 66/128 (52%, 43%-60%) | 46/92 (50%, 39%-61%) | 82/231 (35%, 29%-42%) |
|  | Male | 6/10 (60%, 26%-88%) | 17/41 (41%, 26%-58%) | 18/35 (51%, 34%-69%) | 20/36 (56%, 38%-72%) | 23/50 (46%, 32%-61%) | 45/147 (31%, 23%-39%) |
|  | Female | 8/20 (40%, 19%-64%) | 23/62 (37%, 25%-50%) | 29/57 (51%, 37%-64%) | 46/92 (50%, 39%-61%) | 23/42 (55%, 39%-70%) | 37/84 (44%, 33%-55%) |
| Moderate | All | 4/30 (13%, 4%-31%) | 25/103 (24%, 16%-34%) | 17/92 (18%, 11%-28%) | 16/128 (12%, 7%-20%) | 5/92 (5%, 2%-12%) | 15/231 (6%, 4%-10%) |
|  | Male | 1/10 (10%, 0%-45%) | 8/41 (20%, 9%-35%) | 6/35 (17%, 7%-34%) | 4/36 (11%, 3%-26%) | 4/50 (8%, 2%-19%) | 10/147 (7%, 3%-12%) |
|  | Female | 3/20 (15%, 3%-38%) | 17/62 (27%, 17%-40%) | 11/57 (19%, 10%-32%) | 12/92 (13%, 7%-22%) | 1/42 (2%, 0%-13%) | 5/84 (6%, 2%-13%) |
| Severe | All | 2/30 (7%, 1%-22%) | 0/103 (0%, 0%-4%) | 1/92 (1%, 0%-6%) | 1/128 (1%, 0%-4%) | 3/92 (3%, 1%-9%) | 1/231 (0%, 0%-2%) |
|  | Male | 0/10 (0%, 0%-31%) | 0/41 (0%, 0%-9%) | 0/35 (0%, 0%-10%) | 0/36 (0%, 0%-10%) | 1/50 (2%, 0%-11%) | 1/147 (1%, 0%-4%) |
|  | Female | 2/20 (10%, 1%-32%) | 0/62 (0%, 0%-6%) | 1/57 (2%, 0%-9%) | 1/92 (1%, 0%-6%) | 2/42 (5%, 1%-16%) | 0/84 (0%, 0%-4%) |
| Moderate or severe | All | 6/30 (20%, 8%-39%) | 25/103 (24%, 16%-34%) | 18/92 (20%, 12%-29%) | 17/128 (13%, 8%-20%) | 8/92 (9%, 4%-16%) | 16/231 (7%, 4%-11%) |
|  | Male | 1/10 (10%, 0%-45%) | 8/41 (20%, 9%-35%) | 6/35 (17%, 7%-34%) | 4/36 (11%, 3%-26%) | 5/50 (10%, 3%-22%) | 11/147 (7%, 4%-13%) |
|  | Female | 5/20 (25%, 9%-49%) | 17/62 (27%, 17%-40%) | 12/57 (21%, 11%-34%) | 13/92 (14%, 8%-23%) | 3/42 (7%, 1%-19%) | 5/84 (6%, 2%-13%) |

*The severity gradings presented in this table are the maximum severity recorded per participant for any of the solicited systemic adverse reactions over the first 0-7 days following a second dose of ChAdOx1 nCoV-19. Hospitalisation was an additional category for solicited adverse event severity; however no reported solicited AEs resulted in hospitalisation.

Table S12. Solicited local reactions after a second dose of ChAdOx1 nCoV-19 in men and women by age.

| **Severity*** | **Sex** | **18-25 years** | **26-35 years** | **36-45 years** | **46-55 years** | **56-65 years** | **≥66 years** |
| --- | --- | --- | --- | --- | --- | --- | --- |
| None | All | 11/30 (37%, 20%-56%) | 30/103 (29%, 21%-39%) | 21/92 (23%, 15%-33%) | 31/128 (24%, 17%-33%) | 43/92 (47%, 36%-57%) | 138/231 (60%, 53%-66%) |
|  | Male | 7/10 (70%, 35%-93%) | 16/41 (39%, 24%-55%) | 10/35 (29%, 15%-46%) | 11/36 (31%, 16%-48%) | 26/50 (52%, 37%-66%) | 93/147 (63%, 55%-71%) |
|  | Female | 4/20 (20%, 6%-44%) | 14/62 (23%, 13%-35%) | 11/57 (19%, 10%-32%) | 20/92 (22%, 14%-32%) | 17/42 (40%, 26%-57%) | 45/84 (54%, 42%-65%) |
| Any | All | 19/30 (63%, 44%-80%) | 73/103 (71%, 61%-79%) | 71/92 (77%, 67%-85%) | 97/128 (76%, 67%-83%) | 49/92 (53%, 43%-64%) | 93/231 (40%, 34%-47%) |
|  | Male | 3/10 (30%, 7%-65%) | 25/41 (61%, 45%-76%) | 25/35 (71%, 54%-85%) | 25/36 (69%, 52%-84%) | 24/50 (48%, 34%-63%) | 54/147 (37%, 29%-45%) |
|  | Female | 16/20 (80%, 56%-94%) | 48/62 (77%, 65%-87%) | 46/57 (81%, 68%-90%) | 72/92 (78%, 68%-86%) | 25/42 (60%, 43%-74%) | 39/84 (46%, 35%-58%) |
| Mild | All | 17/30 (57%, 37%-75%) | 68/103 (66%, 56%-75%) | 66/92 (72%, 61%-81%) | 96/128 (75%, 67%-82%) | 47/92 (51%, 40%-62%) | 90/231 (39%, 33%-46%) |
|  | Male | 3/10 (30%, 7%-65%) | 23/41 (56%, 40%-72%) | 23/35 (66%, 48%-81%) | 25/36 (69%, 52%-84%) | 23/50 (46%, 32%-61%) | 52/147 (35%, 28%-44%) |
|  | Female | 14/20 (70%, 46%-88%) | 45/62 (73%, 60%-83%) | 43/57 (75%, 62%-86%) | 71/92 (77%, 67%-85%) | 24/42 (57%, 41%-72%) | 38/84 (45%, 34%-56%) |
| Moderate | All | 2/30 (7%, 1%-22%) | 5/103 (5%, 2%-11%) | 5/92 (5%, 2%-12%) | 1/128 (1%, 0%-4%) | 2/92 (2%, 0%-8%) | 3/231 (1%, 0%-4%) |
|  | Male | 0/10 (0%, 0%-31%) | 2/41 (5%, 1%-17%) | 2/35 (6%, 1%-19%) | 0/36 (0%, 0%-10%) | 1/50 (2%, 0%-11%) | 2/147 (1%, 0%-5%) |
|  | Female | 2/20 (10%, 1%-32%) | 3/62 (5%, 1%-13%) | 3/57 (5%, 1%-15%) | 1/92 (1%, 0%-6%) | 1/42 (2%, 0%-13%) | 1/84 (1%, 0%-6%) |
| Severe | All | 0/30 (0%, 0%-12%) | 0/103 (0%, 0%-4%) | 0/92 (0%, 0%-4%) | 0/128 (0%, 0%-3%) | 0/92 (0%, 0%-4%) | 0/231 (0%, 0%-2%) |
|  | Male | 0/10 (0%, 0%-31%) | 0/41 (0%, 0%-9%) | 0/35 (0%, 0%-10%) | 0/36 (0%, 0%-10%) | 0/50 (0%, 0%-7%) | 0/147 (0%, 0%-2%) |
|  | Female | 0/20 (0%, 0%-17%) | 0/62 (0%, 0%-6%) | 0/57 (0%, 0%-6%) | 0/92 (0%, 0%-4%) | 0/42 (0%, 0%-8%) | 0/84 (0%, 0%-4%) |
| Moderate or severe | All | 2/30 (7%, 1%-22%) | 5/103 (5%, 2%-11%) | 5/92 (5%, 2%-12%) | 1/128 (1%, 0%-4%) | 2/92 (2%, 0%-8%) | 3/231 (1%, 0%-4%) |
|  | Male | 0/10 (0%, 0%-31%) | 2/41 (5%, 1%-17%) | 2/35 (6%, 1%-19%) | 0/36 (0%, 0%-10%) | 1/50 (2%, 0%-11%) | 2/147 (1%, 0%-5%) |
|  | Female | 2/20 (10%, 1%-32%) | 3/62 (5%, 1%-13%) | 3/57 (5%, 1%-15%) | 1/92 (1%, 0%-6%) | 1/42 (2%, 0%-13%) | 1/84 (1%, 0%-6%) |

*The severity gradings presented in this table are the maximum severity recorded per participant for any of the solicited local adverse reactions over the first 0-7 days following a second dose of ChAdOx1 nCoV-19. Hospitalisation was an additional category for solicited adverse event severity; however no reported solicited AEs resulted in hospitalisation.

Table S13. Solicited adverse reactions after a first dose of ChAdOx1 nCoV-19 by sex.

| **Symptom** | **Sex** | **None** | **Mild** | **Moderate** | **Severe** | **Any** |
| --- | --- | --- | --- | --- | --- | --- |
| Feverish | Male | 310 (83%, 79%-87%) | 38 (10%, 7%-14%) | 24 (6%, 4%-9%) | 2 (1%, 0%-2%) | 64 (17%, 13%-21%) |
|  | Female | 308 (74%, 70%-79%) | 59 (14%, 11%-18%) | 42 (10%, 7%-13%) | 5 (1%, 0%-3%) | 106 (26%, 21%-30%) |
| Fever | Male | 367 (99%, 97%-100%) | 3 (1%, 0%-2%) | 2 (1%, 0%-2%) | 0 (0%, 0%-1%) | 5 (1%, 0%-3%) |
|  | Female | 391 (95%, 93%-97%) | 18 (4%, 3%-7%) | 1 (0%, 0%-1%) | 1 (0%, 0%-1%) | 20 (5%, 3%-7%) |
| Chills | Male | 315 (84%, 80%-88%) | 36 (10%, 7%-13%) | 18 (5%, 3%-7%) | 5 (1%, 0%-3%) | 59 (16%, 12%-20%) |
|  | Female | 322 (78%, 73%-82%) | 50 (12%, 9%-16%) | 38 (9%, 7%-12%) | 4 (1%, 0%-2%) | 92 (22%, 18%-27%) |
| Joint pain | Male | 312 (83%, 79%-87%) | 51 (14%, 10%-18%) | 11 (3%, 1%-5%) | 0 (0%, 0%-1%) | 62 (17%, 13%-21%) |
|  | Female | 326 (79%, 74%-83%) | 65 (16%, 12%-20%) | 18 (4%, 3%-7%) | 5 (1%, 0%-3%) | 88 (21%, 17%-26%) |
| Muscle ache | Male | 252 (67%, 62%-72%) | 107 (29%, 24%-33%) | 13 (3%, 2%-6%) | 2 (1%, 0%-2%) | 122 (33%, 28%-38%) |
|  | Female | 248 (60%, 55%-65%) | 123 (30%, 25%-34%) | 41 (10%, 7%-13%) | 2 (0%, 0%-2%) | 166 (40%, 35%-45%) |
| Fatigue | Male | 208 (56%, 50%-61%) | 127 (34%, 29%-39%) | 35 (9%, 7%-13%) | 4 (1%, 0%-3%) | 166 (44%, 39%-50%) |
|  | Female | 169 (41%, 36%-46%) | 169 (41%, 36%-46%) | 74 (18%, 14%-22%) | 2 (0%, 0%-2%) | 245 (59%, 54%-64%) |
| Headache | Male | 231 (62%, 57%-67%) | 114 (30%, 26%-35%) | 29 (8%, 5%-11%) | 0 (0%, 0%-1%) | 143 (38%, 33%-43%) |
|  | Female | 189 (46%, 41%-51%) | 159 (38%, 34%-43%) | 61 (15%, 11%-19%) | 5 (1%, 0%-3%) | 225 (54%, 49%-59%) |
| Malaise | Male | 277 (74%, 69%-78%) | 68 (18%, 14%-22%) | 28 (7%, 5%-11%) | 1 (0%, 0%-1%) | 97 (26%, 22%-31%) |
|  | Female | 272 (66%, 61%-70%) | 88 (21%, 17%-26%) | 45 (11%, 8%-14%) | 9 (2%, 1%-4%) | 142 (34%, 30%-39%) |
| Nausea | Male | 332 (89%, 85%-92%) | 37 (10%, 7%-13%) | 5 (1%, 0%-3%) | 0 (0%, 0%-1%) | 42 (11%, 8%-15%) |
|  | Female | 332 (80%, 76%-84%) | 61 (15%, 11%-19%) | 20 (5%, 3%-7%) | 1 (0%, 0%-1%) | 82 (20%, 16%-24%) |
| Pain | Male | 216 (58%, 53%-63%) | 145 (39%, 34%-44%) | 13 (3%, 2%-6%) | 0 (0%, 0%-1%) | 158 (42%, 37%-47%) |
|  | Female | 235 (57%, 52%-62%) | 154 (37%, 33%-42%) | 24 (6%, 4%-9%) | 1 (0%, 0%-1%) | 179 (43%, 38%-48%) |
| Redness | Male | 371 (99%, 98%-100%) | 19 (5%, 3%-8%) | 13 (4%, 2%-6%) | 6 (2%, 1%-4%) | 3 (1%, 0%-2%) |
|  | Female | 400 (97%, 94%-98%) | 13 (4%, 2%-6%) | 14 (4%, 2%-6%) | 7 (2%, 1%-4%) | 14 (3%, 2%-6%) |
| Warmth | Male | 346 (93%, 89%-95%) | 28 (7%, 5%-11%) | 0 (0%, 0%-1%) | 0 (0%, 0%-1%) | 28 (7%, 5%-11%) |
|  | Female | 350 (85%, 81%-88%) | 62 (15%, 12%-19%) | 2 (0%, 0%-2%) | 0 (0%, 0%-1%) | 64 (15%, 12%-19%) |
| Itch | Male | 363 (97%, 95%-99%) | 10 (3%, 1%-5%) | 1 (0%, 0%-1%) | 0 (0%, 0%-1%) | 11 (3%, 1%-5%) |
|  | Female | 389 (94%, 91%-96%) | 23 (6%, 4%-8%) | 2 (0%, 0%-2%) | 0 (0%, 0%-1%) | 25 (6%, 4%-9%) |
| Swelling | Male | 370 (99%, 97%-100%) | 13 (4%, 2%-6%) | 3 (1%, 0%-2%) | 3 (1%, 0%-2%) | 4 (1%, 0%-3%) |
|  | Female | 404 (98%, 96%-99%) | 9 (2%, 1%-4%) | 9 (2%, 1%-4%) | 3 (1%, 0%-2%) | 10 (2%, 1%-4%) |
| Induration | Male | 371 (99%, 98%-100%) | 8 (2%, 1%-4%) | 4 (1%, 0%-3%) | 1 (0%, 0%-2%) | 3 (1%, 0%-2%) |
|  | Female | 405 (98%, 96%-99%) | 14 (4%, 2%-6%) | 19 (5%, 3%-8%) | 5 (1%, 0%-3%) | 9 (2%, 1%-4%) |
| Tenderness | Male | 150 (40%, 35%-45%) | 209 (56%, 51%-61%) | 14 (4%, 2%-6%) | 1 (0%, 0%-1%) | 224 (60%, 55%-65%) |
|  | Female | 115 (28%, 24%-32%) | 273 (66%, 61%-70%) | 25 (6%, 4%-9%) | 1 (0%, 0%-1%) | 299 (72%, 68%-76%) |

The severity gradings presented in this table are the maximum severity recorded per participant for any of the solicited adverse reactions over the first 0-7 days following a first dose of ChAdOx1 nCoV-19.

Table S14. Solicited adverse reactions after a second dose of ChAdOx1 nCoV-19 by sex.

| **Symptom** | **Sex** | **None** | **Mild** | **Moderate** | **Severe** | **Any** |
| --- | --- | --- | --- | --- | --- | --- |
| Feverish | Male | 297 (93%, 90%-96%) | 17 (5%, 3%-8%) | 5 (2%, 1%-4%) | 0 (0%, 0%-1%) | 22 (7%, 4%-10%) |
|  | Female | 316 (89%, 85%-92%) | 32 (9%, 6%-12%) | 7 (2%, 1%-4%) | 2 (1%, 0%-2%) | 41 (11%, 8%-15%) |
| Fever | Male | 314 (100%, 99%-100%) | 0 (0%, 0%-1%) | 0 (0%, 0%-1%) | 0 (0%, 0%-1%) | 0 (0%, 0%-1%) |
|  | Female | 346 (99%, 98%-100%) | 1 (0%, 0%-2%) | 2 (1%, 0%-2%) | 0 (0%, 0%-1%) | 3 (1%, 0%-2%) |
| Chills | Male | 300 (94%, 91%-96%) | 15 (5%, 3%-8%) | 3 (1%, 0%-3%) | 1 (0%, 0%-2%) | 19 (6%, 4%-9%) |
|  | Female | 337 (94%, 91%-97%) | 17 (5%, 3%-8%) | 3 (1%, 0%-2%) | 0 (0%, 0%-1%) | 20 (6%, 3%-9%) |
| Joint pain | Male | 281 (88%, 84%-91%) | 29 (9%, 6%-13%) | 9 (3%, 1%-5%) | 0 (0%, 0%-1%) | 38 (12%, 9%-16%) |
|  | Female | 317 (89%, 85%-92%) | 36 (10%, 7%-14%) | 4 (1%, 0%-3%) | 0 (0%, 0%-1%) | 40 (11%, 8%-15%) |
| Muscle ache | Male | 253 (79%, 74%-84%) | 54 (17%, 13%-22%) | 12 (4%, 2%-6%) | 0 (0%, 0%-1%) | 66 (21%, 16%-26%) |
|  | Female | 277 (78%, 73%-82%) | 71 (20%, 16%-24%) | 9 (3%, 1%-5%) | 0 (0%, 0%-1%) | 80 (22%, 18%-27%) |
| Fatigue | Male | 211 (66%, 61%-71%) | 88 (28%, 23%-33%) | 18 (6%, 3%-9%) | 2 (1%, 0%-2%) | 108 (34%, 29%-39%) |
|  | Female | 213 (60%, 54%-65%) | 114 (32%, 27%-37%) | 27 (8%, 5%-11%) | 3 (1%, 0%-2%) | 144 (40%, 35%-46%) |
| Headache | Male | 228 (71%, 66%-76%) | 76 (24%, 19%-29%) | 14 (4%, 2%-7%) | 1 (0%, 0%-2%) | 91 (29%, 24%-34%) |
|  | Female | 213 (60%, 54%-65%) | 118 (33%, 28%-38%) | 26 (7%, 5%-10%) | 0 (0%, 0%-1%) | 144 (40%, 35%-46%) |
| Malaise | Male | 261 (82%, 77%-86%) | 45 (14%, 10%-18%) | 12 (4%, 2%-6%) | 1 (0%, 0%-2%) | 58 (18%, 14%-23%) |
|  | Female | 291 (82%, 77%-85%) | 49 (14%, 10%-18%) | 15 (4%, 2%-7%) | 2 (1%, 0%-2%) | 66 (18%, 15%-23%) |
| Nausea | Male | 300 (94%, 91%-96%) | 18 (6%, 3%-9%) | 1 (0%, 0%-2%) | 0 (0%, 0%-1%) | 19 (6%, 4%-9%) |
|  | Female | 317 (89%, 85%-92%) | 36 (10%, 7%-14%) | 3 (1%, 0%-2%) | 1 (0%, 0%-2%) | 40 (11%, 8%-15%) |
| Pain | Male | 243 (76%, 71%-81%) | 73 (23%, 18%-28%) | 3 (1%, 0%-3%) | 0 (0%, 0%-1%) | 76 (24%, 19%-29%) |
|  | Female | 241 (68%, 62%-72%) | 111 (31%, 26%-36%) | 5 (1%, 0%-3%) | 0 (0%, 0%-1%) | 116 (32%, 28%-38%) |
| Redness | Male | 318 (100%, 98%-100%) | 19 (6%, 4%-9%) | 10 (3%, 2%-6%) | 5 (2%, 1%-4%) | 1 (0%, 0%-2%) |
|  | Female | 353 (99%, 97%-100%) | 14 (4%, 2%-7%) | 16 (5%, 3%-8%) | 11 (3%, 2%-6%) | 4 (1%, 0%-3%) |
| Warmth | Male | 303 (95%, 92%-97%) | 16 (5%, 3%-8%) | 0 (0%, 0%-1%) | 0 (0%, 0%-1%) | 16 (5%, 3%-8%) |
|  | Female | 305 (85%, 81%-89%) | 51 (14%, 11%-18%) | 1 (0%, 0%-2%) | 0 (0%, 0%-1%) | 52 (15%, 11%-19%) |
| Itch | Male | 313 (98%, 96%-99%) | 5 (2%, 1%-4%) | 1 (0%, 0%-2%) | 0 (0%, 0%-1%) | 6 (2%, 1%-4%) |
|  | Female | 337 (94%, 91%-97%) | 20 (6%, 3%-9%) | 0 (0%, 0%-1%) | 0 (0%, 0%-1%) | 20 (6%, 3%-9%) |
| Swelling | Male | 318 (100%, 98%-100%) | 4 (1%, 0%-3%) | 6 (2%, 1%-4%) | 3 (1%, 0%-3%) | 1 (0%, 0%-2%) |
|  | Female | 354 (99%, 98%-100%) | 19 (6%, 3%-9%) | 7 (2%, 1%-4%) | 7 (2%, 1%-4%) | 3 (1%, 0%-2%) |
| Induration | Male | 319 (100%, 99%-100%) | 6 (2%, 1%-4%) | 4 (1%, 0%-3%) | 2 (1%, 0%-2%) | 0 (0%, 0%-1%) |
|  | Female | 354 (99%, 98%-100%) | 13 (4%, 2%-6%) | 13 (4%, 2%-6%) | 4 (1%, 0%-3%) | 3 (1%, 0%-2%) |
| Tenderness | Male | 191 (60%, 54%-65%) | 125 (39%, 34%-45%) | 3 (1%, 0%-3%) | 0 (0%, 0%-1%) | 128 (40%, 35%-46%) |
|  | Female | 137 (38%, 33%-44%) | 212 (59%, 54%-65%) | 8 (2%, 1%-4%) | 0 (0%, 0%-1%) | 220 (62%, 56%-67%) |

The severity gradings presented in this table are the maximum severity recorded per participant for any of the solicited adverse reactions over the first 0-7 days following a second dose of ChAdOx1 nCoV-19.
