# Supplementary material for: An exploratory analysis of the response to ChAdOx1 nCoV-19 (AZD1222) vaccine in males and females
Source: eBioMedicine. 2022 Jun 30;81:104128. doi: 10.1016/j.ebiom.2022.104128 (PMC9242842; doi:10.1016/j.ebiom.2022.104128)
Supplement: Supplementary file 8 [file mmc8.pdf]

Global Statistical Analysis Plan

for the combined integrated analysis of studies of ChAdOx1 nCoV-19 (AZD1222) vaccine

Study	Current Version of Protocol
COV001 (UK)	COV001_Protocol_v12.0_09 Nov_2020 clean
COV002 (UK)	COV002_Protocol_v14.0_09 Nov_2020 clean
COV003 (Brazil)	COV003 ChAdOx1 nCoV-19 Trial protocol V5_20200827_clean
COV005 (South Africa)	ChAdOx1-nCoV-19_ZA_Protocol_v4 1_30Sept2020_signed

	NAME	TITLE	SIGNATURE	DATE
Written by:	Merryn Voysey	Lead Statistician		19 Nov 2020
Approved by:	Andrew Pollard	Chief Investigator		19 Nov 2020

Version:	Version Date:	Changes:
1.0	06 Nov 2020	First Draft
1.1	12 Nov 2020	Updated to append MAA SAP – version 4
1.2	19 Nov 2020	Updated to append MAA SAP – version 5

Table of Contents

1 Introduction ii

2 Integrated Summary for Marketing Authorisation Application..........1

1 Introduction

This document details the approach that will be used to assess the efficacy and safety of the University of Oxford sponsored studies of ChAdOx1 nCoV-19 vaccine (AZD1222).

The analysis detailed herein has been developed in collaboration with AstraZeneca for the purposes of the marketing authorisation application for the vaccine and does not preclude additional analyses being conducted that are beyond those required for regulatory submissions. Any additional analyses should follow the principles of the analysis detailed in the global SAP to the extent that is possible.

Statistical Analysis Plan

Study Code	D8111
Edition Number	5.0
Date	17 November 2020

Integrated Summary for Marketing Authorisation Application

**Statistical Analysis Plan**	
Study Code	D8111
Edition Number	5.0
Date	17 November 2020

Integrated Summary for Marketing Authorisation Application

Reviewed and approved by:

Dongmei Lan, MS.
Lead Statistician
Cytel

Date

Kathryn Shoemaker, MS.

Global Product Statistician
AstraZeneca

Date

TABLE OF CONTENTS

TITLE PAGE 1
SIGNATURE PAGE 2
TABLE OF CONTENTS 3
LIST OF ABBREVIATIONS 7
AMENDMENT HISTORY 9
1 INTRODUCTION 11
1.1 Nonclinical Experience with AZD1222 11
1.2 Clinical Experience with AZD1222 11
2. INTEGRATED ANALYSIS OBJECTIVES 12
2.1 Primary Objective of the Pooled Analysis 12
2.2 Secondary Objectives of the Pooled Analysis 12
3. AZD1222 PROTOCOL SUMMARIES 13
4. POOLING OF DATA 20
4.1 Planned Analyses 21
5. ANALYSIS POPULATIONS 23
6. DOSING REGIMENS AND TREATMENT GROUPS 25
7. OVERVIEW OF ANALYSIS CONVENTIONS 26
7.1 Data Presentation 26
7.2 Definitions of Subgroups 27
7.3 Definition of Baseline 27
7.4 Handling of Missing Data 27
7.5 Reference Start Date and Study Day 28
7.6 Windowing Conventions 28
8. SUBJECT ENROLLMENT AND DISPOSITION 30
8.1 Subject Identification 30
8.2 Subject Disposition 30
8.3 Demographics and Baseline Characteristics 30
8.4 Exposure 31
9. EFFICACY EVALUATIONS 31
9.1 Efficacy Assessments 31
9.1.1 Primary Efficacy Endpoint 32
9.1.1.1 Time to Events Endpoints for Supplementary Analysis 33
9.1.2 Secondary Efficacy Endpoints 33

9.1.2.1 Incidence of Severe COVID-19 Occurring ≥ 15 Days Post Second Dose of Study Intervention 33
9.1.2.2 Incidence of Severe COVID-19 Occurring Post First Dose of Study Intervention 33
9.1.2.3 Incidence of Severe COVID-19 Occurring ≥ 22 Days Post First Dose of Study Intervention 34
9.1.2.4 Incidence of Asymptomatic SARS-CoV-2 Infection Occurring ≥ 15 Days Post Second Dose of Study Intervention for COV002 Only 34
9.1.2.5 Incidence of Asymptomatic SARS-CoV-2 Infection Occurring ≥ 22 Days Post First Dose of Study Intervention for COV002 Only 34
9.1.2.6 Incidence of SARS-CoV-2 Virologically-confirmed COVID-19 Occurring Post First Dose of Study Intervention 34
9.1.2.7 Incidence of SARS-CoV-2 Virologically-confirmed COVID-19 Occurring 22 Days Post First Dose of Study Intervention 35
9.1.2.8 Duration of Follow-up 35
9.1.2.9 Incidence of COVID-19 Hospital Admission Occurring ≥ 15 Days Post Second Dose of Study Intervention 35
9.1.2.10 Incidence of COVID-19 Hospital Admission Occurring Post First Dose of Study Intervention 35
9.1.2.11 Incidence of COVID-19 Hospital Admission Occurring 22 Days Post First Dose of Study Intervention 35
9.1.2.12 Incidence of COVID-19 Death Occurring ≥ 15 Days Post Second Dose of Study Intervention 36
9.1.2.13 Incidence of COVID-19 Death Associated with SARS-CoV-2 Virologically-confirmed COVID-19 Occurring Post First Dose of Study Intervention 36
9.1.2.14 Incidence of COVID-19 Death Associated with SARS-CoV-2 Virologically-confirmed COVID-19 Occurring 22 Days Post First Dose of Study Intervention 36
9.1.2.15 Incidence of COVID-19 Requiring ICU Occurring ≥ 15 Days Post Second Dose of Study Intervention 36
9.1.2.16 Incidence of COVID-19 Requiring ICU Occurring Post First Dose of Study Intervention 36
9.1.2.17 Incidence of COVID-19 Requiring ICU Occurring ≥ 22 Days Post First Dose of Study Intervention 36
9.1.2.18 Secondary Efficacy Endpoint Based on Nucleocapsid Antibody: Detection of SARS-CoV-2 Nucleocapsid Antibody Levels Over Time 36
9.2 Efficacy Analyses 36
9.2.1 Primary Efficacy Analyses 37
9.2.1.1 Pooled Analysis of Primary Efficacy Endpoint 37
9.2.1.2 Secondary Analysis of Primary Endpoint 38
9.2.1.3 Subgroup Analysis of Primary Endpoint 38
9.2.1.4 Supportive Analyses for Primary Efficacy Endpoint 38
9.2.1.5 Sensitivity Analysis of Primary Endpoint 38
9.2.2 Secondary Efficacy Analyses 38
9.2.2.1 Incidence of Asymptomatic SARS-CoV-2 Infection COV002 only 38
9.2.2.2 Duration of Follow-up since the Second Vaccination 38

9.2.2.3 Other Secondary Efficacy Endpoints 39
9.2.2.4 Secondary Efficacy Analyses for Endpoints Based on Nucleocapsid: Detection AntibodySARS-CoV-2 Nucleocapsid Related Endpoints 39
10. SAFETY ANALYSIS 39
10.1 Adverse Events 39
10.1.1 Reporting of Adverse events 39
10.1.2 Evaluation of Severity 39
10.1.3 Solicited AEs 43
10.1.4 Unsolicited AEs 43
10.1.5 Serious Adverse Events (SAEs) 44
10.1.6 Adverse Events of Special Interest 44
10.1.7 Death 48
10.2 Clinical Laboratory Results 48
10.3 Subgroup Analysis 49
11. IMMUNOGENICITY ANALYSIS 49
12. REFERENCES 51
13. APPENDICES 52

LIST OF APPENDICES

Appendix A Overview of Analyses 53
Appendix B Pooling and Severity Grading of Solicited Adverse Events: Studies COV001, COV002, COV003, and COV005 55
Appendix C Pooling and Severity Grading of Laboratory Assessed on Scheduled Visits 67

LIST OF TABLES

Table 1 Summary of Pivotal Studies to be Pooled 14
Table 2 WHO Clinical Progression Scale 21
Table 3 Populations for Analysis 23
Table 4 Actual AZD1222 Dose Levels Received by CTM and HD or LD Designation 25
Table 5 Visit Window for Immunogenicity 29
Table 6 Case Definitions for Evaluation of Efficacy 32
Table 7 Severity Rating Criteria for Physical Observations (Applies to Adults Only) for COV001, COV002 and COV003 40

Table 8 Severity Grading Criteria for Select Physical Observations (Based on DAIDS Grading Table; Version 2.1 – July 2017 for COV005)................. 40
Table 9 Adverse Events of Special Interest 45
Table A1 Overview of Evaluation of Efficacy 53
Table A2 Overview of Evaluation of Safety 54
Table A3 Overview of Evaluation of Immunogenicity... 54
Table B1 Schedule and Protocol Description of Solicited Adverse Event Reporting in Patient Diaries 56
Table B2 CSP-specified Solicited Adverse Events and Patient Diary Terms for these Events by Study 58
Table B3 Fever Severity Grades for the DMID and DAIDS Grading Scales 60
Table B4 CSP-specified Severity Grading and Patient Diary Measurements for Redness, Swelling, and Induration 61
Table B5 Patient Diary Severity Grades for Pain and Warmth at Injection Site, Malaise, Nausea, and Vomiting Events in Studies COV001, COV002, and COV003 62
Table B6 Patient Diary Severity Grades for Feverishness and Chills in Studies COV001, COV002, and COV003 63
Table B7 Patient Diary Severity Grading for Tenderness, Itching, Joint Pain, Muscle Pain, Fatigue, and Headache by Study 64
Table B8 Contribution of Individual Studies to the Overall Pool for Solicited Adverse Events 65
Table C1 Laboratory Abnormality Severity Grade Criteria to be Used for Pooled COV1, COV002, and COV005 Data.. 68
Table C2 Laboratory Abnormality Severity Grade Criteria to be Used for Pooled COV001, COV002, and COV005 Data 70

LIST OF ABBREVIATIONS

Abbreviation or special term	Explanation
AE(s)	adverse event(s)
AESI	adverse event of special interest
BMI	body mass index
CBF	Clinical Biomanufacturing Facility
ChAdOx1 nCoV-19	name of AZD1222 by the University of Oxford
CI	confidence interval
COVID-19	coronavirus disease 2019
CSP	Clinical Study Protocol
CTM	clinical trial material
DAIDS	Division of AIDS
DSMB	Data and Safety Monitoring Board
ELISA	enzyme-linked immunosorbent assay
ELISpot	enzyme-linked immunospot
FDA	Food and Drug Administration
FIH	first-in-human
GMFR	geometric mean fold rise
GMT	geometric mean titre
HIV	human immunodeficiency viruses
IFN-γ	interferon-gamma
IgG	immunoglobulin G
IM	intramuscular
ITT	intent-to-treat
LD	low dose
LLOQ	lower limit of quantification
MAA	Marketing Authorisation Application
MedDRA	Medical Dictionary for Regulatory Activities
MenACWY	meningococcal Group A, C, W-135 and Y conjugate vaccine
MERS	Middle East Respiratory Syndrome
MHRA	Medicines and Healthcare Products Regulatory Agency
MNA	microneutralisation
NAb	neutralising antibody
NHP	non-human primate
NIH	national institutes of health
PRNT	plaque reduction neutralisation test

**Abbreviation or special term**	**Explanation**
RBD	receptor binding domain
RR	relative risk
RT-PCR	reverse transcriptase polymerase chain reaction
S	Spike
SAE	serious adverse event
SARS-CoV-2	severe acute respiratory syndrome-coronavirus 2
SD	standard dose
SDSD	standard dose standard dose
Std Dev	standard deviation
tPA	tissue plasminogen activator
UK	United Kingdom
VE	vaccine efficacy
vp	viral particles

AMENDMENT HISTORY

Version 5: Summary of Changes

Category*: Change refers to	Date	Description of change	Rationale
NA	04Sep2020	Not Applicable – First Version.	
Primary or secondary endpoints	02Nov2020	Primary endpoint for COV003 for IA1 was removed; endpoints related to first dose/first dose+22 days were added; the case definition for endpoints were added with WHO severity scoring.	Updated according to response from regulatory agency for planning of interim and primary analysis. Received additional information about adjudication.
Other	02Nov2020	Analysis sets were renamed/modified; overview of analysis was added in the appendix; redefined strategy for subgroups; AESI listing was replaced with updated definition in tables.	Clarify the analysis planned. Update according to new information.
Other	03Nov2020	Remove highlight in TOC; change version to 3.0 from 1.1 in title (skip version 2.0) to match with version in ANGEL. Correct the HIV group in COV002.	Clean the file.
Primary or secondary endpoints	03Nov2020	Update case definition of asymptomatic SARS-CoV-2 infection. Update the homogeneity test may be conducted and change the model of subgroup analysis.	Provide the source of information and reduce parameters in model.
Primary or secondary endpoints	11Nov2020	Added appendix for pooling of solicited events. Clarified COVID-19 infection will be virologically confirmed. Clarified subgroup analyses will be performed for all endpoints, unless specified otherwise.	Clarification of the analyses planned.
Primary or secondary endpoints	17Nov2020	Added appendix for severity grading and pooling of clinical laboratory results. Merged table 10 List of Potential Immune-mediated Medical Conditions into table 9 Adverse Events of special interest. Added clarification of control group for group 2d in COV002. Provided more information of efficacy endpoints in section 4.	Clarification of the analyses planned.

* Pre-specified categories are
Primary or secondary endpoints; Statistical analysis method for the primary or secondary endpoints; Derivation of primary or secondary endpoints; Multiple Testing Procedure; Data presentations; Other

1 INTRODUCTION

AZD1222 is a recombinant replication-defective chimpanzee adenovirus expressing the SARS-CoV-2 S surface glycoprotein driven by the human cytomegalovirus major immediate early promoter that includes intron A with a human tPA leader sequence at the N terminus.

1.1 Nonclinical Experience with AZD1222

AZD1222 has been shown to be immunogenic in BALB/c, CD-1 mice, porcine, and NHP models. Further, in a NHP SARS-CoV-2 challenge model, a single administration of AZD1222 significantly reduced viral load in bronchoalveolar lavage fluid and respiratory tract tissue of vaccinated animals as compared to vector controls (van Doremalen et al 2020). Efficacy studies in ferret and NHP models are in their final completion stage. Biodistribution studies with similar ChAd vaccines (AdCh63 ME-TRAP, AdCh63 MSP-1 and AdCh3NSmut) in mice have previously been performed and showed no evidence of replication of the virus or presence of disseminated infection after IM injections. Given these data, biodistribution studies were not performed based on advice from the MHRA. Toxicology studies have not been conducted on AZD1222. A toxicology study in mice for another ChAdOx1 vectored vaccine expressing a related betacoronavirus S surface antigen (ChAdOx1 MERS) is shown for reference. Further background information is presented in AZD1222 Investigator's Brochure, Section 4.

1.2 Clinical Experience with AZD1222

The University of Oxford is investigating the safety, immunogenicity, and efficacy of AZD1222 in 3 ongoing meningococcal vaccine-controlled clinical studies: a FIH Phase I/II Study COV001 in healthy adults 18 to 55 years of age in the UK; a Phase II/III Study COV002 in healthy adults ≥ 18 years of age (including the elderly) and children 5 to 12 years of age in the UK; and a Phase III Study COV003 in healthy adults ≥ 18 years of age in Brazil. An additional Phase I/II study using a saline placebo control, COV005, is ongoing in South Africa. For these studies, AZD1222 CTM from 3 different sources has been used: 1) The CBF at the University of Oxford; 2) Advent, Italy, and 3) Cobra Biologics. The analytical comparability assessment of AZD1222 Process 1 (CBF), Process 2 (Advent), and Process 3 (Symbiosis) for the clinical Drug Product, was conducted. Due to a potency miscalculation, participants in Groups 1, 2, 4 and 5a in Study COV002 received a lower dose of approximately 2×10^{10} vp instead of the planned dose of 5×10^{10} vp. Groups receiving this lower dose are listed as having received 5×10^{10} vp (Abs260) Advent in the study protocols.

The complete list and description of these ongoing University of Oxford-sponsored AZD1222 clinical studies is presented in Table 1.

Emerging safety and immunogenicity data from all the Oxford sponsored studies are reviewed approximately every 2 weeks by an independent DSMB. Preliminary safety and

immunogenicity data are available for Study COV001, which enrolled the first volunteer on 23 April 2020 and completed enrolment on 21 May 2020. A total of 1 077 volunteers have been enrolled, including 544 volunteers who received at least one dose of 5×10^{10} vp AZD1222 and 10 volunteers who received a second dose of 5×10^{10} vp AZD1222 (homologous prime boost) 4 weeks later. Safety data found the vaccine was generally well tolerated, with no treatmentrelated SAEs reported through 28 days post vaccination. The most common local solicited AEs were vaccination site pain and tenderness. The most common systemic solicited AEs were chills, feverishness, fever, headache, malaise, and myalgia. The majority of events were mild or moderate in severity and resolved within 1 to 7 days. Following the second dose, a general attenuation in the incidence and severity of local and systemic solicited AEs was observed, although this was based on only 10 participants.

Preliminary immunogenicity data from Study COV001 suggest that a single dose can elicit both humoral and cellular immunogenicity responses and that antibody responses are boosted after a second dose. Spike-specific T-cell responses peaked on Day 14. Anti-S IgG responses rose by Day 28 and were boosted 3-fold following a second dose.

Neutralising antibody responses against SARS-CoV-2 were detected in 32 of 35 (91%) participants after a single dose when measured in a microneutralisation assay (MNA_{80}) and in all 35 (100%) participants when measured in a plaque reduction neutralisation test ($PRNT_{50}$) by day 28. After a second dose, all participants had neutralising activity (9 of 9 in MNA_{80} at Day 42 and 10 of 10 in the Marburg virus neutralisation assay on Day 56). Neutralising antibody responses correlated strongly with antibody levels measured by ELISA (Folegatti et al 2020).

2. INTEGRATED ANALYSIS OBJECTIVES

This statistical analysis plan defines methodology and procedures in performing analyses of pooled efficacy and safety data from AZD1222 trials to determine the benefits and risks of AZD1222 as a non-replicating ChAdOx1 vector vaccine in healthy adults against COVID-19.

2.1 Primary Objective of the Pooled Analysis

To estimate the efficacy of 2 IM doses of AZD1222, with the second dose being SD, compared to control for the prevention of COVID-19 in adults ≥ 18 years of age.

2.2 Secondary Objectives of the Pooled Analysis

- To evaluate the efficacy of AZD1222 against severe COVID-19 disease.
- To assess the safety, tolerability and reactogenicity profile of AZD1222.
- To assess humoral immunogenicity of AZD1222 if data are available.
- To assess the cellular immunogenicity of AZD1222 if data are available.

3. AZD1222 PROTOCOL SUMMARIES

Table 1 Summary of Pivotal Studies to be Pooled

Element	COV001	COV002	COV003	COV005
Identifier	NCT04324606; EudraCT 2020-001072-15	NCT04400838; EudraCT 2020-001228-32	ISRCTN89951424	NCT04444674
Title	A phase I/II study to determine efficacy, safety and immunogenicity of the candidate Coronavirus Disease (COVID-19) vaccine ChAdOx1 nCoV-19 in UK healthy adult volunteers	A phase 2/3 study to determine the efficacy, safety and immunogenicity of the candidate Coronavirus Disease (COVID-19) vaccine ChAdOx1 nCoV-19	A Randomized, Controlled, Phase III Study to Determine the Safety, Efficacy, and Immunogenicity of the Non-Replicating ChAdOx1 nCoV-19 Vaccine.	An adaptive phase I/II randomized placebo-controlled trial to determine safety, immunogenicity and efficacy of non-replicating ChAdOx1 SARS-CoV-2 Vaccine in South African adults living without HIV; and safety and immunogenicity in adults living with HIV.
Region	United Kingdom	United Kingdom	Brazil	South Africa
Phase	I/II	II/III	III	I/II
Period	23Apr2020-ongoing	29 May2020-ongoing	Jun2020-ongoing	Jun2020-ongoing
Design	FIH, participant blind, randomised, controlled<br>Participant will be followed 364 days after the last dose	Participant blind, randomised, controlled<br>Participant will be followed 364 days after the last dose except groups 1b, 2b, 5d, 7b and 8b, which will be followed 364 days after the first dose	Participant blind, randomised, controlled<br>Participant will be followed 364 days after the last dose	Double blind, randomised, placebo-controlled, adaptive<br>Participant will be followed 364 days after the first dose
Primary study objective	To assess efficacy of AZD1222 against COVID-19;<br>To assess the safety of AZD1222	To assess efficacy of AZD1222 against COVID-19 in adults aged ≥18 years<br>Co-Primary: To assess the safety of AZD1222 in adults and children.	To evaluate the efficacy of AZD1222 against COVID-19 disease virologically-confirmed[c]	For group 1 and groups 2a and 2b:<br>To assess safety, tolerability and reactogenicity profile of AZD1222;<br>Co-primary objective for groups 2a and 2b:<br>To assess efficacy of AZD1222

Table 1 Summary of Pivotal Studies to be Pooled

Element	COV001	COV002	COV003	COV005
Study population	Healthy adults aged 18-55 years	Main efficacy study: Healthy adults aged ≥18 years<br>Priority given to health professionals and adults with high potential for exposure to SARS-CoV-2<br>Safety and immunogenicity substudies:<br>• Healthy children aged 5 to 12 years, inclusive[a]<br>• HIV+ adults aged 18 - 55 years	Health professionals and adults with high potential for exposure to SARS-CoV-2, aged ≥18 years	Adults aged 18-65 years, living with and without HIV
Actual treatment	AZD1222:<br>2.5×10^{10} vp<br>5×10^{10} vp<br>0.5 mL (3.5 – 6.5 × 10^{10} vp, Abs 260, corrected for PS80)<br>MenACWY: 0.5 mL	AZD1222:<br>2.2×10^{10} vp (qPCR)<br>2.5×10^{10} vp (qPCR)<br>5×10^{10} vp (Abs 260)<br>5×10^{10} vp (qPCR)<br>0.5 mL (3.5 – 6.5 × 10^{10} vp, Abs 260, corrected for PS80)<br>MenACWY: 0.5 mL	AZD1222:<br>5×10^{10} vp<br>0. 5mL (3.5 - 6.5 × 10^{10} vp)<br>MenACWY: 0.5 mL<br>0.9% saline solution: 0.5mL	AZD1222:<br>5×10^{10} vp;<br>Normal saline (0.9% NaCl)

Table 1 Summary of Pivotal Studies to be Pooled

Element	COV001	COV002	COV003	COV005
Efficacy endpoints	**Primary endpoint:** Virologically-confirmed[c] symptomatic cases of COVID-19	**Primary endpoint:** Virologically-confirmed[c] symptomatic cases of COVID-19	**Primary endpoint:** COVID-19 virologically-confirmed[c] symptomatic cases	**Primary endpoint:** Virologically-confirmed[c] COVID-19 cases occurring in participants that were COVID-19 naïve at the time of randomization and who received at least two doses of ChAdOx1 nCoV-19 or placebo. Events will be included if they occurred more than 14 days after the booster dose.

Table 1 Summary of Pivotal Studies to be Pooled

Element	COV001	COV002	COV003	COV005
Efficacy endpoints (continued)	**Secondary endpoints:**<br>a) Hospital admissions associated with COVID-19<br>b) Intensive care unit admissions associated with COVID-19<br>c) Deaths associated with COVID-19<br>d) Severe COVID-19 disease (defined according to clinical severity scales).<br>e) Seroconversion against non-Spike SARS- CoV-2 antigens	**Secondary endpoints:**<br>a) Hospital admissions associated with COVID-19<br>b) Intensive care unit admissions associated with COVID-19<br>c) Deaths associated with COVID-19<br>d) Seroconversion against non-Spike SARS-CoV-2 antigens<br>e) Severe COVID-19 disease (defined according to clinical severity scales)	**Secondary endpoints:**<br>a) Hospitalization for COVID-19 virologically-confirmed[c];<br>b) Severe COVID-19 virologically-confirmed[c];<br>c) Death associated with COVID-19;<br>d) Antibodies against SARS-CoV-2 non-Spike protein (efficacy against non-Spike seroconversion rates)	**Secondary endpoints:**<br>Endpoints in for the overall population and stratified by COVID-19 serological status at randomisation include:<br>a) VE in preventing virologically-confirmed[c] COVID-19; Per-protocol population analysis. Time frame: include all cases occurring onward from 21 days after a single dose or 7 days after a second dose (if a 2-dose schedule was adopted)<br>b) VE in preventing virologically-confirmed[c] COVID-19 cases<br>VE in preventing virologically-confirmed[c] moderate-severe COVID-19<br>c) VE in preventing hospitalization due to virologically-confirmed[c] COVID-19<br>VE in preventing death associated with virologically- confirmed[c] COVID-19<br>d) VE in preventing] all-cause LRTI (overall and stratified by hospitalization or not, irrespective of test result for SARS-COV-2)

Table 1 Summary of Pivotal Studies to be Pooled

Element	COV001	COV002	COV003	COV005
Efficacy endpoints (continued)	**Exploratory endpoints**:<br>N/A	**Exploratory endpoints**:<br>a) Virologically-confirmed[c] SARS-CoV-2 asymptomatic infection<br>b) Differences in viral loads between those with severe, mild, and asymptomatic virologically-confirmed[c] SARS-CoV-2 infections.<br><br>Where possible, sensitivity analyses will be conducted using common alternative definitions of virologically-confirmed COVID-19 disease, including those in use in other Phase 3 protocols (including but not limited to: USA AstraZeneca Phase 3 trial, South Africa COV005 trial, WHO solidarity trial, CEPI definition).	**Exploratory endpoints**:<br>Where possible, sensitivity analyses will be conducted using common alternative definitions of virologically-confirmed[c] COVID-19 disease, including those in use in other Phase 3 protocols (including but not limited to: USA AstraZeneca Phase 3 trial, South Africa COV005 trial, WHO solidarity trial, CEPI definition).	**Exploratory endpoints:**<br>a) VE in preventing death associated with virologically-confirmed[c] COVID-19<br>b) VE in preventing virologically-confirmed[c] COVID-19 or all-cause LRTI requiring supplemental oxygenation<br>c) VE in preventing virologically-confirmed[c] COVID-19 or all-cause LRTI mechanical ventilation<br>d) VE in preventing virologically-confirmed[c] COVID-19 or all-cause LRTI multi-organ dysfunction syndrome<br>e) VE in preventing virologically-confirmed[c] COVID-19 or all-cause LRTI all-cause mortality<br>f) VE in preventing asymptomatic SARS-CoV-2 infection (samples collected at scheduled study visits); ie, no presence of any of the symptoms contributing to COVID-19 outcome, but virologically-confirmed[c] infection<br>g) VE against sero-conversion suggestive of SARS-CoV-2 infection tested using a N-protein IgG assay

Table 1 Summary of Pivotal Studies to be Pooled

Element	COV001	COV002	COV003	COV005
Planned total enrolment	1122	12390	10000	2070
Control	MenACWY	MenACWY	MenACWY	Saline
Number of doses	One or two (based on study group)	One or two (based on study group)	Two	Two
AZD1222 dose levels[b]	Standard and Low	Standard and Low	Standard and standard	Standard and Low
Prophylactic treatment	Paracetamol for a portion of participants	Paracetamol for a portion of participants	Paracetamol systematically	As clinically needed

[a] This group will be enrolled later.
[b] AZD1222 dose levels are defined in Table 4.
[c] Virologically-confirmed from RT-PCR or other nucleic acid amplification test.
COVID = coronavirus disease 2019; FIH = first-in-human; HIV = human immunodeficiency virus; IgG = immunoglobulin G; LRTI = lower respiratory tract infection; MenACWY = meningococcal Group A, C, W-135 and Y conjugate vaccine; N/A = not applicable; PCR = polymerase chain reaction; PS80 = polysorbate 80; qPCR = quantitative polymerase chain reaction; RT-PCR = reverse transcriptase-polymerase chain reaction; SARS-CoV-2 = severe acute respiratory syndrome coronavirus-2; VE = vaccine efficacy; vp = viral particle.
Source: University of Oxford-sponsored study protocols for COV001 version 10.0, COV002 version 12.0, COV003 version 5.0, COV005 version 4.1.
AZD1222 is referred to as ChAdOx1 nCoV-19 in the University of Oxford study protocols.

4. POOLING OF DATA

Despite minor differences across the studies, there is sufficient consistency to justify the proposal for pooled analyses. Some of the differences were driven by input from investigators, funders, and local specificities. The design of studies to be pooled is summarised in Table 1.

The four Oxford-sponsored studies COV001, COV002, COV003, and COV005 are blinded, randomised, controlled studies that were designed to provide robust evidence of efficacy and safety of the AZD1222 candidate vaccine. The designs are compared in Table 1.

All studies enrolled adults 18 to 55. In addition, Studies COV002 (UK, Phase II/III) and COV003 (Brazil, Phase III), have enrolled older adults in age escalation groups of 56 to 69 years of age and ≥ 70 years of age. Enrolment in COV001 was restricted to healthy adults. The other studies allowed the inclusion of people with underlying health conditions with the exception of severe and/or uncontrolled underlying disease. All studies excluded pregnant and breastfeeding women. The safety and immunogenicity of AZD1222 in adults with known HIV infection was specifically investigated in a small subset of participants in Studies COV002 and COV005).

Collection and assessment of data for capture of COVID 19 variables is performed in a consistent manner across the studies. All participants have good access to health care and cases of COVID 19 are detected through passive surveillance systems. A central, blinded adjudication committee is being used by all 4 studies to assess COVID-19 cases from all participants with SARS-CoV-2 virologically positive results. Each case is assessed by the adjudication committee and classified according to the WHO severity grading scale reproduced in Table 2. The adjudicated results are used for the pooled analyses.

Table 2 WHO Clinical Progression Scale

Patient State	Descriptor	Score
Uninfected	Uninfected; no viral RNA detected	0
Ambulatory mild disease	Asymptomatic; viral RNA detected	1
	Symptomatic; independent	2
	Symptomatic; assistance needed	3
Hospitalised: moderate disease	Hospitalised; no oxygen therapy[a]	4
	Hospitalised; oxygen by mask or nasal prong	5
Hospitalised: severe disease	Hospitalised; oxygen by NIV or high flow	6
	Intubation and mechanical ventilation, pO2/FiO2 ≥ 150 or SpO2/FiO2 ≥ 200	7
	Mechanical ventilation pO2/FIO2 < 150 (SpO2/FiO2 <200) or vasopressors	8
	Mechanical ventilation pO2/FiO2 < 150 and vasopressors, dialysis, or ECMO	9
Dead	Dead	10

[a] If hospitalised for isolation only, record status as for ambulatory patient.
ECMO = extracorporeal membrane oxygenation; FiO2 = fraction of inspired oxygen; NIV = non-invasive ventilation; pO2 = partial pressure of oxygen; SpO2 = oxygen saturation.
Source: (WHO et al 2020).

Case definitions for the pooled analysis are given in Table 6. Per protocol in UK studies ICU admission was a protocol defined endpoint. In order to standardise “ICU admission” across trials for differences in local medical practice, this was redefined as “Requiring ICU admission” and corresponds to those WHO severity grades reflecting the need for mechanical ventilation.

Studies COV001, COV002, COV003, and COV005 are all randomised, controlled studies in healthy volunteers with similar efficacy endpoints. Case detection methods for efficacy assessments are also similar. Access to care for passive case detection is available in all studies; COV002 also includes weekly self-swabs for detection of infection and COV005 includes nasal swab and/or saliva on each scheduled visit. Key inclusion criteria are similar for all studies, except for age. Although there are some differences in the exclusion criteria across the studies, study populations are generally similar. All studies excluded patients with serious conditions or receiving medication that could interfere with the study conduct or the interpretation of study data.

4.1 Planned Analyses

The proposed primary and interim pooled analyses provide an opportunity to produce early and robust estimates of efficacy to support rapid decision making in the present conditions of

this public health emergency. One interim analysis and one primary analysis are planned as below:

The interim analysis will be triggered when 53 COVID-19 cases (SARS-CoV-2 virologically confirmed) that occurred ≥ 15 days post the second dose have been reported in participants who received SD/SD (as defined in Table 4) across the AZD1222 and control groups in pooled studies. This would provide 77% power for the 20% threshold to assume a true vaccine efficacy of 70%. The analysis will include participants who received two doses, with a SD as the second dose (ie, participants who received LD/SD or SD/SD). For an individual study to be included in the pooled analysis of efficacy, a minimum of 5 primary endpoint defined cases must be accrued. For COV002, only cases accruing in efficacy study groups will be included (groups 4, 6, 9, 10).

The primary analysis will be triggered when 105 COVID-19 cases (SARS-CoV-2 virologically confirmed) that occurred ≥ 15 days post the second dose have been reported in participants who received SD/SD (as defined in Table 4) across the AZD1222 and control groups. This would provide 90% power for the 20% threshold to assume a true vaccine efficacy of 60%. The analysis will include participants who received two doses, with the second dose being SD (ie, participants who received LD/SD or SD/SD). For an individual study to be included in the pooled analysis of efficacy, a minimum of 5 primary endpoint defined cases must be accrued. For COV002, only cases accruing in efficacy study groups will be included (groups 4, 6, 9, 10).

Gamma Alpha-Spending function is used to control the overall Type 1 Error at 5%. The planned alpha level is 1.13% for interim analysis and 4.44% for primary analysis. The minimum observed VE if an interim or primary analysis demonstrates evidence of efficacy is 64% and 48%, respectively.

At the time of the interim and primary analysis, the safety of AZD1222 as measured by defined safety endpoints or of potential risk in overall pooled data and in subgroups as appropriate will be evaluated. Note that whereas an individual study must accrue 5 cases to be included in the pooled efficacy analysis, all data from COV001, COV002, COV003 and COV005 available at the time of analysis will contribute to the safety analysis if meet the definitions for populations.

Analysis marked for primary manuscript only in this plan will be conducted by Oxford University.

5. ANALYSIS POPULATIONS

Analysis sets for the pooled and interim analyses are defined in Table 3. The groups/participants meeting any of the conditions below will be excluded from all analysis sets (unless otherwise indicated) regardless if they meet the definition in Table 3.

- Groups without randomization (eg, group 3 of COV001, group 11 of COV002);
- Participants previously vaccinated with a ChAdOx1 vectored vaccine (eg, group 11 of COV002);
- Participants with HIV diagnosed at study start (group 3 of COV005 and group 12 of COV002).
- Children (< 18 years old)

Furthermore, only groups evaluated for efficacy in COV002 (ie, groups 4, 6, 9,10) will be considered in populations for efficacy analysis.

The corresponding control group for group 2d in COV001 is group 2e.

All analyses will be performed by actual treatment received. For primary endpoint, a sensitivity analysis by randomized treatment assignment will be performed.

Table 3 Populations for Analysis

Population	Description
All participants analysis set	All participants screened for the studies, to be used for reporting disposition and screening failures.
Any Dose for Safety	All randomized adult participants who received at least 1 dose of study intervention (AZD1222 or control).<br>Participants who withdraw consent or assent to participate in the study will be included up to the date of their study termination.<br>Erroneously-treated participants (eg, those randomized to treatment A but are actually given treatment B) are accounted for in this analysis set by assigning them to the treatment they actually received. A participant who received at least one dose of AZD1222 is classified as AZD1222. This analysis set will be used for safety analysis.
Any Dose for Efficacy	All participants in Any Dose for Safety but for groups in COV002, only efficacy groups (ie, groups 4, 6, 9,10) will be considered.<br>This analysis set will be used for efficacy analysis.
Dose1 SD for Safety	Only participants who received SD as the first dose of AZD1222 or in corresponding control group in Any Dose for Safety. The treatment assignment will follow the same rule of Any Dose for Safety analysis set. This analysis set will be used for safety analysis.

Table 3 Populations for Analysis

Population	Description
Dose1 SD Seronegative for Efficacy	Only participants seronegative at baseline in Any Dose for Safety who received SD as the first dose of AZD1222 or in corresponding control group, and remain on-study 22 days after their first dose without having had a prior SARS-CoV-2 virologically-confirmed[a] COVID-19 infection. In addition, for groups in COV002, only efficacy groups (ie, groups 4, 6, 9,10) will be considered.<br>The treatment assignment will follow the same rule of Any Dose for Safety analysis set. This analysis set will be used for efficacy analysis.
SDSD + LDSD Seronegative for Efficacy	Only participants seronegative at baseline in Any Dose for Safety who received LD/SD or SD/SD or in the corresponding control group, and remain on-study 15 days after their second dose without having had a prior SARS-CoV-2 virologically-confirmed[a] COVID-19 infection. In addition, for groups in COV002, only efficacy groups (ie, groups 4, 6, 9,10) will be considered.<br>The treatment assignment will follow the same rule of Any Dose for Safety analysis set. This analysis set will be used for the efficacy analysis.
SDSD + LDSD Seronegative ITT for Efficacy	Only participants seronegative at baseline in Any Dose for Safety who received two doses, planned to receive LD/SD or SD/SD or in the corresponding control group, and remain on-study 15 days after their second dose without having had a prior SARS-CoV-2 virologically-confirmed[a] COVID-19 infection. In addition, for groups in COV002, only efficacy groups (ie, groups 4, 6, 9,10) will be considered.<br>Participants will be analysed according to their randomized treatment irrespective of whether they have prematurely discontinued, according to the intent-to-treat principle.<br>This analysis set will be used for the sensitivity analysis of primary endpoint.
SDSD Seronegative for Efficacy	Only participants seronegative at baseline in SDSD + LDSD Seronegative for Efficacy analysis set who received SD/SD or in the corresponding control group, and remain on-study 15 days after their second dose without having had a prior SARS-CoV-2 virologically-confirmed[a] COVID-19 infection.<br>The treatment assignment will follow the same rule of Any Dose for Safety analysis set. This analysis set will be used for the efficacy analysis.

Table 3 Populations for Analysis

Population	Description
SDSD + LDSD for Immunogenicity	Only participants in Any dose for Safety who received LD/SD or SD/SD of AZD1222 or in corresponding control group. Participants without at least one post baseline immunogenicity result will be excluded.<br>The treatment assignment will follow the same rule of Any dose for safety analysis set. This population will be used for the immunogenicity analysis.
SDSD for Immunogenicity	Only participants in Any Dose for Safety who received two SDs of AZD1222 or in corresponding control group. Participants without at least one post baseline immunogenicity result will be excluded.<br>The treatment assignment will follow the same rule of Any dose for safety analysis set. This analysis set will be used for immunogenicity analysis.

[a] Virologically-confirmed from RT-PCR or other nucleic acid amplification test.
ITT = intent-to-treat; LD = low dose; RT-PCR = reverse transcriptase-polymerase chain reaction; SD = standard dose.

6. DOSING REGIMENS AND TREATMENT GROUPS

Across the 4 University of Oxford-sponsored studies, participants were randomized to receive a single dose or two doses of either AZD1222, ranging from 2.2 to 5.0 × 10^{10} vp, or control (as described in Table 4). AZD1222 CTM was sourced from: 1) CBF at the University of Oxford; 2) Advent, Italy, and 3) Cobra Biologics. For control, the MenACWY vaccine was administrated in Studies COV001, COV002, and the first dose of COV003, and 0.9% normal saline (0.9% NaCl) was administered in Study COV005 and the second dose of Study COV003 for participants who received two doses.

Table 4 presents the actual AZD1222 dose levels participants received across the 4 studies by the CTM source and SD or LD designation. For the pooled analyses, a designation of SD or LD was given to each of the AZD1222 dose levels. Generally, 5 × 10^{10} vp or equivalent is designated as a SD, and 2.2 × 10^{10} vp or 2.5 × 10^{10} vp are designated as a LD. Due to a potency miscalculation, participants in Study COV002 groups 1, 2, 4 and 5a who were to receive 5 × 10^{10} vp (Abs260) Advent as the first dose per protocol, received a lower dose of approximately 2 × 10^{10} vp (qPCR). Study COV005 groups 1 and 2 also had the LD vaccine.

Table 4 Actual AZD1222 Dose Levels Received by CTM and HD or LD Designation

AZD1222 Dose Level	SD/LD
2.2 × 10^{10} vp (qPCR) Advent material	LD
2.5 × 10^{10} vp (qPCR) Advent material	LD

AZD1222 Dose Level	SD/LD
3.5 - 6.5 × 10^{10} vp Cobra material	SD
5 × 10^{10} vp (Abs260) Advent material	LD
5 × 10^{10} vp (qPCR) Advent material	SD
5 × 10^{10} vp Advent material	SD
5 × 10^{10} vp CBF material	SD

CTM = Clinical Trial Material; LD = low dose; SD = standard dose; vp = viral particles.

One source of heterogeneity relates to logistical constraints in the context of the rapid conditions in which this clinical program and scale up manufacturing were initiated in parallel, which led to delays in clinical study material availability for second dose vaccinations in studies to be included in the pooled analyses. This resulted in the fact that, for some participants, the interval between the first and second doses (ie, dose schedule) exceeded 1 month. Dose schedule of two doses will be summarized and homogeneity among different dose schedules will be investigated when needed.

7. OVERVIEW OF ANALYSIS CONVENTIONS

7.1 Data Presentation

The planned 5% alpha will be split across the interim and primary analyses as described in Section 4.1. All efficacy analyses will use a 2-sided alpha test unless otherwise stated. P values will be rounded to 4 decimal places. If a p value is less than 0.0001, it will be reported as "< 0.0001". If a p value is greater than 0.9999, it will be reported as "> 0.9999".

All continuous variables will be summarized using descriptive statistics, reporting N, mean, standard deviation, median, minimum, and maximum. As appropriate, minima and maxima will be reported with the same precision as the raw values; medians and means will have one additional decimal place; standard deviation will have 2 additional decimal places. Data will be displayed by treatment group in all listings, as needed. Participants will be uniquely identified in the listings by the combination of study number, study site number, and subject number.

For discrete, or categorical data, percentages will be suppressed when the count is zero. A row denoted as "Missing" will be included in the count tabulations where necessary to account for dropouts or missing values.

All regulatory analyses will be performed using SAS®, Version 9.4 or higher (SAS Institute Inc., Cary, NC).

7.2 Definitions of Subgroups

To explore the implications for efficacy, safety, and immunogenicity among different populations, the following subgroups will be used:

- Age at randomization;
  - 18-64, 65 years and above
  - 18-55, 56-69, 70 years and above (for efficacy analysis of the primary manuscript only)
- Country (UK, Brazil vs South Africa)
- Comorbidity at baseline (at least one comorbidity vs no comorbidity), where comorbidity is BMI $\geq$ 30 kg/m^2 at baseline, Cardiovascular Disorder, Respiratory disease or Diabetes.
- Baseline serostatus (seronegative vs seropositive)

The analyses by each subgroup will be performed for all endpoints (efficacy, safety, and immunogenicity) unless specified otherwise.

Additional subgroups which may be explored include but are not limited to:

- Gender (male, female)
- Race (Asian, Black, White, Mixed, Other, Unknown): only categories with at least 100 individuals exposed will be presented
- Use of prophylactic paracetamol (for analysis of reactogenicity)
- Dose level (LD/SD vs SD/SD)
- Dose schedule (< 6 weeks, 6-8 weeks, 9-11 weeks, $\geq$ 12 weeks)
- Control type (MenACWY, Saline) (for safety only)

7.3 Definition of Baseline

In general, the last non-missing measurement collected prior to the first dose is considered as baseline. If the change from the second dose of study intervention needs be calculated, the last non-missing assessment collected prior to the second dose of study intervention will be considered as baseline.

7.4 Handling of Missing Data

As a general rule, missing data values will not be imputed unless otherwise specified below.

In the summary of AEs by timing relative to each vaccination, if the AE onset date is completely missing, then the AE will be included in summary. If the AE onset date is partially missing, partial AE dates will be handled using the following imputation rules:

- Partial AE start dates where only the year is known:

- If the year is same as the year of dosing:
  - Assume (first dose date + 1 day) if when AE occurred relative to dosing is not available
  - Assume dose date if otherwise
- If the year is not same as the year of dosing, assume January 1 for start date

- Partial AE stop dates where only the year is known: assume December 31 for stop date. If imputed date is greater than the cut-off date of data, use cut-off date instead.
- Partial AE start dates where only the month and year are known:
  - If the year and the month are the same as the year and the month of dosing:
    - Assume (dose date + 1 day) if when AE occurred relative to dosing is not available
    - Assume dose date if otherwise
  - If the year and the month is not same as the year and the month of dosing: assume the first of the month for start date.
- Partial AE stop dates where only the month and year are known: assume the end of the month for stop date

7.5 Reference Start Date and Study Day

Study Day will be calculated from the reference start date and will be used to show start/stop day of assessments and events. Reference start date is defined as the day of the first dose of study drug intervention ie, Day 0.

Study Day will be computed as follows:

- Study Day = (Date of event –Date of first dose of study drug)

In addition, day relative to vaccination will be derived for each vaccination dose. For example, day relative to the first dose will be equal to the Study Day. Day relative to the second dose will start with a value of 0 on the day of the second dose.

7.6 Windowing Conventions

A windowing convention will be used to determine the analysis value for a given study visit for immunogenicity data analyses. The window definitions as following will be used for the immunogenicity.

- A window of ± 14 days from the target day is applied to the following visits: Study Days 28, 56, 90 and 182;
- A window of ± 30 days from the target day is applied to the following visits: Study Days 364;

- A window of ± 42 days from the target day is applied to the following visits: Overall post first dose + 28;

One or more results for a particular immunogenicity variable may be obtained in the same visit window. In such an event, the result with the date closest to the expected visit date will be used in the analysis. In the event that two observations are equidistant from the expected visit date, the later observation will be used in the analysis.

Table 5 Visit Window for Immunogenicity

Dosing Period	Visit	Day Relative to Dose within the Dosing Period[a]	Visit Window (Study Day) Relative to the Dosing Period
Period 1 (Relative to Dose 1)	Baseline[b]	≤ 0	≤ 0
	Day 28	28	14-42
	Day 56	56	42-70
	Day 90	90	76-104
	Day 182	182	168-196
	Day 364	364	334-394
	Overall post first dose + 28	56	14-104
Period 2 (Relative to Dose 2)	Day 28	28	14-42
	Day 56	56	42-70
	Day 90	90	76-104
	Day 182	182	168-196
	Day 364	364	334-394
		**Scheduled Illness Visit**	**Visit Window (Illness Day)**
Illness Visit	Baseline[a]	0	0
	Illness Day 7	7	5-9

[b] For each dosing period, the administration of the study intervention is designated as Study Day 0. For analyses within a period, the study day value is incremented by 1 for each date following the vaccine administration. Dates prior to the vaccine administration are decremented by 1, with the date preceding the vaccine administration designated as Study Day 0.

[c] Where time is available, the time of the collection must be prior to the first dose of study intervention. Day 0 observations taken after the first dose are considered post-baseline values.

[d] For each dosing period, the administration of the study intervention is designated as Study Day 0. For analyses within a period, the study day value is incremented by 1 for each date following the vaccine administration. Dates prior to the vaccine administration are decremented by 1, with the date preceding the vaccine administration designated as Study Day 0.

8. SUBJECT ENROLLMENT AND DISPOSITION

8.1 Subject Identification

Each subject in the pooled analysis will be uniquely identified by the combination of study number, study site number, and subject number.

8.2 Subject Disposition

Disposition will be summarized by treatment group, for overall and each subgroup.

This disposition table of participants will include the number of participants with informed consent, screen failure, in each exclusion criteria from pooled analysis. For each analysis set defined in Section 5, the number and percentage of participants ongoing in study, completed study, discontinued early from study and reasons of discontinuation, and reasons of exclusion in the analysis set will be provided. Only one reason for study discontinuation will be recorded for each discontinued subject.

8.3 Demographics and Baseline Characteristics

Pooled demography and baseline characteristics data will be summarized for Any dose for Safety analysis set, SDSD + LDSD Seronegative for Efficacy analysis set, SDSD Seronegative for Efficacy analysis set, SDSD + LDSD for Immunogenicity analysis set, and SDSD for Immunogenicity by actual treatment.

Demographics:

- Age (years) at randomization
- Age group (18-64 vs ≥ 65 years; 18-55, 56-69 vs ≥ 70 years)
- Gender
- Race (Asian, Black, White, Mixed, Other, Unknown)

Baseline characteristics:

- BMI at baseline (< 30 vs ≥ 30 kg/m^2)
- Serostatus at baseline (Negative, Indeterminate, Positive)
- Cardiovascular Disorder (Yes vs No)
  - Subcategories: chronic heart failure, ischaemic heart disease (including angina), atrial fibrillation, peripheral vascular disease, valvular heart disease, hypertension, myocardial infarction and other
- Respiratory disease (Yes vs No)
  - Subcategories: Chronic obstructive pulmonary disease, Bronchiectasis, Asthma, Other

- Diabetes (Yes, No, Not collected)
  - Subcategories: Type 1 diabetes, Type 2 diabetes not using insulin, Type 2 diabetes using insulin, Other
- Current smoker (Yes vs No)
- Former smoker (Yes, No, Not collected)
- Comorbidity at baseline (at least one comorbidity vs no comorbidity), where comorbidity includes BMI ≥30 kg/m^2 at baseline, Cardiovascular Disorder, Respiratory disease or Diabetes.

Missing due to participant not providing the answer will be presented under category "Missing" while missing due to information not collected in specific study will be presented under category "Not collected".

8.4 Exposure

The exposure information including dose level (ie, SD [only for analysis set based on Dose1 SD], LD/SD, SD/SD), number of dose(s), all available dose schedule for two doses and further categorized dose schedule (< 6 weeks, 6-8 weeks, 9-11 weeks, 12+ weeks) will be summarized by treatment for overall and each study. The summary will be performed for Any Dose for Safety analysis set, Any Dose for Efficacy analysis set, Dose1 SD for Safety analysis set, Dose 1 SD Seronegative for Efficacy analysis set, SDSD + LDSD Seronegative for Efficacy analysis set, SDSD + LDSD for Immunogenicity analysis set, and SDSD for Immunogenicity by actual treatment. The categories for dose schedule may be combined if number of participants are small in some of categories.

A listing of exposure will be provided at the time of the primary analysis.

9. EFFICACY EVALUATIONS

9.1 Efficacy Assessments

Efficacy analysis will be assessed at time planned in Section 4.1. The planned 5% alpha will be split across the interim and primary analyses as described in Section 4.1.

All data from participants with SARS-CoV-2 virologically positive results from RT-PCR or other nucleic acid amplification tests will be assessed by a blinded adjudication committee and the adjudicated endpoints used for all analyses. WHO clinical progression scale (WHO et al 2020) will be utilized to assess the severity of disease. The description of WHO clinical progression scale is in Table 2.

The case for evaluation of efficacy based on adjudicated results are defined as in Table 6.

The overview of the endpoints and corresponding analysis populations is shown in the Appendix A.

Table 6 Case Definitions for Evaluation of Efficacy

Case	Definition
COVID-19 (Primary)<br>Virologically-confirmed[a] symptomatic cases of COVID-19	PCR-confirmed SARS-CoV-2 and at least one of the following symptoms: objective fever (defined as ≥ 37.8 °C), cough, shortness of breath, anosmia, or ageusia. Confirmed by adjudication committee.
COVID-19 Severe Disease	WHO grade ≥ 6[b]
COVID-19 Hospital Admission	WHO grade ≥ 4[b]
COVID-19 Requiring ICU	WHO grade ≥ 7[b]
COVID-19 Death	WHO grade = 10[b]
Asymptomatic SARS-CoV-2 infection	PCR-confirmed SARS-CoV-2 infection and no symptom recorded in data. Confirmed by adjudication committee.

a Virologically-confirmed from RT-PCR or other nucleic acid amplification test.
b WHO clinical progression scale.

9.1.1 Primary Efficacy Endpoint

Incidence of SARS-CoV-2 Virologically-confirmed COVID-19 Occurring ≥ 15 Days Post Second Dose of Study Intervention

The primary efficacy endpoint is the first case of SARS-CoV-2 virologically-confirmed COVID-19 occurring ≥ 15 days post second dose of study intervention. The case is defined as COVID-19 (primary) in Table 6. Only cases with both the sampling date of positive PCR test and COVID-19 symptom(s) onset date ≥ 15 days post second dose will be counted as events. The event date is the date of the PCR test date or date of symptom onset, whichever comes first, and this date will be defined by the endpoint review committee for analysis. For participants with multiple events, only the first occurrence will be used for the primary efficacy endpoint analysis.

The primary efficacy analysis will be based on SDSD + LDSD Seronegative for Efficacy analysis set.

Period at risk for primary endpoint for pooled analysis will be calculated using the reference start as the second dose date + 15 days as Day 1. Calculations for Period at risk are provided below:

Period at risk for primary endpoint = [Last date at risk– (the second dose date + 15 days)] + 1

Where last date at risk will be:

- Date of first event for those diagnosed with SARS-CoV-2 virologically-confirmed COVID-19;
- Date of completion/early discontinuation for participants who complete or discontinue study without event;
- Data cut-off date for analysis for rest of participants who are ongoing without event at the time of analysis.

If the primary endpoint is missing, the period at risk for primary endpoint will be set to missing.

9.1.1.1 Time to Events Endpoints for Supplementary Analysis

Time to First SARS-CoV-2 Virologically-confirmed COVID-19 Occurring ≥ 15 Days Post Second Dose of Study Intervention

Time to First SARS-CoV-2 Virologically-confirmed COVID-19 is defined as the time from the second vaccination + 15 days until the date of the first SARS-CoV-2 virologically-confirmed COVID-19. The calculation is same as the period at risk for the primary endpoint for the pooled analysis. Participants with SARS-CoV-2 virologically-confirmed COVID-19 will be counted as having event while the participants with non-missing value for the period at risk for primary endpoint for pooled analysis will be counted as censored.

Participants with missing value for the period at risk for primary endpoint for pooled analysis will be excluded from analysis.

9.1.2 Secondary Efficacy Endpoints

9.1.2.1 Incidence of Severe COVID-19 Occurring ≥ 15 Days Post Second Dose of Study Intervention

An event is defined as COVID-19 Severe Disease in Table 6, and both the sampling date of positive PCR test and COVID-19 symptom(s) onset date ≥ 15 days post second dose. For participants with multiple events, only the first occurrence will be used for analysis.

9.1.2.2 Incidence of Severe COVID-19 Occurring Post First Dose of Study Intervention

An event is defined as COVID-19 Severe Disease in Table 6, and both the sampling date of positive PCR test and COVID-19 symptom(s) onset date post first dose. For participants with multiple events, only the first occurrence will be used for analysis.

9.1.2.3 Incidence of Severe COVID-19 Occurring ≥ 22 Days Post First Dose of Study Intervention

An event is defined as COVID-19 Severe Disease in Table 6, and both the sampling date of positive PCR test and COVID-19 symptom(s) onset date ≥ 22 days post first dose. For participants with multiple events, only the first occurrence will be used for analysis.

9.1.2.4 Incidence of Asymptomatic SARS-CoV-2 Infection Occurring ≥ 15 Days Post Second Dose of Study Intervention for COV002 Only

An event is defined as Asymptomatic SARS-CoV-2 infection in Table 6 and a PCR-positive sample collected ≥ 15 days post second dose. For participants with multiple events, only the first occurrence will be used for analysis.

The period at risk for SARS-CoV-2 Virologically-confirmed COVID-19 will be calculated similarly to period at risk for the primary endpoint for the pooled analysis replacing information related the primary endpoint for the pooled analysis with information of Asymptomatic SARS-CoV-2 Infection.

The corresponding time to event endpoint is below:

- Time to First Asymptomatic SARS-CoV-2 Infection Occurring ≥ 15 Days Post Second Dose of Study Intervention for COV002 only

9.1.2.5 Incidence of Asymptomatic SARS-CoV-2 Infection Occurring ≥ 22 Days Post First Dose of Study Intervention for COV002 Only

This endpoint will be calculated in the same manner as asymptomatic SARS-CoV-2 infection confirmed by RT-PCR COVID-19 occurring ≥ 15 days post second dose of study intervention for COV002 only (Section 9.1.2.4) by changing the reference date from date of 15 Days post second dose to date of 22 days post first dose and the analysis set will follow Appendix A.

The corresponding time to event endpoint is below:

- Time to Asymptomatic SARS-CoV-2 Infection Confirmed by RT-PCR COVID-19 Occurring ≥ 22 Days Post First Dose of Study Intervention for COV002 only

9.1.2.6 Incidence of SARS-CoV-2 Virologically-confirmed COVID-19 Occurring Post First Dose of Study Intervention

This endpoint will be calculated in the same manner as primary endpoint (Section 9.1.1) by changing the reference date from date of 15 Days post second dose to the first dose date and the analysis will be performed on Any dose for efficacy analysis set.

The corresponding time to event endpoint is below:

- Time to First SARS-CoV-2 Virologically-confirmed COVID-19 Occurring Post First Dose of Study Intervention

9.1.2.7 Incidence of SARS-CoV-2 Virologically-confirmed COVID-19 Occurring 22 Days Post First Dose of Study Intervention

This endpoint will be calculated in the same manner as primary endpoint (Section 9.1.2.1) by changing the reference date from date of 15 Days post second dose to date of 22 days post first dose and the analysis will be performed on Dose1 SD for efficacy analysis set.

The corresponding time to event endpoint is below:

- Time to First SARS-CoV-2 Virologically-confirmed COVID-19 Occurring 22 Days Post First Dose of Study Intervention

9.1.2.8 Duration of Follow-up

For participants in the pooled analysis, the duration of follow-up since the first dose, 22 days post the first dose and 15 days post the second dose will be calculated respectively as study completion/discontinuation date - reference date +1. If subject did not discontinue from the study at the time of the analysis, the data cut-off date will be used. The duration will be summarized by treatment group.

9.1.2.9 Incidence of COVID-19 Hospital Admission Occurring ≥ 15 Days Post Second Dose of Study Intervention

An event is defined as COVID-19 Hospital Admission in Table 6 and with both admission date of hospitalization and onset date of associated COVID-19 are ≥ 15 days post second dose of study intervention.

9.1.2.10 Incidence of COVID-19 Hospital Admission Occurring Post First Dose of Study Intervention

An event is defined as COVID-19 Hospital Admission in Table 6 and with both admission date of hospitalization and onset date of associated COVID-19 are post first dose of study intervention.

9.1.2.11 Incidence of COVID-19 Hospital Admission Occurring 22 Days Post First Dose of Study Intervention

An event is defined as COVID-19 Hospital Admission in Table 6 and with admission date of hospitalization and onset date of associated COVID-19 are ≥ 22 days post first dose of study intervention.

9.1.2.12 Incidence of COVID-19 Death Occurring ≥ 15 Days Post Second Dose of Study Intervention

An event is defined as COVID-19 Death in Table 6 and both death date and onset date of associated COVID-19 are ≥ 15 days post second dose of study intervention.

9.1.2.13 Incidence of COVID-19 Death Associated with SARS-CoV-2 Virologically-confirmed COVID-19 Occurring Post First Dose of Study Intervention

An event is defined as COVID-19 Death in Table 6 and with both death date and onset date of associated COVID-19 are post first dose of study intervention.

9.1.2.14 Incidence of COVID-19 Death Associated with SARS-CoV-2 Virologically-confirmed COVID-19 Occurring 22 Days Post First Dose of Study Intervention

An event is defined as COVID-19 Death in Table 6 and with both death date and onset date of associated COVID-19 are ≥ 22 days post first dose of study intervention.

9.1.2.15 Incidence of COVID-19 Requiring ICU Occurring ≥ 15 Days Post Second Dose of Study Intervention

An event is defined as COVID-19 Requiring ICU in Table 6 and with both admission date and onset date of associated COVID-19 are ≥ 15 days post second dose of study intervention.

9.1.2.16 Incidence of COVID-19 Requiring ICU Occurring Post First Dose of Study Intervention

An event is defined as COVID-19 Requiring ICU in Table 6 and with both admission date and onset date of associated COVID-19 are post first dose of study intervention.

9.1.2.17 Incidence of COVID-19 Requiring ICU Occurring ≥ 22 Days Post First Dose of Study Intervention

An event is defined as COVID-19 Requiring ICU in Table 6 and with both admission date and onset date of associated COVID-19 are ≥ 22 days post first dose of study intervention.

9.1.2.18 Secondary Efficacy Endpoint Based on Nucleocapsid Antibody: Detection of SARS-CoV-2 Nucleocapsid Antibody Levels Over Time

If data available, the detection of SARS-COV-2 nucleocapsid antibody will be summarised as proportion of participants who have a post-treatment response (negative at baseline to positive post treatment with study intervention) to SARS-CoV-2 Nucleocapsid antibodies. This will be summarized by study arm, by visit, overall and by PCR result based on analysis sets for immunogenicity. The window conventions defined in Table 5 will apply to this variable.

9.2 Efficacy Analyses

The efficacy analyses will be conducted in the analysis sets as planned in Table A1Table **3**, unless otherwise specified.

9.2.1 Primary Efficacy Analyses

9.2.1.1 Pooled Analysis of Primary Efficacy Endpoint

A Poisson regression model with robust variance (Zou 2004) will be used as the primary efficacy analysis model to estimate the relative risk (RR) of the incidence of SARS-CoV-2 virologically-confirmed primary symptomatic COVID-19 between the AZD1222 and control groups. The model contains the term of study code, treatment group, and age group at randomization (ie, 18-55 years, 56-69 years, and ≥ 70 years). The logarithm of the period at risk for primary endpoint for pooled analysis will be used as an offset variable in the model to adjust for volunteers having different follow up times during which the events occur.

Vaccine efficacy (VE), which is the incidence of infection in the vaccine group relative to the incidence of infection in the control group expressed as a percentage, will be calculated as VE = 1- relative risk. The VE, and its corresponding 2-sided (1-α) % confidence interval (CI), will be estimated from the model. In addition, the 2-sided p value testing null hypothesis that the incidence of SARS-CoV-2 virologically-confirmed primary symptomatic COVID-19 between AZD1222 and control groups are the same will be obtained from the model. Statistical significance will be achieved if the 2-sided p value is ≤ α, where α for interim and primary analysis as descripted in Section 4.1.

The Poisson regression with robust variance analysis will be implemented by using the SAS PROC GENMOD procedure for binary data with the REPEATED statement for subject ID and logarithm link. The estimated parameter $\hat{\beta}$ [ie, log ($\widehat{RR}$)], 2-sided (1-α) % CI for $\hat{\beta}$, and the 2-sided p value will be obtained from the SAS outputs. The estimated RR and corresponding CI for the RR is given by exponentiating $\hat{\beta}$ and its confidence limits. Therefore, the percent of VE is given by [(1 – exp ($\hat{\beta}$)) * 100%]. The CI for the percent of VE is given by ([1 – exp(upper confidence limit for $\hat{\beta}$) * 100%], [1 – exp(lower confidence limit for $\hat{\beta}$) * 100%]).

If the Poisson regression model with robust variance fails to converge, the exact conditional method for stratified Poisson regression using PROC GENMOD with the exact statement will be used.

To investigate if the pooled studies are homogeneous, a study-by-treatment interaction term may be included in the Poisson regression model as a factor and the type III p value for this term will be presented. If the p value is greater than or equal to 0.05, the study-by-treatment interaction term may be dropped from the model for presentation of summary analysis results. Same method may be performed for dose schedule (< 6 weeks, 6-8 weeks, 9-11 weeks, 12+ weeks), and dose pattern (LD/SD, SD/SD) if further exploration is warranted.

9.2.1.2 Secondary Analysis of Primary Endpoint

The analyses for the primary endpoint for the pooled analysis will be repeated for participants who received two SDs of vaccine (ie, SDSD Seronegative for Efficacy analysis set).

9.2.1.3 Subgroup Analysis of Primary Endpoint

Subgroup analysis will be performed for the primary efficacy endpoint, the incidence of SARS-CoV-2 virologically-confirmed COVID-19. Within each level of a subgroup, the VE and its corresponding (1-α) % CI will be estimated using a Poisson regression model with robust variance with the term of treatment. A forest plot of the VE and the (1-α) % CI will be presented. If the Poisson regression model does not converge for any stratum of a subgroup, the exact conditional method for stratified Poisson regression using PROC GENMOD with the exact statement will be used.

9.2.1.4 Supportive Analyses for Primary Efficacy Endpoint

To support the primary analysis, a Cox Proportional Hazards model using the same covariates as for the primary analyses as well as Kaplan-Meier curves will be presented for the active and control groups based on observed events, showing the cumulative incidence of the first case of SARS-CoV-2 RT-PCR-positive symptomatic illness occurring ≥ 15 days post second dose of study intervention. Time to event, i.e., the duration in days since 15 days post second study dose to event or censoring, will be fit using the PH model with treatment as a factor and age group, country as stratum. Hazards ratios for each study arm along with the two-sided (1-α) % CI will be obtained from the PH model. The number of participants with a primary endpoint and the number of censored participants will also be provided. The censoring timing at each month will be displayed.

9.2.1.5 Sensitivity Analysis of Primary Endpoint

The analyses for the primary endpoint for the pooled analysis will be repeated for participants who received two SDs of vaccine and the second dose is SD based on randomized treatment (ie, SDSD + LDSD Seronegative ITT for Efficacy analysis set).

9.2.2 Secondary Efficacy Analyses

9.2.2.1 Incidence of Asymptomatic SARS-CoV-2 Infection COV002 only

The analysis for those endpoints will be conducted in a similar manner as described in Section 9.2.1.1 (see Pooled Analysis of Primary Efficacy Endpoint) but remove Study code from model.

9.2.2.2 Duration of Follow-up since the Second Vaccination

Summary by treatment group will be calculated for this variable.

9.2.2.3 Other Secondary Efficacy Endpoints

The remaining endpoints will be analysed in the same manner as the pooled analysis of the primary efficacy endpoint as described in Section 9.2.1.1 (see Pooled Analysis of Primary Efficacy Endpoint). VE will not be computed unless 5 cases have accrued for the respective endpoint, but the distribution by groups will be presented.

9.2.2.4 Secondary Efficacy Analyses for Endpoints Based on Nucleocapsid: Detection AntibodySARS-CoV-2 Nucleocapsid Related Endpoints

Percentage of participants positive for SARS-CoV-2 nucleocapsid antibody levels over time will be summarized overall and by baseline seropositivity status. At each time point the % positive for nucleocapsid antibody will be summarized for those who were not previously positive at any time point and in those who were previously positive at least one time point.

10. SAFETY ANALYSIS

Safety summaries described below will be performed by treatment group (AZD1222 and Control) for analysis sets planned in Table A2.

10.1 Adverse Events

10.1.1 Reporting of Adverse events

All local and systemic AEs that occur within 28 days after each vaccination observed by the Investigator or reported by the participant, will be recorded by the participants in the Diary of Symptoms and by the Investigators in the study CRF. For COV001, diary cards for the second vaccines will not be filled out for participants in groups 2f, 2g, 4c and 4d. For COV002, solicited and unsolicited AEs will be reported for 7 days only for group 1-3, 5, 7, 8, 11 and 12 and a subset of up to 3000 participants for groups 4, 6, 9, and 10. In COV003, solicited AEs will be reported for a subset of 200 participants.

SAEs and Adverse Events of Special Interest will be collected throughout the study period.

Adverse events will be coded using the Medical Dictionary for Regulatory Activities (MedDRA) Version 23 or higher.

10.1.2 Evaluation of Severity

The severity of AEs will be assessed according to toxicity rating scales adapted from the FDA for healthy volunteers recruited in preventive vaccine clinical trials, listed in the specific study work instructions and Table 7. For COV005, DAIDS grading the severity of adult and paediatric adverse events is used. For solicited AE, refer to Appendix B.

Table 7 Severity Rating Criteria for Physical Observations (Applies to Adults Only) for COV001, COV002 and COV003

Vital Signs	Grade 1 (mild)	Grade 2 (moderate)	Grade 3 (serious)	Grade 4 Potentially fatal
Fever (oral)	38, 0°C- 38, 4°C	38.5°C - 38.9°C	39.0°C - 40°C	> 40°C
Tachycardia (bpm)[a]	101 – 115	116-130	> 130	A/E visit or hospitalization for arrhythmia
Bradycardia (bpm)[b]	50 – 54	45 – 49	< 45	A/E visit or hospitalization for arrhythmia
Systolic hypertension (mmHg)	141 -150	151 – 155	≥ 155	A/E visit or hospitalization for malignant hypertension
Diastolic hypertension (mmHg)	91 – 95	96 – 100	> 1100	A/E visit or hospitalization for malignant hypertension
Systolic hypotension (mmHg)[c]	85 – 89	80 – 84	< 80	A/E visit or hospitalization for hypotensive shock
Respiratory Rate - breaths per minute	17 – 20	21-25	> 25	Intubation

[a] Measured after ≥10 minutes at rest
[b] When the resting heart rate is between 60 to 100 beats per minute. Use the clinical criterion when characterizing bradycardia among some populations of healthy participants, for example, conditioned athletes.
[c] Only if symptomatic (for example, dizziness/vertigo)
A/E = accident & emergency department.

Table 8 Severity Grading Criteria for Select Physical Observations (Based on DAIDS Grading Table; Version 2.1 – July 2017 for COV005)

Vital signs	Grade 1 (mild)	Grade 2 (moderate)	Grade 3 (severe)	Grade 4 Potentially life threatening
	Mild symptoms causing no or minimal interference with usual social & functional activities with intervention not indicated	Moderate symptoms causing greater than minimal interference with usual social & functional activities with intervention indicated	Severe symptoms causing inability to perform usual social & functional activities with intervention or hospitalization indicated	Potentially life-threatening symptoms causing inability to perform basic self-care functions with intervention indicated to prevent permanent impairment, persistent disability, or death

Table 8 Severity Grading Criteria for Select Physical Observations (Based on DAIDS Grading Table; Version 2.1 – July 2017 for COV005)

Vital signs	Grade 1 (mild)	Grade 2 (moderate)	Grade 3 (severe)	Grade 4 Potentially life threatening
Arthralgia	Joint pain causing no or minimal interference with usual social & functional activities	Joint pain causing greater than minimal interference with usual social & functional activities	Joint pain causing inability to perform usual social & functional activities	Disabling joint pain causing inability to perform basic self-care functions
Arthritis	Stiffness or joint swelling causing no or minimal interference with usual social & functional activities	Stiffness or joint swelling causing greater than minimal interference with usual social & functional activities	Stiffness or joint swelling causing inability to perform usual social & functional activities	Disabling joint stiffness or swelling causing inability to perform basic self-care functions
Chills	Symptoms causing no or minimal interference with usual social & functional activities	Symptoms causing greater than minimal interference with usual social & functional activities	Symptoms causing inability to perform usual social & functional activities	NA
Fatigue or Malaise *Report only one*	Symptoms causing no or minimal interference with usual social & functional activities	Symptoms causing greater than minimal interference with usual social & functional activities	Symptoms causing inability to perform usual social & functional activities	Incapacitating symptoms of fatigue or malaise causing inability to perform basic self-care functions
Fever (non-axillary temperatures only)	38.0 to < 38.6°C	≥ 38.6 to < 39.3°C	≥ 39.3 to < 40.0°C	≥ 40.0°C or ≥ 104.0°F
Headache	Symptoms causing no or minimal interference with usual social & functional activities	Symptoms causing greater than minimal interference with usual social & functional activities	Symptoms causing inability to perform usual social & functional activities	Symptoms causing inability to perform basic self-care functions OR Hospitalization indicated OR Headache with significant impairment of alertness or other neurologic function

Table 8 Severity Grading Criteria for Select Physical Observations (Based on DAIDS Grading Table; Version 2.1 – July 2017 for COV005)

Vital signs	Grade 1 (mild)	Grade 2 (moderate)	Grade 3 (severe)	Grade 4 Potentially life threatening
Myalgia (generalized)	Muscle pain causing no or minimal interference with usual social & functional activities	Muscle pain causing greater than minimal interference with usual social & functional activities	Muscle pain causing inability to perform usual social & functional activities	Disabling muscle pain causing inability to perform basic self-care functions
Pain (not associated with study agent injections and not specified elsewhere) *Specify location*	Pain causing no or minimal interference with usual social & functional activities	Pain causing greater than minimal interference with usual social & functional activities	Pain causing inability to perform usual social & functional activities	Disabling pain causing inability to perform basic self-care functions OR Hospitalization indicated
Acute Allergic Reaction	Localized urticaria (wheals) with no medical intervention indicated	Localized urticaria with intervention indicated OR Mild angioedema with no intervention indicated	Generalized urticaria OR Angioedema with intervention indicated OR Symptoms of mild bronchospasm	Acute anaphylaxis OR Life-threatening bronchospasm OR Laryngeal edema
Blood Pressure Abnormalities Hypertension (with the lowest reading taken after repeat testing during a visit) ≥ 18 years of age	140 to < 160 mmHg systolic OR 90 to < 100 mmHg diastolic	≥ 160 to <180 mmHg systolic OR ≥ 100 to < 110 mmHg diastolic	≥ 180 mmHg systolic OR ≥ 110 mmHg diastolic	Life-threatening consequences in a participant not previously diagnosed with hypertension (eg, malignant hypertension) OR Hospitalization indicated
Hypotension	No symptoms	Symptoms corrected with oral fluid replacement	Symptoms AND IV fluids indicated	Shock requiring use of vasopressors or mechanical assistance to maintain blood pressure

Table 8 Severity Grading Criteria for Select Physical Observations (Based on DAIDS Grading Table; Version 2.1 – July 2017 for COV005)

Vital signs	Grade 1 (mild)	Grade 2 (moderate)	Grade 3 (severe)	Grade 4 Potentially life threatening
Dyspnea or Respiratory Distress *Report only one*	Dyspnea on exertion with no or minimal interference with usual social & functional activities OR Wheezing OR Minimal increase in respiratory rate for age	Dyspnea on exertion causing greater than minimal interference with usual social & functional activities OR Nasal flaring OR Intercostal retractions OR Pulse oximetry 90 to < 95%	Dyspnea at rest causing inability to perform usual social & functional activities OR Pulse oximetry < 90%	Respiratory failure with ventilator support indicated (eg, CPAP, BPAP, intubation)

10.1.3 Solicited AEs

Diary cards will collect the timing and severity of predefined Solicited AEs listed in Appendix B. Solicited local AEs and solicited systemic AEs collected for 7 days (Day 0 to Day 6 for COV005 while Day 0 to Day 7 for rest of studies) after vaccination for each dose will be summarized respectively. The strategy for pooling the data across different studies is provided in Appendix B.

Each solicited AE will be summarized by treatment group at the following time intervals: Days 0-7, and Days 0 to day 7 individually (for PA only). Each time interval will be repeated for after dose 1, after dose 2 and after any dose. For each time interval, the count and percentage of participants will be determined for each of the following categories: participants evaluated, participants with any event, mild events, moderate events, severe events, and potentially life-threatening events. Participants should not be double counted; therefore, the event of greatest severity will be used for participants with more than one episode of the same event. Similar counts and percentages will be presented for solicited local AEs “Overall” and solicited systemic AEs “Overall”. For participants evaluated for diary cards, missing values will be treated as missing in the calculation.

A listing of all solicited AEs will be provided at the time of the primary analysis.

10.1.4 Unsolicited AEs

All AEs are unsolicited AEs unless categorized as solicited AEs. Unsolicited AEs from the start of each dose through 28 days (ie, day of vaccination and the following 27 days) will be summarized. Summaries will be produced of:

- Number of participants with unsolicited AEs by treatment group, system organ class and preferred term
- Number of participants with unsolicited AEs, assessed by investigator as possibly related to investigational product, by treatment, system organ class and preferred term
- Number of participants with unsolicited AEs ≥x% in either treatment group, by treatment and preferred term where x may be 1, 3 or 5

In the summary of number of participants, should a subject experience multiple events within a SOC or PT, the subject will be counted only once for that SOC or PT.

A listing of all unsolicited AEs will be provided at the primary analysis.

10.1.5 Serious Adverse Events (SAEs)

SAEs are those events recorded as "Serious" on the AE page of the eCRF. SAEs following the first vaccination to 364 days after the last vaccination will be summarized. Summaries will be produced of:

- Number of SAEs by treatment group, Medical Dictionary for Regulatory Activities (MedDRA) system organ class and preferred term
- Number of participants with SAEs by treatment group, system organ class and preferred term
- Number of participants with SAEs, assessed by investigator as possibly related to investigational product, by treatment group, system organ class and preferred term
- Number of participants with SAEs ≥ x% in either treatment group, by treatment and preferred term where x may be 1, 3 or 5

In the summary of number of participants, should a subject experience multiple events within a SOC or PT, the subject will be counted only once for that SOC or PT.

A listing of all SAEs including those prior to the first vaccination will be provided at the time of the primary analysis.

10.1.6 Adverse Events of Special Interest

AZD1222 AESIs are based on Brighton Collaboration case definitions (SPEAC 2020), clinical experience, and scientific interest. There is no current evidence to suggest that AZD1222 is associated with these AESIs.

Table 9 Adverse Events of Special Interest

AESI	Medical Concept
Neurologic	Generalized convulsion: Seizures are episodes of neuronal hyperactivity most commonly resulting in sudden, involuntary muscular contractions. They may also manifest as sensory disturbances, autonomic dysfunction and behavioral abnormalities, and impairment or loss of consciousness.
	Other neurologic events: These events would include new onset event (acute or subacute) motor and sensory disturbances (eg, weakness, numbness, paresthesias, hypoesthesia, hyperesthesia, dysesthesias), bowel/bladder dysfunction, gait impairment, or visual disturbance, or other sudden neurological deficit.
Vascular	Thrombotic, thromboembolic, and neurovascular events: These are events that can manifest as transient or permanent vision problems, dizziness, trouble understanding, facial droop, slurred speech, unilateral weakness, deep vein thrombosis with swollen, warm or painful leg, pulmonary embolism with shortness of breath, chest pain or irregular heart rate
Hematologic	Thrombocytopenia: Thrombocytopenia is a disorder in which there is an abnormally low platelet count; a normal platelet count ranges from 150 000 to 450 000 platelets per μL.
Immunologic	Vasculitides: Vasculitides are a group of related disorders characterized by inflammation of blood vessels (vasculitis) leading to tissue or end-organ injury.
	Anaphylaxis: Anaphylaxis an acute hypersensitivity reaction with multi-organ-system involvement that can present as, or rapidly progress to, a severe life-threatening reaction requiring immediate medical attention.
	Vaccine-associated enhanced respiratory disease: The pathogenicity of VAERD has been linked to a vaccine immune response characterized by induction of non-neutralizing antibodies, and a T-cell response of the Th2 type with hypereosinophilia (Lambert et al 2020). VAERD may manifest as a severe form of respiratory disease with prolonged fever, and diverse clinical manifestations of disease severity and pathological changes marked by increased areas of lung consolidation, broncho-interstitial pneumonia, and necrotizing bronchiolitis (Rajão et al 2016).
	Potential immune-mediated conditions: These conditions are a group of autoimmune inflammatory disorders characterized by an alteration in cellular homeostasis, which may or may not have an autoimmune aetiology. A list of events is provided below<br>• Gastrointestinal disorders<br>○ Celiac disease<br>○ Crohn's disease<br>○ Ulcerative colitis<br>○ Ulcerative proctitis

AESI	Medical Concept
	• Liver disorders<br>o Autoimmune cholangitis<br>o Autoimmune hepatitis<br>o Primary biliary cirrhosis<br>o Primary sclerosing cholangitis<br><br>• Metabolic diseases<br>o Addison's disease<br>o Autoimmune thyroiditis (including Hashimoto thyroiditis)<br>o Diabetes mellitus type I<br>o Grave's or Basedow's disease<br><br>• Musculoskeletal disorders<br>o Antisynthetase syndrome<br>o Dermatomyositis<br>o Juvenile chronic arthritis (including Still's disease)<br>o Mixed connective tissue disorder<br>o Polymyalgia rheumatic<br>o Polymyositis<br>o Psoriatic arthropathy<br>o Relapsing polychondritis<br>o Rheumatoid arthritis<br>o Scleroderma, including diffuse systemic form and CREST syndrome<br>o Spondyloarthritis, including ankylosing spondylitis, reactive arthritis<br>o (Reiter's Syndrome) and undifferentiated spondyloarthritis<br>o Systemic lupus erythematosus<br>o Systemic sclerosis

AESI	Medical Concept
	• Neuroinflammatory disorders<br>o Acute disseminated encephalomyelitis, including site specific variants (eg, non-infectious encephalitis, encephalomyelitis, myelitis, radiculomyelitis)<br>o Cranial nerve disorders, including paralyses/paresis (eg, Bell's palsy)<br>o Guillain-Barré syndrome, including Miller Fisher syndrome and other variants<br>o Immune-mediated peripheral neuropathies and plexopathies, including chronic inflammatory demyelinating polyneuropathy, multifocal motor neuropathy and polyneuropathies associated with monoclonal gammopathy<br>o Multiple sclerosis<br>o Neuromyelitis optica spectrum disorder<br>o Narcolepsy<br>o Optic neuritis<br>o Transverse myelitis<br>o Myasthenia gravis, including Eaton-Lambert syndrome<br><br>• Skin disorders<br>o Alopecia areata<br>o Autoimmune bullous skin diseases, including pemphigus, pemphigoid and dermatitis herpetiformis<br>o Cutaneous lupus erythematosus<br>o Erythema nodosum<br>o Morphoea<br>o Lichen planus<br>o Psoriasis<br>o Rosacea<br>o Sweet's syndrome<br>o Vitiligo<br>• Vasculitides<br>o Large vessels vasculitis including: giant cell arteritis such as Takayasu's arteritis and temporal arteritis<br>o Medium sized and/or small vessels vasculitis including: polyarteritis nodosa, Kawasaki's disease, microscopic polyangiitis, Wegener's granulomatosis, Churg– Strauss syndrome (allergic granulomatous angiitis), Buerger's disease, thromboangiitis obliterans, necrotizing vasculitis and anti-neutrophil cytoplasmic antibody (ANCA) positive vasculitis (type unspecified), Henoch-Schonlein purpura, Behcet's syndrome, leukocytoclastic vasculitis

AESI	Medical Concept
	• Other<br>○ Antiphospholipid syndrome<br>○ Autoimmune hemolytic anemia<br>○ Autoimmune glomerulonephritis (including IgA nephropathy, glomerulonephritis rapidly progressive, membranous glomerulonephritis, membranoproliferative glomerulonephritis, and mesangioproliferative glomerulonephritis)<br>○ Autoimmune myocarditis/cardiomyopathy<br>○ Autoimmune thrombocytopenia<br>○ Goodpasture syndrome<br>○ Idiopathic pulmonary fibrosis<br>○ Pernicious anemia<br>○ Raynaud's phenomenon<br>○ Sarcoidosis<br>○ Sjögren's syndrome<br>○ Stevens-Johnson syndrome<br>○ Uveitis

ADEM = acute disseminated encephalomyelitis; AESI = adverse event of special interest; GBS = Guillain-Barré syndrome; VAERD = vaccine-associated enhanced respiratory disease.

This list may be updated along with the studies.

AESIs following the first vaccination to 364 days after the last vaccination will be summarized. The number and percentage of participants with AESIs will be summarized by treatment group, and by treatment, AESI term, and PT. Should a participant experience multiple events within a PT, the participant will be counted only once for that PT according to the first onset date.

A listing of all AESIs will be provided at the primary analysis.

10.1.7 Death

Listings of all death due to causes other than COVID-19 will be provided at the time of the primary analysis.

10.2 Clinical Laboratory Results

Clinical laboratory results on scheduled visits for COV001, COV002 and COV005 (group 1 and 3 only) will be pooled to examine the most extreme value encoded in the 30 days post dose 1 and 30 days post dose 2 by treatment group and severity grade. The strategy for severity grading across different studies is provided in Appendix C.

The clinical laboratory tests include:

- Haematology; Full Blood Count
- Biochemistry; Sodium, Potassium, Urea, Creatinine, Albumin, Liver Function Tests (ALT, ALP, Bilirubin)

A listing for clinical laboratory results and a listing for laboratory results with toxicity grading scale ≥ 3 will be provided at the primary analysis.

10.3 Subgroup Analysis

Overview of AEs including factors below will be summarized by subgroup.

- Incidence of all AEs
- Incidence of solicited AEs
- AEs by severity
- Incidence of unsolicited AEs
- Incidence of SAEs
- Incidence of AESI
- Deaths other than due to COVID-19

11. IMMUNOGENICITY ANALYSIS

Immunogenicity analysis will be based on participants with data available. Summary by actual treatment for overall and by serostatus group (Seronegative, Seropositive) for analysis sets planned in Appendix A.

The immunogenicity endpoints are:

- SARS-CoV-2 S, RBD antibody quantification
- Virus NAb assays against SARS-CoV-2
- Antibody seroconversion rate (≥ 4-fold increase from baseline) against SARS-CoV-2 S protein, RBD and NAb

A multiplexed immunoassay that assesses SARS-CoV-2 S, RBD, and N antigens, will be utilised to determine the antibody responses to AZD1222 vaccination. GMTs and GMFRs of these antigens may be provided at baseline and 28 days after each dose in participants for whom data are available.

Data on neutralising antibodies will be provided in the form of a pseudoneutralisation assay, with GMTs and GMFRs reported at the same timepoints as above.

The proportion of participants who have a post-treatment seroresponse (≥ 4-fold rise in titers from the day of dosing baseline value to 28 days post each dose) to AZD1222 as measured by SARS-CoV-2 binding or neutralising antibodies will also be provided, as data are available. For neutralization assays the proportion with neutralizing titres (> LLOQ) will be generated for the primary manuscript.

As bandwidth of laboratories allows, live virus neutralisation and ELISpot data will complement the data provided. These data will be stratified by age (category 1: 18-55, 56-69, and ≥ 70 year-old participants for primary manuscript and category 2: 18-64 and ≥ 65 year-old participants), baseline serostatus and provided following validation of the assays and testing.

Descriptive statistics for GMTs and GMFRs will include number of participants, geometric mean, 95% CI, minimum and maximum. Medians, 25th quartiles and 75% quartiles may also be presented.

The GMT will be calculated as the antilogarithm of Σ (log 2 transformed titer/n), ie, as the antilogarithm transformation of the mean of the log-transformed titer, where n is the number of participants with titer information. The 95% CI will be calculated as the anti-logarithm transformation of the upper and lower limits for a two-sided CI for the mean of the log-transformed titers.

The fold rise is calculated as the ratio of the post-vaccination titer level to the pre-vaccination titer level, ie, the baseline level. GMFR will be calculated as anti-logarithm of Σ (log 2 transformed (post-vaccination titer/ pre-vaccination titer)/n). The 95% CIs for GMFR will be calculated similarly to those for GMT.

Results reported as lower than the LLOQ for SARS-CoV-2 S and RBD responses and virus neutralizing antibody responses will have a value equal to half of the LLOQ imputed in the calculation.

Immunogenicity data will be presented in a listing at the time of the primary analysis.

12. REFERENCES

FDA. (Food and Drug Administration). Guidance for Industry. Toxicity grading scale for healthy adult and adolescent volunteers enrolled in preventive vaccine clinical trials. . https://www.fda.gov/media/73679/download. Published 2007. Accessed 20 June 2020.

Folegatti PM, Ewer KJ, Aley PK, Angus B, Becker S, Belij-Rammerstorfer S et al. Safety and immunogenicity of the ChAdOx1 nCoV-19 vaccine against SARS-CoV-2: a preliminary report of a phase 1/2, single-blind, randomised controlled trial. Lancet. 2020;S0140-6736(20):31604-4.

Lambert P-H, Ambrosino DM, Andersen SR, Baric RS, Black SB, Chen RT et al. Consensus summary report for CEPI/BC March 12–13, 2020 meeting: Assessment of risk of disease enhancement with COVID-19 vaccines. Vaccine. 2020;38(31):4783-91.

NIH. (National Institutes of Health) National Institute of Allergy and Infectious Diseases, Division of AIDS. Division of AIDS (DAIDS) Table for Grading the Severity of Adult and Pediatric Adverse Events, Corrected Version 2.1 [July 2017]. https://rsc.niaid.nih.gov/sites/default/files/daidsgradingcorrectedv21.pdf. Published 2017. Accessed 13 November 2020.

Rajão DS, Chen H, Perez DR, Sandbulte MR, Gauger PC, Loving CL et al. Vaccine-associated enhanced respiratory disease is influenced by haemagglutinin and neuraminidase in whole inactivated influenza virus vaccines. The Journal of general virology. 2016;97(7):1489-99.

SPEAC. (Safety Platform for Emergency Vaccines) D2.3 Priority list of adverse events of special interest: COVID-19. Work Package: WP2 Standards and Tools. v1.1. 05 March 2020. https://media.tghn.org/articles/COVID-19_AESIs_SPEAC_V1.1_5Mar2020.pdf. Published 2020. Accessed 14 June 2020.

van Doremalen N, Lambe T, Spencer A, Belij-Rammerstorfer S, Purushotham JN, Port JR et al. ChAdOx1 nCoV-19 vaccination prevents SARS-CoV-2 pneumonia in rhesus macaques. bioRxiv. 2020;2020.05.13.093195.

WHO, Marshall JC, Murthy S, Diaz J, Adhikari NK, Angus DC et al. Working Group on the Clinical Characterisation and Management of COVID-19 Infection. A minimal common outcome measure set for COVID-19 clinical research. Lancet Infec Dis. 2020;20(8):e192-7.

Zou G. A modified poisson regression approach to prospective studies with binary data. Am J Epidemiol. 2004;159(7):702-6.

13. APPENDICES

Appendix A Overview of Analyses

Table A1 Overview of Evaluation of Efficacy

Dose and regimen[a]	Population	N[b]	Time period of endpoints	Case definition	Other case definition
SDSD + LDSD	18 years and above, Seronegative	~19000[c]	From 15 days post dose 2	Incidence of First SARS-CoV-2 virologically-confirmed[d] COVID-19	Severe COVID-19<br>Hospitalised COVID-19<br>ICU COVID-19<br>Death COVID-19<br>Infection SARS-CoV-2[e]
SDSD	18 years and above, Seronegative	~15600	From 15 days post dose 2	Incidence of First SARS-CoV-2 virologically-confirmed[d] COVID-19	Severe COVID-19<br>Hospitalised COVID-19<br>ICU COVID-19<br>Death COVID-19<br>Infection SARS-CoV-2[e]
First dose is SD	18 years and above, Seronegative	~15800	From 22 days post dose 1	Incidence of First SARS-CoV-2 virologically-confirmed[d] COVID-19	Severe COVID-19<br>Hospitalised COVID-19<br>ICU COVID-19<br>Death COVID-19<br>Infection SARS-CoV-2[e]
Any dose[e]	18 years and above	~19200	From dose 1	Incidence of First SARS-CoV-2 virologically-confirmed[d] COVID-19	Severe COVID-19<br>Hospitalised COVID-19<br>ICU COVID-19<br>Death COVID-19

[a] by treatment received
[b] Seronegative is not considered. The dose information is based on the spreadsheet from Oxford in Aug2020. Number is floored to 100.
[c] Sensitivity analysis of ITT by randomization to AZD1222 or control
[d] In a subset (COV002)
[e] Virologically-confirmed from RT-PCR or other nucleic acid amplification test.
[f] Required for safety evaluation

COVID-19 = coronavirus disease 2019; ICU = intensive care unit; LD = low dose; SARS-CoV-2 = severe acute respiratory syndrome-coronavirus 2; SD = standard dose.

Table A2 Overview of Evaluation of Safety

Dose and regimen[a]	Population	N[b]	Endpoints
First dose is SD	18+	~16000	Reactogenicity<br>Unsolicited AEs<br>SAEs<br>AESIs
Any dose	18+	~19800	Reactogenicity<br>Unsolicited AEs<br>SAEs<br>AESIs

a by treatment received
b Seronegative is not considered. The dose information is based on the spreadsheet from Oxford in Aug2020. Number is floored to 100.
AE = adverse event; AESI = adverse event of special interest; SAE = serious adverse event; SD = standard dose.

Table A3 Overview of Evaluation of Immunogenicity

Dose and regimen[a]	Population	N[b]	Endpoints	Note
SDSD + LDSD	18+	~15600	Spike<br>RBD<br>NAb	By serostatus
SDSD	18+	~19200	Spike<br>RBD<br>NAb	By serostatus

a by treatment received
b Seronegative and non-missing post baseline value is not considered. The dose information is based on the spreadsheet from Oxford in Aug2020. Number is floored to 100.
LD = low dose; SD = standard dose.

Appendix B Pooling and Severity Grading of Solicited Adverse Events: Studies COV001, COV002, COV003, and COV005

B 1 Introduction

In AZD1222 Studies COV001, COV002, COV003, and COV005, diary cards collect predefined local and systemic AEs that are commonly associated with vaccine administration, including the severity of these events. Although there are many similarities in the collection of solicited AEs in these studies, there are also differences. This document presents the similarities and differences in solicited event collection across these studies and a strategy for pooling events across studies.

B 2 Frequency of Solicited Adverse Event Entries

Table B1 presents the schedule of solicited AEs reporting by participants in the patient diaries and the protocol text describing the relevant outcome measures and timepoints, when available in Studies COV001, COV002, COV003, and COV005.

Table B1 Schedule and Protocol Description of Solicited Adverse Event Reporting in Patient Diaries

Study	1st vaccination									2nd vaccination							
COV001	Day 0	Day 1	Day 2	Day 3	Day 4	Day 5	Day 6	Day 7		Day 0	Day 1	Day 2	Day 3	Day 4	Day 5	Day 6	Day 7
- CSP text	Outcome measure: Occurrence of solicited local/systemic reactogenicity signs and symptoms for 7 days following vaccination: Day 0-7 Self-reported symptoms recorded using electronic diaries.																
COV002	Day 0	Day 1	Day 2	Day 3	Day 4	Day 5	Day 6	Day 7		Day 0	Day 1	Day 2	Day 3	Day 4	Day 5	Day 6	Day 7
- CSP text	Outcome measure: Occurrence of solicited systemic reactogenicity signs and symptoms for 7 days following vaccination/booster vaccination: Day 0-7 Self-reported symptoms recorded using electronic diaries																
COV003	Day 0	Day 1	Day 2	Day 3	Day 4	Day 5	Day 6	Day 7		Day 0	Day 1	Day 2	Day 3	Day 4	Day 5	Day 6	Day 7
- CSP text	Endpoint measure: Occurrence of signs and symptoms of local and systemic reactogenicity requested during 7 days after vaccination																
COV005	Day 0	Day 1	Day 2	Day 3	Day 4	Day 5	Day 6	-		Day 0	Day 1	Day 2	Day 3	Day 4	Day 5	Day 6	-
- CSP text	Endpoint measure: occurrence of solicited local/systemic reactogenicity signs and symptoms for 7 days following vaccination;																

CSP = clinical study protocol.

B 3 Solicited Adverse Events

Solicited AEs as defined in the CSP and in the patient diaries are presented in Table B2 below. Studies COV001 and COV002 have identical terms in the CSPs and patients diaries. Study COV003 terms are synonymous with the terms in Studies COV001 and COV002. Study COV005 has identical or synonymous terms for 12 of the solicited AEs, but some protocol-specified events are not collected in the patient diary (ie, pain, warmth, malaise, nausea, vomiting) and an additional local event, bruising, is collected in the patient diary.

Table B2 CSP-specified Solicited Adverse Events and Patient Diary Terms for these Events by Study

**Adverse Event**	**CSP Terms**			**Patient Diary Terms**		
**Study**	**COV001, COV002**	**COV003**	**COV005**	**COV001, COV002**	**COV003**	**COV005**
Pain	Pain/<br>Pain at injection site	Pain/<br>Injection site pain	Pain/<br>Pain at injection site	Pain	Pain	-
Tenderness	Tenderness	Sensitivity	Tenderness	Tenderness	Sensitivity	Tenderness
Redness	Erythema at injection site	Redness/<br>Injection site erythema	Redness	Redness	Redness	Redness
Warmth	Warmth	Heat	Warmth	Warmth	Local heat	-
Itch	Itch	Itching	Itch	Itching	Itch	Itching
Swelling	Swelling	Swelling	Swelling	Swelling	Swelling	Swelling
Induration	Induration	Local hardening	Induration	Hardness	Local hardening	Hardness
Fever	Fever	Fever	Fever	Temperature	Temperature	Temperature
Feverishness	Feverishness	Feeling feverish	Feverishness	Feverishness	Feeling feverish	Feeling feverishness
Chills	Chills	Chills	Chills	Chills	Chills	Rigors
Joint pain	Joint pains	Joint pain	Joint pains	Joint pain	Joint pain	Joint pain
Fatigue	Fatigue	Fatigue	Fatigue	More tired than usual	Fatigue	Weakness/<br>Tiredness
Muscle pain	Muscle pains	Muscle ache	Muscle pain	Aching muscles	Muscle pain	Muscle pain
Headache	Headache	Headache	Headache	Headache	Headache	Headache
Malaise	Malaise	Malaise	Malaise	Generally unwell	Malaise	-
Nausea	Nausea	Nausea	Nausea	Nausea	Nausea	-

**Table B2**	**CSP-specified Solicited Adverse Events and Patient Diary Terms for these Events by Study**					
**Adverse Event**	**CSP Terms**			**Patient Diary Terms**		
**Study**	**COV001, COV002**	**COV003**	**COV005**	**COV001, COV002**	**COV003**	**COV005**
Vomiting	Vomiting	Vomiting	-	Vomiting	Vomiting	-
Bruising	-	-	Bruising	-	-	Bruising

CSP = clinical study protocol.

B 4 Pooling of Solicited Adverse Events with Objective Measurements

Four solicited AEs will be reported with objective measurements: fever (measured by body temperature in degrees Celsius), redness, swelling, and induration (reported in diameter in millimeters).

B 4.1 Fever

All 4 studies collected body temperature in degrees Celsius to the nearest tenth of a degree. In the individual CSPs, Studies COV001, COV002, and COV003 specify the use of the US FDA DMID scale for vaccine studies while Study COV005 specifies the use of the US NIH DAIDS grading scale. The scales are presented in Table B3 below. Note that the life-threatening criteria are identical, but the ranges of the other 3 severity grades overlap by 0.1- 0.3 °C.

Table B3 Fever Severity Grades for the DMID and DAIDS Grading Scales

Severity grade	DMID	DAIDS
Grade 1	38.0 - 38.4°C	38.0 to < 38.6°C
Grade 2	38.5 - 38.9°C	≥38.6 to < 39.3°C
Grade 3	39.0 - 40°C	≥39.3 to < 40.0°C
Grade 4	> 40°C	> 40°C

DAID = Division of AIDS; DMID = Division of Microbiology and Infectious Diseases

As the data collected are objective numerical measurements, the data can be easily pooled. The severity grades of fever from all 4 studies will be assessed based upon the DMID scale specified in Studies COV001, COV002, and COV003 prior to pooling.

B 4.2 Redness, Swelling, and Induration

Redness and swelling will be reported in millimeters in all 4 studies, and all 4 CSPs contain the same severity grading (Table B4). (Note: the source data are recorded in millimeters but the grading criteria use centimeter; unit conversion will be required).

Induration will also be reported in millimeters in Studies COV001, COV002, and COV003 with the same protocol-defined severity grades, in centimeters. However, in Study COV005, induration is reported in the patient diary at 3 severity grade levels (mild, moderate, severe) that are not compatible with the millimeter measurements used in the other 3 studies.

Based upon the above, redness and swelling can be pooled across all 4 studies, and induration can be pooled across Studies COV001, COV002, and COV003.

Table B4 CSP-specified Severity Grading and Patient Diary Measurements for Redness, Swelling, and Induration

Event	CSP-specified grading severity	Patient diary measurement	
Study	COV001, COV002, COV003, COV005	COV001, COV002, COV003	COV005
Redness	Grade 1: 2.5-5 cm	Millimeter	Millimeter
	Grade 2: 5.1-10 cm		
	Grade 3: > 10 cm		
	Grade 4: Necrosis or exfoliative dermatitis		
Swelling	Grade 1: 2.5-5 cm and does not interfere with activity	Millimeter	Millimeter
	Grade 2: 5.1 - 10 cm or interferes with activity		
	Grade 3: > 10 cm or prevents daily activity		
	Grade 4: Necrosis		
Induration	Grade 1: 2.5-5 cm and does not interfere with activity	Millimeter	Grade 1: small hard lumps/swelling felt, smaller than 25 mm
	Grade 2: 5.1 - 10 cm or interferes with activity		Grade 2: hard lump/swelling felt, larger than 25 mm
	Grade 3: > 10 cm or prevents daily activity		Grade 3: large lump/swelling felt, wider than half the arm width
	Grade 4: Necrosis		-

CSP = clinical study protocol.

B 4.3 Bruising

Only Study COV005 collected bruising as a solicited AE. Severity grading within the diary consisted of Grades 1 (< 10 mm), 2 (10-25 mm), and 3 (> 25 mm). For the sake of completeness, these results will be presented within the pooled data output.

B 5 Pooling of Solicited Adverse Events with Subjective Measurements

This section presents events that will be reported by participants using subjective severity grades.

B 5.1 Pain and Warmth at the Injection Site, Malaise, Nausea, and Vomiting

Pain and warmth at the injection site and malaise, nausea, and vomiting will be reported in Studies COV001, COV002, and COV003. (Study COV005 did not include vomiting in the protocol and did not collect pain and warmth at the injection site and malaise and nausea despite being specified in the protocol.) Common severity grades are used in Studies COV001 and COV002 (Table B5). The severity grades in Study COV003 are somewhat different but similar enough to justify pooling of these 4 solicited AE across all 3 studies.

Table B5 Patient Diary Severity Grades for Pain and Warmth at Injection Site, Malaise, Nausea, and Vomiting Events in Studies COV001, COV002, and COV003

Severity	COV001, COV002	COV003
Grade 1	Mild: easily tolerated with no limitation on normal activity	Mild: transient or mild discomfort (< 48 hours); no interference with routine activities; no medical intervention/therapy required
Grade 2	Moderate: some limitation of daily activity	Moderate: mild to moderate limitation in routine activities – some assistance may be needed; no or minimal medical intervention/therapy required
Grade 3	Severe: unable to perform normal daily activity	Severe: marked limitation in routine activities, some assistance usually required; medical intervention/therapy required
Grade 4	Emergency department or hospital admission required	Potentially life-threatening: requires assessment in accident & emergency department.or hospitalization

B 5.2 Feverishness and Chills

Feverishness and chills will be reported with common severity grades in Studies COV001 and COV002, slightly different severity grades in Study COV003, and a binary “yes” or “no” without severity grading in Study COV005 (Table B6). The severity grades used in Studies COV001, COV002, and COV003 are similar enough to justify pooling of these 2 solicited AEs across the 3 studies. The lack of severity grading of these events in Study COV005 precludes them being pooled with the other studies.

Table B6 Patient Diary Severity Grades for Feverishness and Chills in Studies COV001, COV002, and COV003

Severity	COV001, COV002	COV003	COV005
Grade 1	Mild: easily tolerated with no limitation on normal activity	Mild: transient or mild discomfort (< 48 hours); no interference with routine activities; no medical intervention/therapy required	Yes/No (without grading)
Grade 2	Moderate: some limitation of daily activity	Moderate: mild to moderate limitation in routine activities – some assistance may be needed; no or minimal medical intervention/therapy required	
Grade 3	Severe: unable to perform normal daily activity	Severe: marked limitation in routine activities, some assistance usually required; medical intervention/therapy required	
Grade 4	Emergency department or hospital admission required	Potentially life-threatening: requires assessment in accident & emergency department or hospitalization	

B 5.3 Tenderness, Itching, Joint pain, Muscle pain, Fatigue, and Headache

These solicited AEs have identical 4-level severity grading scales in Studies COV001 and COV002 and a slightly different 4-level grading scale in Study COV003 (Table B7). Study COV005 has event-specific criteria and differs further from the other studies in that a 3-point mild, moderate, and severe scale will be used for these events. The severity grades used in Studies COV001, COV002, and COV003, as discussed above, are similar enough to justify pooling of these 6 solicited AEs across all 3 studies. In addition, the 3-level event-specific severity grades used in Study COV005 do define mild, moderate, and severe events and are also judged to be similar enough to justify pooling across studies.

Table B7 Patient Diary Severity Grading for Tenderness, Itching, Joint Pain, Muscle Pain, Fatigue, and Headache by Study

COV001, COV002	COV003	COV005					
**All events**	**All events**	**Tenderness**	**Itching**	**Joint pain**	**Muscle pain**	**Fatigue**	**Headache**
Mild: easily tolerated with no limitation on normal activity	Mild: transient or mild discomfort (< 48 hours); no interference with routine activities; no medical intervention/therapy required	Mild: minor tenderness when injection site is touched	Mild: minor itching	Mild: mild aching	Mild aching	Minor weakness/ tiredness	Minor headache not requiring medication
Moderate: some limitation of daily activity	Moderate: mild to moderate limitation in routine activities – some assistance may be needed; no or minimal medical intervention/therapy required	Moderate: very tender when injection site is touched	Moderate: marked itching (like new mosquito bite)	Moderate: severe aching, but able to do most activities	Severe aching, but able to do most activities	Very weak/tired	Bad headache, but able to do most activities (with medication)
Severe: unable to perform normal daily activity	Severe: marked limitation in routine activities, some assistance usually required; medical intervention/therapy required	Severe: severe pain in the injected limb, increased when it is moved or movement of the limb is reduced	Severe: severe itching requiring soothing cream	Severe: very severe aching, requiring medication, limiting activities	Very severe aching, requiring medication, limiting activities	Unable to do normal activities during the day	Severe headache, requiring medication and unable to do normal activities during the day
Emergency department or hospital admission required	Potentially Life-threatening: requires assessment in accident & emergency department or hospitalization	-	-	-	-	-	-

B 6 Overall Solicited Adverse Event Pooling Strategy

Table B8 Contribution of Individual Studies to the Overall Pool for Solicited Adverse Events

	Data to be included in the pooling by study				Comments
	**COV001**	**COV002**	**COV003**	**COV005**	
Solicited local adverse events					
Pain	X	X	X	NA	Grade based upon Studies COV001/COV002 grading criteria. No data from Study COV005.
Tenderness	X	X	X	X	Grade based upon Studies COV001/COV002 grading criteria. Study COV005 scale lacks Grade 4
Redness	X	X	X	X	Grade based upon common objective grading criteria (diameter)
Warmth	X	X	X	NA	Grade based upon Studies COV001/COV002 grading criteria. No data from Study COV005.
Itch	X	X	X	X	Grade based upon Studies COV001/COV002 grading criteria. Study COV005 scale lacks grade 4
Swelling	X	X	X	X	Grade based upon common objective grading criteria (diameter)
Induration	X	X	X	-	Grade based upon the common objective grading criteria. Study COV005 grading scale not compatible.
Bruising	NA	NA	NA	X	Included despite recorded in only one study so that all available data are included.
Solicited systemic adverse events					
Fever	X	X	X	X	Grade based upon Studies COV001/COV002/COV003 grading criteria.
Feverishness	X	X	X	-	Grade based upon Studies COV001/COV002 grading criteria. Study COV005 has no severity grading
Chills	X	X	X	-	Grade based upon Studies COV001/COV002 grading criteria. Study COV005 has no severity grading.
Joint pain	X	X	X	X	Grade based upon Studies COV001/COV002 grading criteria. Study COV005 scale lacks Grade 4
Muscle pain	X	X	X	X	Grade based upon Studies COV001/COV002 grading criteria. Study COV005 scale lacks

Table B8 Contribution of Individual Studies to the Overall Pool for Solicited Adverse Events

	Data to be included in the pooling by study				Comments
	**COV001**	**COV002**	**COV003**	**COV005**	
					Grade 4
Fatigue	X	X	X	X	Grade based upon Studies COV001/COV002 grading criteria. Study COV005 scale lacks Grade 4
Headache	X	X	X	X	Grade based upon Studies COV001/COV002 grading criteria. Study COV005 scale lacks Grade 4
Malaise	X	X	X	NA	Grade based upon Studies COV001/COV002 grading criteria. No data from Study COV005.
Nausea	X	X	X	NA	Grade based upon Studies COV001/COV002 grading criteria. No data from Study COV005.
Vomiting	X	X	X	NA	Grade based upon Studies COV001/COV002 grading criteria. No data from Study COV005.

NA = not available.

Appendix C Pooling and Severity Grading of Laboratory Assessed on Scheduled Visits

In Studies COV001, COV002, and COV005, the severity of laboratory toxicity from scheduled assessments will be assessed on scales of Grades 1-4. In Studies COV001 and COV002, these laboratory abnormalities will be assessed according to grading scales that have been adapted from the US FDA Toxicity Grading Scale for Healthy and Adolescent Volunteers Enrolled in Preventive Vaccine Clinical Trials (FDA 2007), using identical grading scales. In Study COV005, laboratory toxicity will be assessed using grading scales adapted from the DAIDS Table for Grading the Severity of Adult and Pediatric Adverse Events, Corrected Version 2.1 (NIH 2017).

In order to pool laboratory abnormalities from these studies, all pooled data will be uniformly graded using the US FDA grading system as the common grading system. (Note: Study COV003 does not contribute data to this pooling as the study does not have scheduled laboratory assessments.) For the purposes of reporting, values which do not meet at least the criteria of Grade 1 will be reported as normal, to account for results falling between the upper limit of normal for individual laboratories and the lower limit for Grade 1 toxicity. For those variables in which US FDA severity gradings are based upon multiples of the upper limit of the normal reference range (ie, alanine transaminase, alkaline phosphate, and bilirubin), study specific reference ranges will be used.

Table C1 presents the severity gradings that will be used for pooled laboratory abnormalities from Studies COV001, COV002, and COV005. Table C2 presents a comparison of the US FDA severity grade criteria with the 3 studies' individual criteria.

Table C1 Laboratory Abnormality Severity Grade Criteria to be Used for Pooled COV1, COV002, and COV005 Data

Variable	Unit	Normal Range	Grade 1	Grade 2	Grade 3	Grade 4	Comments
Haemoglobin Absolute Decreased (male)	g/L	-	125-135	105-124	85-104	<85	FDA values.
Haemoglobin Absolute Decreased (female)	g/L	-	110-120	95-109	80-94	<80	FDA values.
Haemoglobin Decrease from Baseline	g/L	-	1-15	16-20	21-50	>50	FDA values, Consistent with COV001, COV002, except lower limit for Grade 1
White Blood Cells-Elevated	cells × 10^9/L	-	10.8-15.0	>15.0-20.0	>20.0-25.0	>25.0	FDA values. Consistent with COV001, COV002, except lower limit for Grade 1
White Blood Cells-Decreased	cells × 10^9/L	-	2.5-3.5	1.5-<2.5	1.0-<1.5	<1.0	FDA values. COV001, COV002
Platelets-Decreased	cells × 10^9/L	-	125-140	100-124	25-99	<25	FDA values. COV001, COV002
Neutrophils-Decreased	cells × 10^9/L	-	1.50-2.00	1.00-1.49	0.50-0.90	<0.50	FDA values. COV001, COV002
Lymphocytes-Decreased	cells × 10^9/L	-	0.750-1.000	0.500-0.749	0.250-0.499	<0.250	FDA values. COV001, COV002
Eosinophils-Elevated	cells × 10^9/L	-	0.650-1.500	1.501-5.000	>5.000	-	FDA values. COV001, COV002
Sodium-Elevated	mmol/L	-	144-145	146-147	148-150	>150	FDA values
Sodium-Decreased	mmol/L	-	132-134	130-131	125-129	>125	FDA values. COV001, COV002
Potassium-Elevated	mmol/L	-	5.1-5.2	5.3-5.4	5.5-5.6	>5.6	FDA values
Potassium-Decreased	mmol/L	-	3.5-3.6	3.3-3.4	3.1-3.2	<3.1	FDA values.

Table C1 Laboratory Abnormality Severity Grade Criteria to be Used for Pooled COV1, COV002, and COV005 Data

Variable	Unit	Normal Range	Grade 1	Grade 2	Grade 3	Grade 4	Comments
Urea-Elevated (*converted from BUN mg/dL)*	mmol/L	-	8.2-9.4	9.5-11.0	>11.0	-	FDA values. COV001, COV002
Creatinine-Elevated (*converted from mg/dL)*	µmol/L	-	133-154	155-181	182- 221	>221	FDA values
Bilirubin-Elevated with normal ALT/ALP	µmol/L	COV001/ COV002: 0-21 COV005: 5-21	1.1-1.5 × ULN	1.6-2.0 × ULN	2.0-3.0 × ULN	>3.0 × ULN	FDA values. COV001, COV002. Country specific normal ranges.
Bilirubin-Elevated with abnormal ALT/ALP	µmol/L		1.1-1.25 × ULN	1.26-1.5 × ULN	1.51-1.75 × ULN	>1.75 × ULN	FDA values. COV001, COV002. Country specific normal ranges.
Alanine Transaminase-Elevated	U/L	COV001/ COV002: 10-45 COV005: 15-40	1.1-2.5 × ULN	2.6-5.0 × ULN	5.1-10 × ULN	>10 × ULN	FDA values. COV001, COV002. Country specific normal ranges.
Alkaline Phosphate-Elevated	U/L	COV001, COV002: 30-130 COV005: 53-128	1.1-2.0 × ULN	2.1-3.0 × ULN	3.1-10 × ULN	>10 × ULN	FDA values. COV001, COV002. Country specific normal ranges.
Albumin-Decreased	g/L	-	28-31	25-27	<25	-	FDA values. COV001, COV002.

ALP = alkaline phosphate; ALT = alanine aminotransferase; BUN = blood urea nitrogen; FDA = (US) Food and Drug Administration; N.A. = not available; ULN = upper limit of normal.

Table C2 Laboratory Abnormality Severity Grade Criteria to be Used for Pooled COV001, COV002, and COV005 Data

Variable	Unit	Standard	Normal Range	Grade 1	Grade 2	Grade 3	Grade 4
Haemoglobin Absolute Decreased (male)	g/L	FDA	-	125-135	105-124	85-104	<85
		COV001 COV002	130-170	115-125	100-114	85-99	<85
		COV005 (DAIDS)	-	100-109	90-<100	70-<90	<70
Haemoglobin Absolute Decreased (female)	g/L	FDA	-	110-120	95-109	80-94	<80
		COV001 COV002	120-150	105-113	90-104	80-89	<80
		COV005 (DAIDS)	-	95-104	85-<95	65-<85	<65
Haemoglobin Decrease from Baseline	g/L	FDA	-	1-15	16-20	21-50	>50
		COV001 COV002	-	10-15	16-20	21-50	>50
		COV005 (DAIDS)	-	N.A.	N.A.	N.A.	N.A.
White Blood Cells-Elevated	cells × 10^9/L	FDA	-	10.8-15	>15-20	>20-25	>25
		COV001 COV002	4.0-11	11.5-15	>15-20	>20-25	>25
		Cov005 (DAIDS)	-	N.A.	N.A.	N.A.	N.A.
White Blood Cells-Decreased	cells × 10^9/L	FDA	-	2.5-3.5	1.5-2.49	1.0-1.49	<1.0
		COV001 COV002	4.0-11	2.5-3.5	1.5-2.49	1.0-1.49	<1.0
		Cov005 (DAIDS)	-	2.00-2.49	1.50-1.99	1.00-1.49	<1.0
Platelets-Decreased	cells × 10^9/L	FDA	-	125-140	100-124	25-99	<25
		COV001 COV002	150-400	125-140	100-124	25-99	<25
		COV005 (DAIDS)	-	100-< 125	50-<100	25-<50	< 25
Neutrophils-Decreased	cells × 10^9/L	FDA	-	1.5-2.00	1.0-1.49	0.5- 0.99	<0.50
		COV001 COV002	2.0-7.0	1.5-1.99	1.0-1.49	0.5-0.99	<0.50
		COV005 (DAIDS)	-	0.80-1.00	0.60-0.79	0.40-0.59	<0.40
Lymphocytes-Decreased	cells ×	FDA	-	0.750-1.000	0.500-0.749	0.250-0.499	<0.250

Table C2 Laboratory Abnormality Severity Grade Criteria to be Used for Pooled COV001, COV002, and COV005 Data

Variable	Unit	Standard	Normal Range	Grade 1	Grade 2	Grade 3	Grade 4
	10^9/L	COV001 COV002	1.0-4.0	0.75-0.99	0.5-0.74	0.25-0.49	<0.25
		COV005	-	0.60-0.65	0.500- 0.599	0.350-0.499	<0.350
Eosinophils-Elevated	cells × 10^9/L	FDA	-	0.650-1.500	1.501-5.000	>5.000	Hypereosinophilic
		COV001 COV002	0.02-0.5	0.65-1.5	1.51-5.00	>5.00	Hypereosinophilic
		COV005	-	N.A.	N.A.	N.A.	N.A.
Sodium-Elevated	mmol/L	FDA	-	144-145	146-147	148-150	>150
		COV001 COV002	134-145	146-147	148-149	150-155	>155
		COV005 (DAIDS)	-	146 to < 150	150 to < 154	154 to < 160	≥ 160
Sodium-Decreased	mmol/L	FDA	-	132-134	130-131	125-129	<125
		COV001 COV002	135-145	132-134	130-131	125-129	<125
		COV005 (DAIDS)	-	130-< 135	125-< 130	121-< 125	≤120
Potassium-Elevated	mmol/L	FDA	-	5.1-5.2	5.3-5.4	5.5-5.6	>5.6
		COV001 COV002	3.5-5	5.1-5.2	5.3-5.4	5.5-6.5	>6.5
		COV005 (DAIDS)	-	5.6-< 6.0	6.0-< 6.5	6.5-< 7.0	≥ 7.0
Potassium-Decreased	mmol/L	FDA	-	3.5-3.6	3.3-3.4	3.1-3.2	<3.1
		COV001 COV002	-	3.2-3.3	3.1	2.5-3.0	<2.5
		COV005 (DAIDS)	-	3.0-< 3.4	2.5-< 3.0	2.0-< 2.5	< 2.0
Urea-Elevated (*converted from BUN mg/dL)*	mmol/L	FDA	-	8.2-9.3	9.4-11.0	>11.0	Requires dialysis
		COV001 COV002	2.5-7.4	8.2-9.3	9.4-11.0	>11.0	Requires dialysis
		COV005 (DAIDS)	-	N.A.	N.A.	N.A.	N.A.
Creatinine-Elevated (*converted from mg/dL)*	mg/dL (μmol/L)	FDA	-	1.5-1.7 (133-154)	1.8-2.0 (155-181)	2.1-2.5 (182-221)	>2.5 (>221) or requires dialysis

Table C2 Laboratory Abnormality Severity Grade Criteria to be Used for Pooled COV001, COV002, and COV005 Data

Variable	Unit	Standard	Normal Range	Grade 1	Grade 2	Grade 3	Grade 4
		COV001 COV002	49-104	1.1-1.5 × ULN<br>114-156	>1.5-2.0 × ULN<br>157-312	>2.0 × ULN<br>>312	Requires dialysis
		COV005	64-10	1.1-1.3 × ULN	> 1.3-1.8 × ULN	> 1.8-< 3.5 × ULN	≥ 3.5 × ULN
Bilirubin-Elevated	µmol/L	FDA with normal ALT/ALP	-	1.1-1.5 × ULN	1.6-2.0 x ULN	2.0-3.0 × ULN	>3.0 × ULN
		FDA with abnormal ALT/ALP		1.1-1.25 × ULN	1.26-1.5 × ULN	1.51-1.75 × ULN	>1.75 × ULN
		COV001 COV002 -Normal LFTs	0-21	1.1-1.5 × ULN<br>23-32	>1.5-2 × ULN<br>33-42	>2-3 × ULN<br>43-63	>3 × ULN<br>>64
		COV005 -Normal LFTs	5-21				
		COV001 COV002 -Abnormal LFTs	0-21	1.1-1.25 × ULN<br>23-26	>1.25-1.5 × ULN<br>27-32	>1.5-1.75 × ULN<br>33-37	>1.75 × ULN<br>>37
		COV005 -Abnormal LFTs	5-21				
		COV005 (DAIDS)	-	1.1-< 1.6 × ULN	1.6-< 2.6 × ULN	2.6-< 5.0 × ULN	≥ 5.0 × ULN
Alanine Transaminase-Elevated	U/L	FDA		1.1-2.5 ULN	2.6-5 × ULN	5.1-10 × ULN	>10 × ULN
		COV001 COV002	10-45	1.1-2.5 × ULN<br>49-112	>2.5-5 × ULN<br>113-225	>5-10 × ULN<br>226-450	>10 × ULN<br>>450
		COV005 (DAIDS)	15-40	1.25-< 2.5 × ULN	2.5-< 5.0 × ULN	5.0-< 10.0 × ULN	≥10.0 × ULN
Alkaline Phosphate-	µmol/L	FDA		1.1-2.0 × ULN	2.1-3.0 × ULN	3.1-10 × ULN	>10 × ULN

Table C2 Laboratory Abnormality Severity Grade Criteria to be Used for Pooled COV001, COV002, and COV005 Data

Variable	Unit	Standard	Normal Range	Grade 1	Grade 2	Grade 3	Grade 4
		COV001 COV002	30-130	1.1-2 × ULN<br>143-260	>2.-3 × ULN<br>261-390	>3-10 × ULN<br>391-1300	>10 × ULN<br>>1300
		COV005 (DAIDS)	53-128	1.25-< 2.5 × ULN	2.5-< 5.0 × ULN	5.0-< 10.0 × ULN	≥ 10.0 × ULN
Albumin-Decreased	µmol/L	FDA	-	28-31	25-27	<25	N.A.
		COV001 COV002	32-50	28-31	25-27	<25	N.A.
		COV005 (DAIDS)	-	30-<LLN	≥20-<30	<20	N.A.

ALP = alkaline phosphate; ALT = alanine aminotransferase; BUN = blood urea nitrogen; DAIDS = Division of AIDS; FDA = (US) Food and Drug Administration; N.A. = not available; LFT = liver function tests; LLN = lower limit of normal; ULN = upper limit of normal
